# A biomimetic, ultralow-power edge-AI-empowered and self-sustaining gait analysis system

**DOI:** 10.1126/sciadv.aeh9625

**Published:** 2026-08-19

**Authors:** Fuying Dong, Chi Han, Pengchong Xu, Jasleen Chhatwal, Xinnian Jiang, Tengteng Wang, Abigail Hsu, Minzhu Baek, Di Wu, Rui Li, Yuanwen Jiang, Bozhi Tian, Jason Y. Fang, Simiao Niu

**Affiliations:** ^1^ Department of Biomedical Engineering, Rutgers University; Piscataway, New Jersey 08854, United States.; ^2^ Department of Electrical and Computer Engineering, Rutgers University; Piscataway, New Jersey 08854, United States.; ^3^ Department of Medicine, Division of Medical Oncology, Section of Cancer Epidemiology and Health Outcomes, Rutgers Robert Wood Johnson Medical School, Rutgers Cancer Institute; New Brunswick, New Jersey 08901, United States.; ^4^ Department of Chemical and Materials Engineering, University of California Merced; Merced, California 95343, United States.; ^5^ Tandon School of Engineering, New York University; Brooklyn, New York 11201, United States.; ^6^ Department of Materials Science and Engineering, University of Pennsylvania; Philadelphia, Pennsylvania 19104, United States.; ^7^ Department of Chemistry, University of Chicago; Chicago, Illinois 60637, United States.

## Abstract

Smart digital health has reshaped patient monitoring, but it faces a fundamental trade-off between device intelligence and continuous, energy-efficient monitoring. Inspired by self-sustaining intelligent biospecies, we develop a biomimetic, battery-free, and high-precision edge-AI system through a harvested-energy-constrained holistic co-design that couples ultralow-power edge-AI-empowered sensor hardware with biomechanical energy harvesting and cold-start power management. Our edge-AI-empowered motion sensor performs instantaneous, context-aware on-device inference and timely result updating from raw sensor data while consuming only 86 μW. A high-output energy harvester and tailored high-efficiency power management circuitry sustain energy levels exceeding system requirements, eliminating downtime associated with charging and enabling true 24/7, hassle-free monitoring. This breakthrough establishes a paradigm for system-level, edge-AI-empowered, and self-sustaining sensing, demonstrating that intelligence and energy autonomy can coexist within a single wearable platform and pointing to next-generation always-on, personalized digital health systems.

## INTRODUCTION

The emergence of smart digital health technologies, such as smartwatches, has transformed patient monitoring by enabling the continuous acquisition of physiological signals and providing timely alerts and feedback (*1*–*21*). These capabilities have demonstrated strong potential in improving disease management, reducing unnecessary physician visits, motivating positive lifestyle changes, and increasing patient engagement and adherence to treatment. Smart digital health exists at the intersection of two worlds governed by fundamentally different rules: the biological world, which senses and processes information through slow, low-power, and adaptive mechanisms in a self-sustaining manner, and the digital world, where inference requires rapid, energy-intensive computation. The collision of these regimes gives rise to a rarely articulated but universal constraint in digital health: the intelligence–energy bottleneck, which links the fidelity of physiological inference to the energetic cost of extracting meaning from continuous, high-dimensional biosignals. To provide digital health intelligence and move toward accurate forecasting of patient health trajectories, edge artificial intelligence (edge-AI) must be integrated directly into devices, which enables low-latency, high-precision inference and supports the delivery of personalized, adaptive, instant, and context-aware feedback (*22*, *23*). Nevertheless, the current generation of edge-AI algorithms typically requires high computational power (*23*–*25*), which leads to shortened battery life and interrupted monitoring in smart digital devices.

**Fig. 1.**
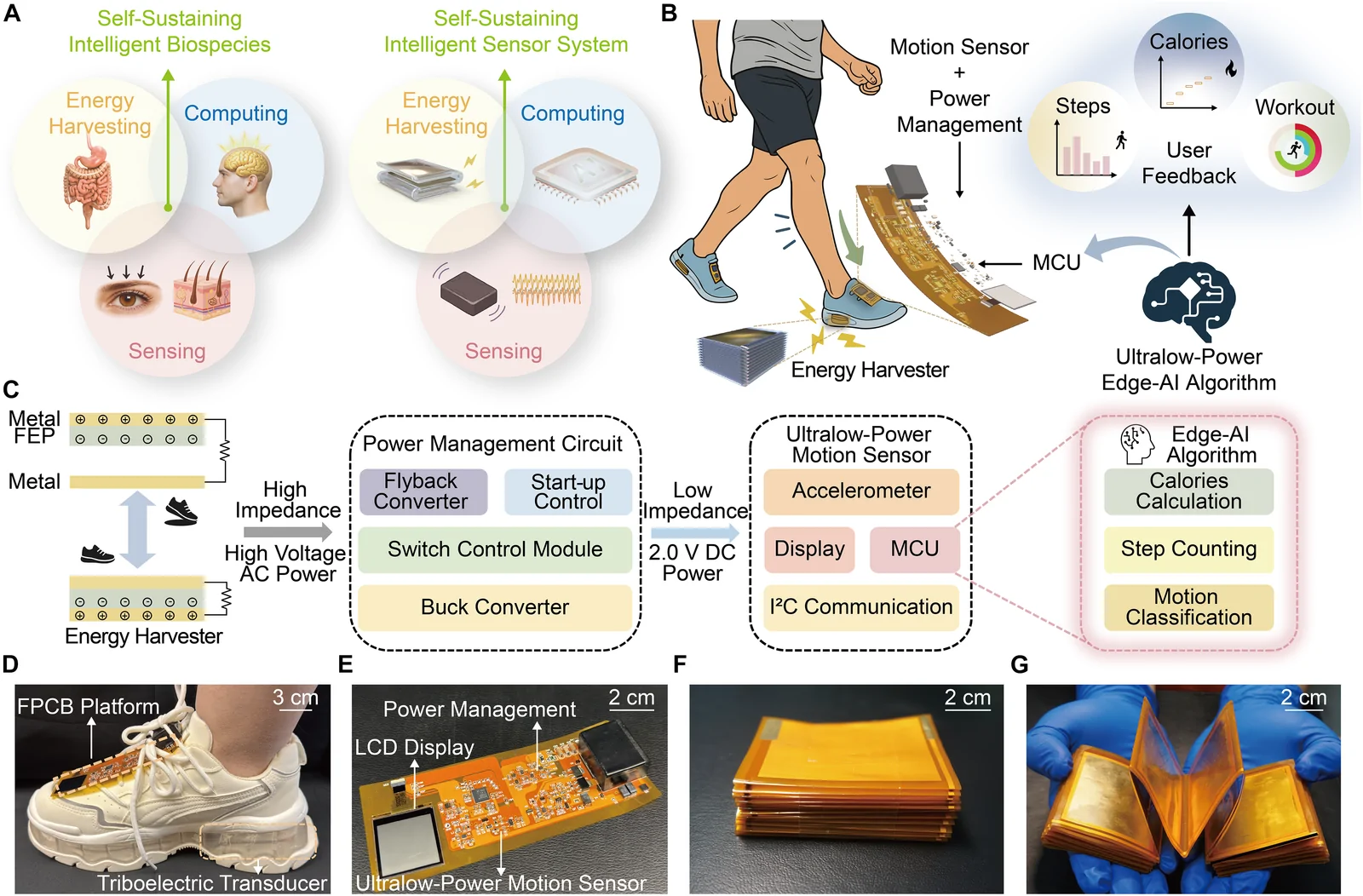
Design of the edge-AI-empowered wearable gait analysis system. (**A**) Bio-inspired design rationale. (**B**) Conceptual schematic of the bio-inspired edge-AI-empowered gait analysis system. (**C**) Conceptual illustration and exploded structural view of the edge-AI-empowered wearable gait analysis system, showing the system workflow from the triboelectric energy harvester to the power management circuit (PMC), ultralow-power (ULP) motion sensor, and edge-AI gait analysis algorithm. (**D**) Photographs of the fully integrated smart shoe system. (**E**) Flexible printed circuit board (FPCB) integrating the PMC and ULP motion sensor modules. (**F**) Photograph of the fabricated triboelectric transducer before attachment to the shoe sole. (**G**) Partially unfolded view of the triboelectric transducer.

Biological systems naturally circumvent this bottleneck by tightly coupling sensing, energy harvesting, and computing (Fig. 1A). For example, the hypothalamus harvests blood glucose as an energy source through the metabolic process, incorporates multiple physiological sensors (glucose-sensing neurons, etc.), and local neural circuits perform ultralow-power inference. This self-sustaining integrated architecture, which co-evolves sensing, ultralow-power computation, and energy harvesting, provides a powerful biomimetic template for next-generation self-sustaining intelligent wearables. Inspired by this, we develop a self-sustaining and high-precision edge-AI system (fig. S1). Here, we define wearable edge-AI sensor systems as wearable sensing platforms that acquire physiological signals and perform AI-based inference locally on embedded hardware, rather than relying solely on continuous raw-data transmission to an external computer, smartphone, or cloud processor. The key innovation behind this platform is the holistic co-design of an ultralow-power edge-AI inference engine and a high-power energy harvesting system. At the heart of the system is energy-optimized sensing hardware tightly coupled with an ultralow-power edge-AI algorithm. This edge-AI algorithm mirrors the energy-frugal computation of neural circuits and enables instantaneous, high-precision, context-aware on-device inference and timely result updating from raw high-dimensional time-series sensor data with a power consumption of only 86 μW. Compared with representative wearable edge-AI sensor systems summarized in table S1, which typically operate in the 10–100 mW range (*23*–*25*), this μW-level power consumption represents an approximately two-order-of-magnitude reduction while enabling local AI inference in a self-sustaining wearable platform. Such an unprecedented level of energy efficiency enables continuous, on-device intelligence at the extreme edge. A high-output energy-harvesting transducer and tailored high-efficiency power management circuitry mirror the metabolic mechanism within biological systems, generating energy levels that exceed the requirements of our edge-AI-empowered sensor systems. This design renders the entire platform battery-free and self-sustaining. It eliminates charging downtime, enables true 24/7, hassle-free monitoring, and could support the future generation of long-term physiological datasets for health monitoring and outcome analysis. This biologically inspired convergence of sensing, computation, and energy harvesting elevates the platform from a traditional wearable to a cyber-biophysical analog of human hypothalamic systems.

To validate the feasibility and versatility of our architecture, we implement this architecture in a gait-sensing system (Fig. 1B). We choose gait monitoring as a demonstration for several compelling reasons. First, gait represents one of the most fundamental expressions of human mobility, integrating intrinsic physiological regulation with external mechanical adaptation to produce fluid, coordinated, and purposeful motion (*26*–*28*). It is a critical biomarker for a spectrum of disorders, from neurological conditions to musculoskeletal and metabolic pathogenesis (*29*, *30*), including cystic fibrosis (*31*), Parkinson’s disease (*32*), spinal cord injury (*33*), rare bone diseases (*34*), etc. (*35*). Second, ultralow-power edge-AI is indispensable for processing the complex, time-series, and high-dimensional raw sensor data generated during locomotion. By performing real-time analysis directly on the device, the edge-AI inference engine transforms the raw signals into clinically interpretable and actionable insights, including step count, gait pattern recognition, and calorie estimation—all without reliance on cloud connectivity or external computing resources. This approach enables instantaneous on-device inference and timely result updating while drastically reducing latency and energy demand. Third, every gait cycle inherently produces low-frequency biomechanical energy, providing a natural and sustainable power source for the system to operate continuously and autonomously without the need for batteries or external charging (*36*, *37*). Our biomimetic, edge-AI-empowered, and self-sustaining gait sensing system (Fig. 1C) incorporates a high-performance energy harvester, a highly efficient cold-start power management circuit (PMC), an ultralow-power accelerometer-based motion sensor, and an embedded ultralow-power edge-AI algorithm. The central contribution of this work is a harvested-energy-constrained system co-design that closes the loop among biomechanical energy generation, cold-start energy regulation, ultralow-power motion sensing, and deployable on-device inference. Rather than optimizing these modules independently, this platform jointly coordinates the energy harvester, PMC, sensing module, and compressed edge-AI model so that gait-triggered harvested energy can support local gait monitoring, on-device analysis, and local feedback during locomotion-powered operation. Moreover, this modular system framework provides a potential route toward scalable and generalizable self-sustaining wearable sensing beyond the present gait-monitoring demonstration. Within gait analysis, the platform can be extended to different users and patient populations by retraining and validating the embedded edge-AI algorithm with population-specific gait datasets. More broadly, the energy harvester can be repositioned or redesigned to collect biomechanical energy from other periodic body motions, and the PMC can be adapted to regulate other intermittent energy-harvesting inputs. The sensing module can also be replaced with other low-power physiological sensors, such as electrocardiogram (ECG) monitoring or electrochemical biosensors, while the embedded edge-AI algorithm can be retrained to extract task-specific physiological or health-related outputs. This work, therefore, establishes a paradigm for system-level, edge-AI-empowered, and self-powered physiological sensing, demonstrating that intelligence and energy autonomy can coexist within a single wearable platform. It opens a transformative pathway toward the next generation of intelligent, always-on, and truly personalized digital health systems.

## RESULTS

### Design of the overall edge-AI-empowered and battery-free system architecture

Our edge-AI-empowered and battery-free gait analysis system integrates four meticulously designed subsystems: (i) a high-performance, fully integrated triboelectric transducer for biomechanical energy harvesting, (ii) a high-efficiency and cold-start PMC to regulate and condition the harvested energy, (iii) an ultralow-power (ULP) motion sensor module, and (iv) a ULP, memory-efficient, and high-precision edge-AI algorithm embedded inside our microcontroller unit (MCU) (Fig. 1C). Structurally, the energy harvester is embedded in a shoe’s air cushion, while the PMC and ULP motion sensor modules (including an edge-AI algorithm) are assembled onto a flexible printed circuit board (FPCB) mounted securely on the shoe’s upper (Fig. 1, D to G). During walking, periodic compression and rebound of the transducer upon ground contact generate pulsed current, which is conditioned by the PMC to transition the system from a zero-energy state and power the motion sensor module and the edge-AI algorithm. As long as gait motion persists, the harvested biomechanical energy continuously sustains system operation.

### Design of the high-output integrated energy harvester and high-efficiency cold-start power management circuit

**Fig. 2.**
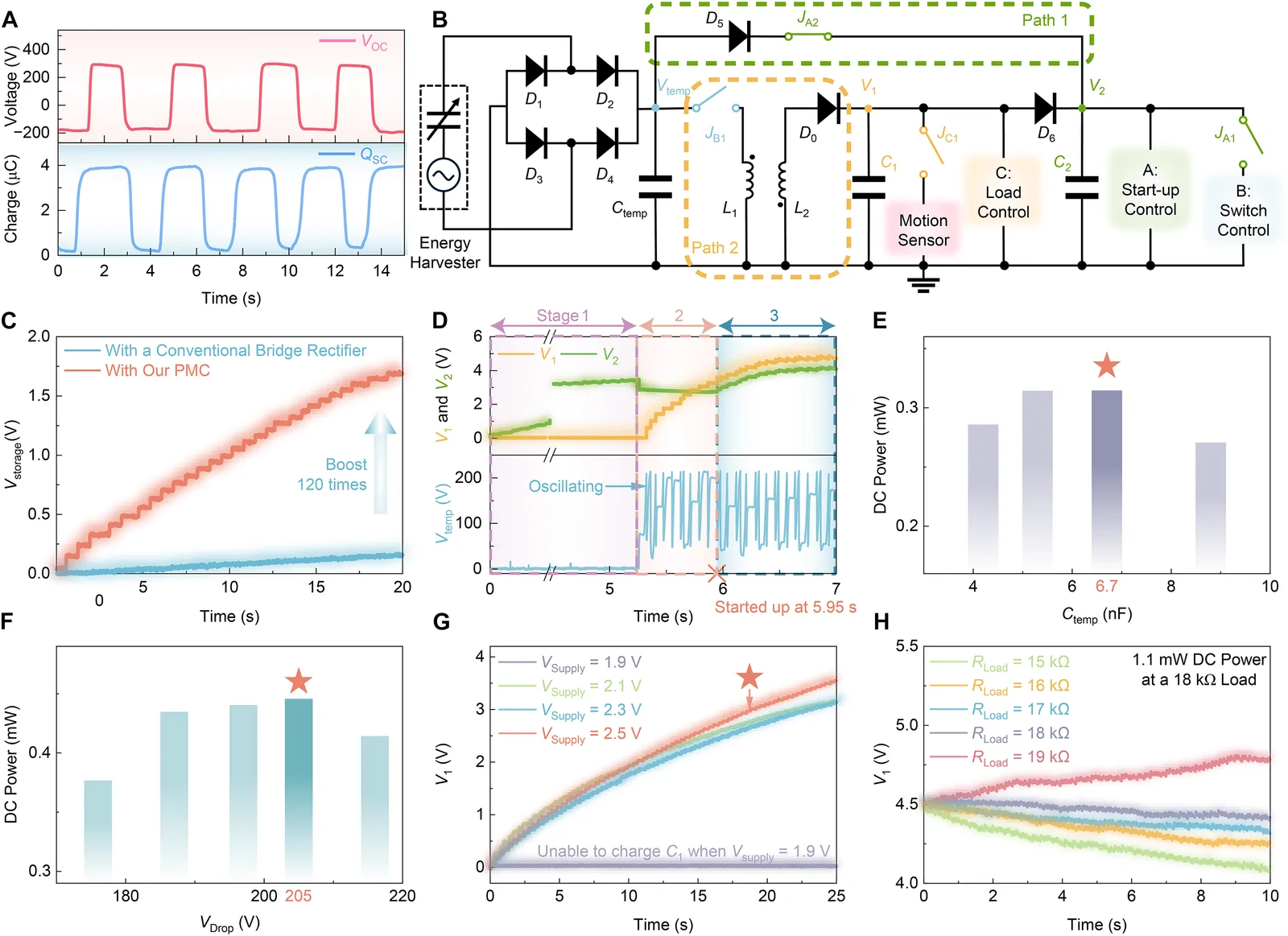
Design and characterization of the power management circuit for regulated energy harvester outputs. (**A**) Output characteristics of the triboelectric transducer, including short-circuit transferred charges (*Q*_SC_) and open-circuit voltage (*V*_OC_). A 5 Hz low-pass FIR filter is applied to remove the power line noise. (**B**) Schematic of the power management circuit. (**C**) Comparison of the designed PMC with conventional diode-bridge rectification for harvesting AC energy. *V*_1_ represents the storage-capacitor voltage measured in each configuration, as labeled in Fig. 2B for the proposed PMC and in fig. S14 for the conventional diode-bridge rectification control, under matched storage-capacitor conditions. (**D**) “Dual-path” self-starting process, including working stages 1–3 that illustrate circuit functionality. Experiments were conducted with *C*_1_ = 100 μF and *C*_2_ = 22 μF during a maximum-effort tapping process, and data were recorded using an oscilloscope. A moving-window average filter was applied to the raw signal to reduce noise and recover the true waveform. (**E**) Variation in DC power output with different *C*_temp_ values. Experiments were conducted with a 2.5 V switch control supply voltage during a 60 beats per minute (BPM) tapping process. (**F**) Variation in DC power output with different flyback switch control voltages, *V*_drop_. Experiments were conducted with a 2.5 V switch control supply voltage during a 90 BPM tapping process. (**G**) Charging behavior of *C*_1_ under different switch control supply voltages. Experiments were conducted with *C*_1_ = 1 mF and *C*_2_ = 100 μF during a continuous 90 BPM tapping process, and data were recorded using an oscilloscope. A moving-window average filter was applied to the raw signal to reduce noise. (**H**) The maximum DC power output of the system driven by human biomechanical energy. Experiments were conducted with *C*_1_ = 1 mF and *C*_2_ = 100 μF during a maximum-effort tapping process.

The energy harvester aims to scavenge low-frequency biomechanical energy from gait cycles. We choose a multi-layer attached-electrode contact-mode triboelectric transducer design (fig. S2, Fig. 1, E and F), as this is the most suitable for harvesting low-frequency gait energy compared to other transducer types (*38*–*43*). Next, we adopt an FPCB-based fabrication technique (fig. S3), which substantially reduces fabrication complexity (eliminating the need for fragile external wire connections between layers; fig. S4) and total weight (by 38.3%). Device performance and durability are further optimized through the geometric design (figs. S3, S6, and S7). We realize 3.8 μC of short-circuit transferred charge and 475 V open-circuit voltage (Fig. 2A, figs. S8 to S13), while maintaining a compact profile (80 by 58 by 22 mm^3^/53.3 g).

Besides the high-output energy harvester, we design a PMC (Fig. 2B) that enhances the harvested DC energy by up to 120 times (Fig. 2C) compared to a conventional full-wave bridge rectifier (fig. S14). Compared to our previous design (*10*), we add a cold-start module to the PMC to address its initial start-up issue. This cold-start capability allows the PMC to initiate operation when no energy is initially stored in the system, without requiring a pre-charged storage element, battery, or external power supply. Without it, the PMC cannot start from a completely uncharged state because its internal control circuitry requires a power supply. The cold-start module first provides a passive path to accumulate start-up energy, then enables the PMC to transition to the optimized energy-extraction mode. This cold-start module consists of a relatively small start-up capacitor *C*_2_, two electronic switches (*J*_A1_ and *J*_A2_), a start-up control logic, and two diodes (*D*_5_ and *D*_6_) (Fig. 2B). The capacitor *C*_2_ is designed to provide the initial power for the flyback switch control circuit (FSCC) when the voltage of *C*_1_ (*V*_1_, that powers the final load) is below a threshold. The whole PMC operates on a “dual-path” approach. Path 1 employs the depletion-mode MOSFET transistor (*J*_A2_) to bypass the flyback converter and passively charge *C*_2_. Path 2, in contrast, uses an active flyback converter, enabling substantially higher energy conversion efficiency. Three hysteresis-comparator-based control circuits are designed to control the electronic switches within the PMC. First, the start-up control circuits (SUCC) switch between Path 1 and 2 (*J*_A1_ and *J*_A2_) based on the voltage level of *C*_2_ (*V*_2_). Second, the FSCC regulates flyback converter operation (*J*_B1_) based on the *C*_temp_ voltage. Third, the load control activates the motion sensor circuitry (*J*_C1_) when *V*_1_ reaches a predesigned threshold.

The overall operational sequence can be divided into 4 distinct stages (Fig. 2D, figs. S15 and S16). The key purpose of this staged process is to first generate a small amount of start-up energy via a passive charging path (Path 1) and then use it to activate the higher-efficiency flyback charging path (Path 2). At the beginning (stage 0), no energy is stored in the system (*V*_1_ and *V*_2_ are 0). The switch *J*_A2_ is designed to be closed since it is a depletion-mode NMOS transistor (Fig. 2B). In contrast, the switches *J*_B1_ and *J*_A1_ are open because they are enhancement-mode NMOS transistors. When the energy harvester generates electricity (Stage 1 – Non-optimized Direct Charging, 0–5.2 s, shown in Fig. 2D), as *J*_A2_ is closed and *J*_B1_ is open, the energy generated by the harvester flows directly through Path 1 to *C*_2_ via a bridge rectifier, and *V*_2_ increases (fig. S16A). Then, when *V*_2_ reaches the rising threshold of *C*_2_ (*V*_thr2_) (5.2 s in Fig. 2D), the SUCC opens *J*_A2_ and closes *J*_A1_, switching the energy flow from Path 1 to Path 2. Subsequently, the FSCC is supplied by *C*_2_, and a stable voltage of 2.5 V is generated to control the flyback converter switch *J*_B1_ (Stage 2 – Optimized Flyback Charging, from 5.2 s to 5.95 s in Fig. 2D). The harvested energy is now transferred through the flyback converter in Path 2 to charge the main storage capacitor *C*_1_, leading to an increase in *V*_1_ (fig. S16B). Since *C*_2_ is powering the FSCC during Stage 2 and is not being charged from the harvester, *V*_2_ will gradually decrease. Once *V*_2_ falls below the falling threshold of *C*_2_ (*V*_thf2_), the SUCC detects this change and reverts to Stage 1 to recharge *C*_2_ by opening *J*_A1_ and closing *J*_A2_, allowing *C*_2_ to be recharged through Path 1. This alternating operation between Stage 1 and Stage 2 continues until *V*_1_ reaches *V*_2_ + *V*_fwd_ (where *V*_fwd_ is the forward voltage drop of diode *D*_6_). At this time (Stage 3, after 5.95 s in Fig. 2D), *C*_2_ can be charged by *C*_1_ through *D*_6_ (fig. S16C) and no longer requires repeated passive start-up charging from Path 1. The cold-start process is therefore completed, and the PMC remains in the optimized flyback conversion configuration (Path 2), thereby optimizing power extraction from the energy harvester. When *V*_1_ reaches the rising threshold of *C*_1_ (*V*_thr1_), the load control closes *J*_C1_ and starts the motion sensor unit.

We further optimize the PMC’s functional components to reach the desired performance. First, we tune both *C*_temp_ and *V*_drop_ to maximize the flyback converter’s power transfer efficiency. An appropriate selection of *C*_temp_ minimizes energy dissipation. Optimal performance is achieved when *C*_temp_ is ∼6.7 nF, yielding 0.31 mW DC power at a tapping frequency of 60 BPM (Fig. 2E). In contrast, the design of *V*_drop_ is based on the impedance match between the energy harvester and the PMC. By modifying the hysteresis window of the FSCC, an optimum value of *V*_drop_ is measured to be ∼205 V, resulting in 0.44 mW DC power at a tapping frequency of 90 BPM (Fig. 2F). Second, we optimize the FSCC for an optimized flyback regulation performance. Compared to our previous design (*10*), in which the FSCC is directly powered by the fluctuating *V*_1_, we add a high-efficiency buck converter to provide a stable and optimized FSCC supply voltage. Lowering this supply voltage will reduce the FSCC’s energy consumption (figs. S17 and S18) and improve cold-start speed. However, if the supply voltages are lower than the threshold voltage of *J*_B1_, the *J*_B1_’s on-resistance will substantially increase, resulting in substantial output degradation (Fig. 2G). Consequently, an optimum 2.5 V supply voltage is selected. In this case, the quiescent current of the SUCC and FSCC are 2.34 μA and 1.55 μA, respectively (fig. S17 and S18). Such low quiescent current levels ensure a fast cold start (figs. S19 and S20). Third, we optimize the values of *C*_1_ and *C*_2_ to balance the trade-off between start-up time and the duration for which the system can sustain operation after the biomechanical energy supply ceases (note S2, figs. S21 and S22). By implementing the three optimizations above, we can achieve a high DC output while also facilitating a fast cold-start process. A DC output power of ∼1.1 mW can be achieved (Fig. 2H, fig. S24), and the cold-start process can be as fast as 5.95 s (Fig. 2D).

### Design of the ULP motion sensor

The third component in our system is a ULP motion sensor. To make our system self-sustaining, the key design consideration is that the power consumption of our motion sensor system must be lower than the power supplied from the energy harvester and the PMC. We therefore focus on energy-aware system optimization, including low-power component selection and firmware-level power reduction, to enable continuous sensing and on-device edge-AI processing within the harvested power budget.

**Fig. 3.**
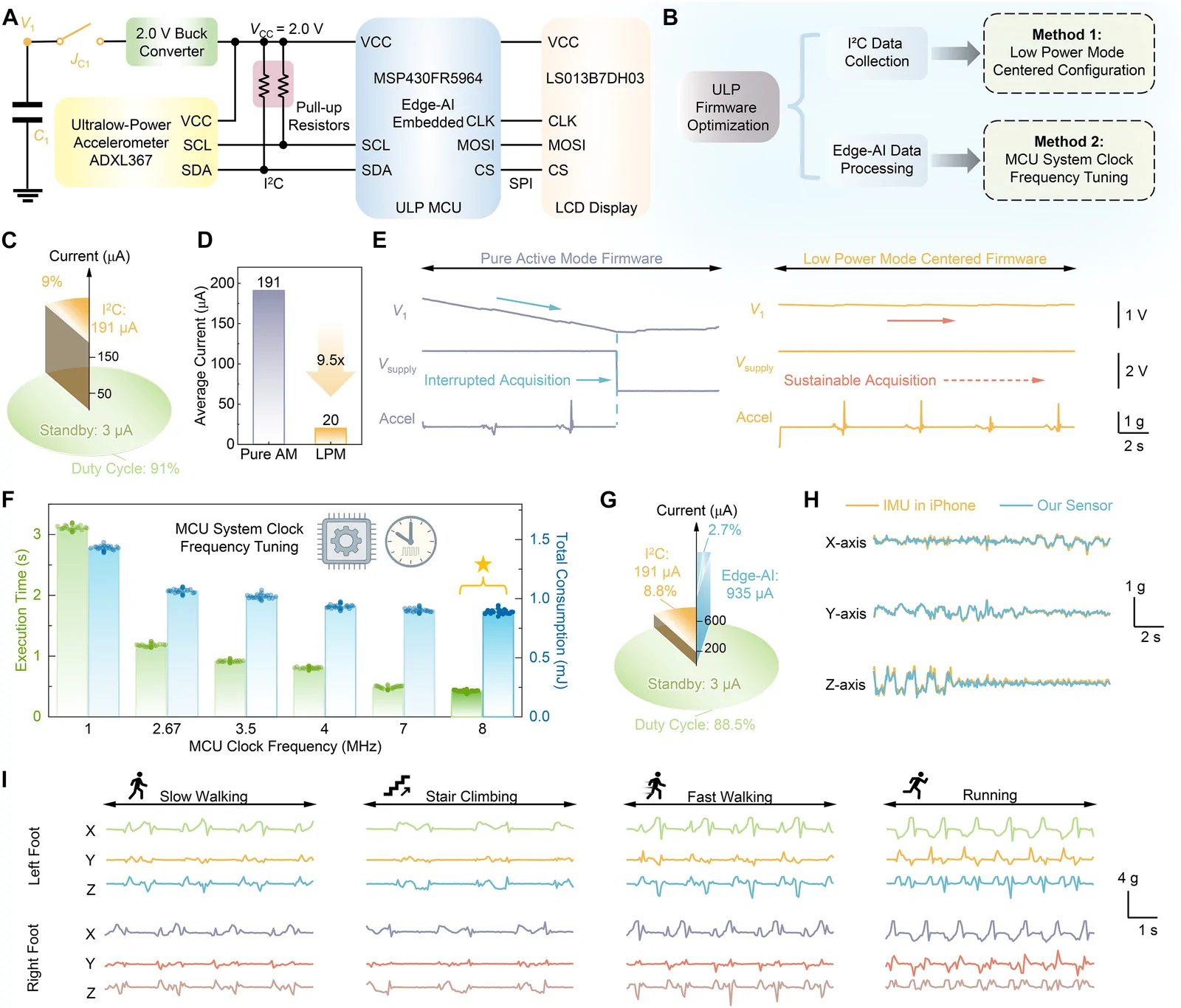
ULP motion sensor and firmware optimization for energy-efficient gait sensing. (**A**) Schematic and circuit diagram of the ULP motion sensor. (**B**) Stepwise, multi-pronged firmware optimization workflow to substantially reduce system-level energy consumption. (**C**) Stage-specific current consumption and duty cycle during the data-acquisition phase, illustrating the contribution of each stage to the overall energy budget. (**D**) Comparison of average current between a low-power-mode (LPM)–centered strategy (with standby) and a pure active-mode (AM; no standby) operation using firmware with only data collection functionality. (**E**) Motion-sensor operation powered by the energy harvester under pure AM and LPM-centered configurations (using firmware with only data collection functionality); a moving-window mean filter was applied to the raw signal to suppress noise and recover the underlying waveform. (**F**) Total system power consumption and total processing time during the data processing/display phase under different MCU clock frequencies. (**G**) Stage-specific average current consumption and duty cycle within a 15-second data-acquisition window using the final firmware, highlighting the energy contribution of each operating stage. (**H**) Measurement accuracy of the motion sensor compared with the built-in IMU of an iPhone (8 Hz low-pass filtered), evaluated using tri-axial acceleration for motion tracking in different directions. (**I**) Representative tri-axial acceleration signals from both feet under four motion scenarios: slow walking, fast walking, running, and stair climbing. For alignment of the two data streams from both feet, we performed a simultaneous jump at the beginning of the test and used the jump-induced acceleration peak signal as a mark to align.

Our ULP motion sensor comprises the following key components: a ULP MCU unit for edge-AI processing, a ULP three-axis microelectromechanical systems (MEMS) accelerometer for motion data acquisition, and a ULP display (Fig. 3A). An additional buck converter is used to provide an optimum 2.0 V power supply. To satisfy both the sensing performance and ultralow-power requirements, we first select all key components under strict power and function constraints. The accelerometer ADXL367 is chosen because it operates at a ULP consumption (only 0.89 μA, substantially lower than other accelerometers, table S2). Its accuracy is validated through comparative analysis with the built-in accelerometer of an iPhone under different motion conditions along various axes (Fig. 3H). For the MCU, we select MSP430FR5964 as the main MCU because of its large memory size and ultralow active-mode current (table S3). In addition, a 1.28-inch LCD (LS013B7DH03) is utilized as an ultralow-power local feedback interface, consuming only ∼12 μW in static display and ∼ 50 μW even under 1 Hz full-screen refresh. This local-display strategy is selected instead of continuous wireless transmission because radio communication would introduce a substantially higher energy burden and compromise the self-sustaining, battery-free design objective. Future versions could incorporate intermittent wireless transmission of compact algorithm-processed summaries, such as the final activity label, step count, and cumulative caloric estimate, rather than continuous raw accelerometer data.

To further minimize power consumption, on the hardware side, we optimize the inter-integrated circuit (I^2^C) serial communication pull-up resistor and supply voltage to reduce communication and conversion losses (fig. S25). On the firmware side, we implement a stepwise, multi-pronged firmware optimization to substantially reduce system-level energy consumption (Fig. 3B). Our final firmware consists of two major phases (fig. S26). The first is a data-acquisition phase, which performs I^2^C communication to sample the accelerometer data continuously (25 Hz sampling frequency) for a fixed time window (an additional firmware with only continuous data collection capabilities is also developed to collect datasets for the edge-AI algorithm training). The second is a data processing/displaying phase, which utilizes edge-AI to process the collected data and displays the insights on the LCD.

During the data processing/displaying phase, the MCU must remain in active mode (AM) to fulfill the complex edge-AI computation. However, during the data acquisition phase, substantial time intervals exist between I^2^C commands. Therefore, during data collection phase, we substantially reduce system power consumption by placing the MCU into low-power mode (LPM) (Fig. 3C). Through this optimization, the average current in the data acquisition phase decreases from 191 μA in pure AM to 20 μA, resulting in a 9.5-fold reduction (Fig. 3D, fig. S27). As a result, even a gentle walking motion at 12 BPM can support uninterrupted and sustainable gait data collection in this LPM-centered data-acquisition firmware (fig. S28), whereas in traditional pure-AM firmware, motion at 12 BPM only allows data acquisition for 13 seconds before power runs out (Fig. 3E, fig. S29).

During the data processing/displaying phase, we further reduce total MCU power consumption by increasing the MCU clock frequency. This is counterintuitive, since a higher MCU clock frequency typically means a higher supply current. However, increasing clock frequency shortens command execution time, and the net energy consumption during processing decreases (Fig. 3F, note S3). By increasing the clock frequency from 1 MHz to 8 MHz, we can reduce the total energy consumption of the processing/displaying phase by 37.6% and the total processing time by 86.4%. Therefore, we finally use an 8 MHz main clock during the data processing/displaying phase.

With these optimizations, our final firmware with a 15-second data acquisition window (window size selection see figs. S31 and S33) stays in an LPM idle state (3 μA supply current) for 88.7% duty cycle, stays in I^2^C communication state (190 μA supply current) for 8.8% duty cycle, and stays in edge-AI computation/display state (935 μA supply current) for 2.5% duty cycle (Fig. 3G). The overall average current is reduced from 191 μA to 43 μA (fig. S34), corresponding to 86 μW under the regulated 2.0 V supply. The complete system power consumption measured with the PMC stage is reduced from 377 μW to 108 μW (figs. S35 and S36). Even with edge-AI functionality, the power consumption of our sensor is reduced to approximately 2% of that of typical commercial accelerometers without edge-AI algorithms (mA-level, table S4).

By embedding the energy harvesters, the PMC, and the ULP motion sensors into a pair of shoes, we enable continuous, battery-free, long-term three-axis acceleration sensing, on-device edge-AI processing, and displaying the results. Due to our ULP design, even slow walking (∼22 BPM; fig. S37) can sustainably power the entire system. Our final wearable gait system supports prolonged and accurate gait sensing, local data processing, and result updating across diverse movement patterns without perturbing subjects’ natural gait. Importantly, raw accelerometer data do not need to be retrieved during the intended operation of the final system; instead, the collected acceleration signals are processed locally on the MCU, and only the algorithm-processed outputs are updated on the device. Raw accelerometer data were retrieved only during the dataset-building and AI algorithm-training stage, where offline data collection was necessary to develop and validate the activity-classification model. For this purpose, we collected 537 15-second gait data windows from four healthy volunteers across four locomotor modes: slow walking, fast walking, running, and stair climbing (Fig. 3I and table S5). The participants included three males and one female, with ages ranging from 23 to 26 years, heights ranging from 165 to 180 cm, and body weights ranging from 72 to 93 kg. The collected gait data show representative gait-cycle-related acceleration patterns under different locomotor modes. For example, in slow walking and stair-climbing trials, both left- and right-shoe sensing units captured acceleration patterns consistent with repeated stance and swing phases, qualitatively reflecting the alternating nature of bilateral gait.

### Design of the ULP edge-AI algorithm

**Fig. 4.**
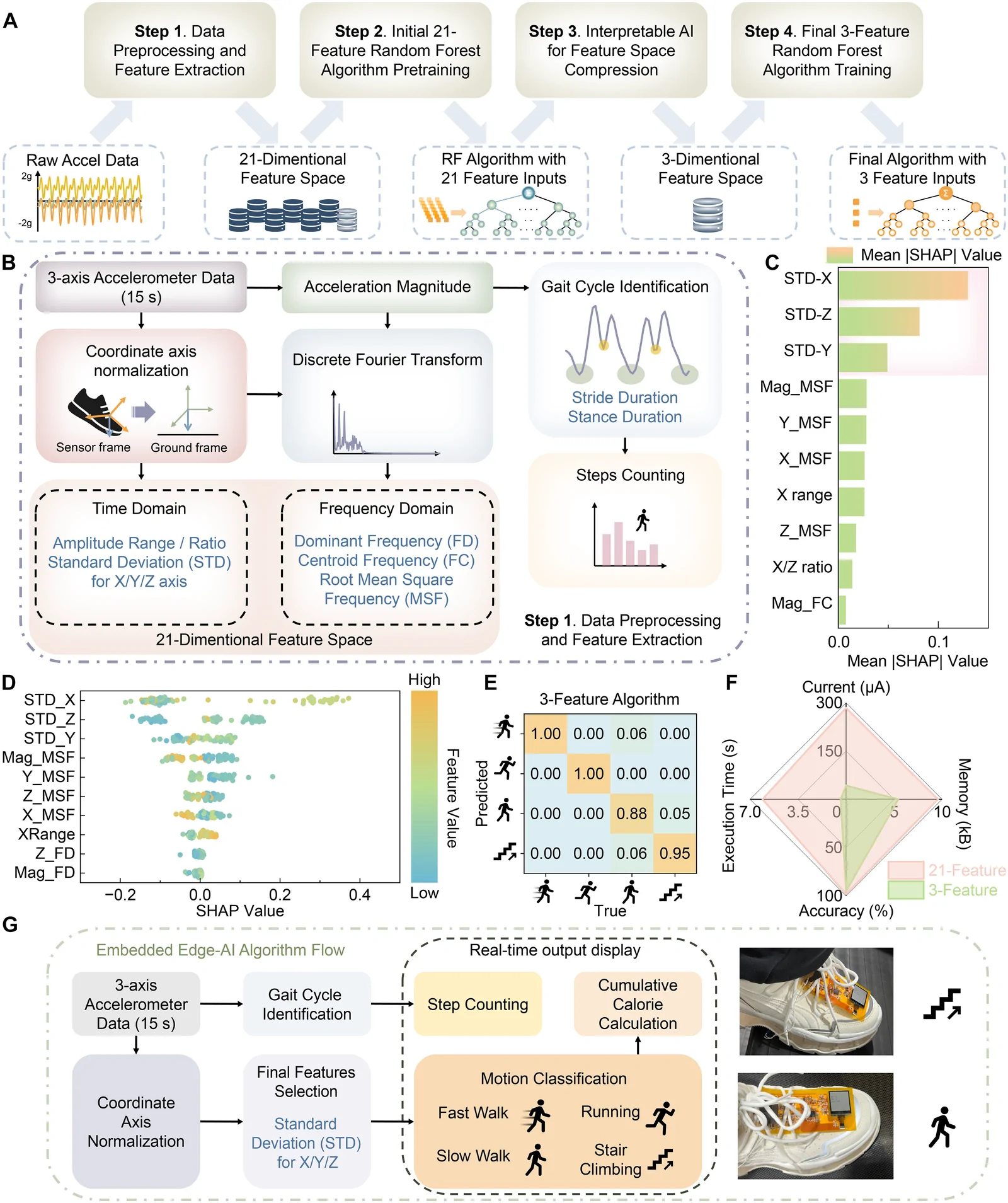
Development of an embedded edge-AI motion data-processing pipeline. (**A**) Optimization workflow of the edge-AI pipeline from offline model design to on-device deployment. (**B**) Detailed schematic of Step 1: accelerometer data preprocessing and feature extracion. (**C**) SHAP-based feature-importance ranking across 21 candidate features used to guide feature selection for embedded implementation (top 10 features shown). (**D**) SHAP beeswarm plot showing each feature’s contribution to model predictions, with samples predominantly assigned to Fast Walking class (top 10 features shown). (**E**) Confusion matrix of motion-type classification using the final time-domain–only model; frequency-domain features were excluded to avoid FFT-based spectral analysis and reduce computational burden. (**F**) Performance and efficiency comparison between the 3-feature and 21-feature models, including classification metrics, memory footprint, execution time per analysis window, and average current consumption, demonstrating reduced computational cost with minimal accuracy loss. (**G**) Final embedded algorithm flow integrating activity classification and step counting from accelerometer data with timely user feedback, together with representative photographs of stair-climbing and slow-walking scenarios illustrating correct motion recognition and accurate step counting on the embedded system.

We develop an edge-AI pipeline embedded on the MCU for motion activity classification, calorie calculation, and step counting, providing timely feedback to users (Fig. 4G). Deploying edge-AI on an ultralow-power MCU requires balancing strict power and memory constraints with the need for accurate and reliable classification, making algorithm optimization a key design focus. The edge-AI algorithm we choose is based on a random forest, where features are first extracted from raw signals, and the AI algorithm is then trained on these extracted features. Here, random forest is chosen because it combines low memory usage, high predictive performance, and inherent interpretability (*44*, *45*), thus avoiding the memory-intensive and power-hungry multiply–accumulate operations and matrix algebra. Through real-time on-device edge-AI processing, the step count, motion activity type, and calorie estimates [based on the Metabolic Equivalent of Task (MET) method (*46*, *47*)] are updated and displayed on the LCD, providing users with timely feedback on their activity status. In this context, “real-time” refers to on-device inference and timely result updating during system operation, in contrast to offline post-processing after data retrieval, rather than continuous visual checking of the shoe-mounted display during walking or running. In addition, the LCD serves as an ultralow-power local feedback interface for pause-and-check use.

The detailed edge-AI algorithm flow is as follows (Fig. 4A). First, for each 15-second segment of tri-axial accelerometer data, we preprocess the data and extract features (Fig. 4B). The raw signals are first transformed into a ground-referenced global coordinate system. The overall magnitude of the three-axis acceleration is then calculated. From the magnitude, we identify gait cycles and detect stride and stance phases (fig. S38). After preprocessing, statistical features are extracted for further edge-AI algorithm training. In the time domain, we extract features such as descriptors of motion amplitude [such as axis-wise magnitudes and standard deviations (STD)]. In the frequency domain, we perform a fast Fourier transform (FFT). Then we extract features such as the dominant frequency (FD), centroid frequency (FC), and mean square frequency (MSF), among others. This results in a final 21-dimensional feature space.

Second, we pretrain a random forest classifier using the 21-dimensional feature space. To avoid overfitting and ensure consistent performance across activity classes, we use cross-validated hyperparameter tuning. Our 21-feature-based model demonstrates excellent performance, achieving 98.1% accuracy with no signs of overfitting (figs. S41 to S43). However, its 9.573 kB memory requirement exceeds the maximum-allowed RAM size (8.192 kB), making this model non-deployable on our MCU. In addition, given that this model requires complex FFT pre-processing, it has a long execution time (6.1 s), which further leads to undesired current consumption (284.4 μA).

To make our pretrained, non-deployable classifier compact and suitable for deployment on the ultralow-power MCU, we use SHapley Additive exPlanations (SHAP), an interpretable machine-learning method that quantifies the contribution of each input feature to the model prediction (*44*, *48*–*50*), to evaluate feature importance and compress our feature space (Fig. 4, C and D, figs. S44 to S51). Through SHAP analysis to analyze global feature importance, which is quantified by the mean absolute SHAP value (|SHAP|) across all samples, we reveal that the standard deviations of the 3-axis accelerations are the top-3 dominant contributors to the decisions (Fig. 4C) (*51*). Based on this discovery, we therefore compress our 21-dimensional feature space to these three features and then eliminate all frequency-domain features. Correspondingly, we can also eliminate the FFT process in our data preprocessing step, which further reduces data processing time, power consumption, and memory requirements.

Fourth, we retrain a final lightweight random forest classifier, which preserves high classification accuracy (95.4%, Fig. 4E) while substantially reducing algorithmic complexity (Fig. 4F). Its memory requirement decreases from the original non-deployable 117% to 66%. The total execution time reduces by a factor of 15.2 (6.1 s to 0.4 s) per data window, and the average current consumption reduces by a factor of 6.6 (284.4 μA to 43.0 μA). This compressed random-forest edge-AI model matches the stringent computation, memory, and energy constraints of our platform. In addition to the simplified lightweight motion classifier, our system also captures motion duration and estimates caloric expenditure using a standard MET-based formulation (*46*, *47*). The final feedback suite includes motion classification, step counting, and caloric expenditure estimation (Fig. 4G). In system-level testing with the fully integrated prototype with edge-AI, these outputs are correctly updated and displayed on the LCD, demonstrating real-time on-device processing and local result updating. The shoe-mounted display allows the user to pause and check the updated results at any time, providing understandable feedback to the user (Fig. 4G).

## DISCUSSION

In summary, we present a biomimetic, ultralow-power, edge-AI-empowered, and self-sustaining gait-analysis platform that integrates biomechanical energy harvesting, cold-start power management, ultralow-power motion sensing, and MCU-deployable edge inference. This system-level co-design of energy generation, power regulation, sensing, and embedded intelligence enables on-device edge-AI analysis under a strict harvested-energy budget and supports self-sustaining continuous gait monitoring during locomotion-powered operation. With the tailored edge-AI algorithm, our ultralow-power motion sensor can intelligently extract accelerometer data, calculate steps, classify motions with 95.4% accuracy, and estimate calories, consuming only 86 μW. A fully integrated and high-output triboelectric transducer scavenges milliwatt-level energy from each gait cycle, while a dual-path PMC maximizes the harvested power, which exceeds the requirement from the sensor and the edge-AI algorithm. Our platform demonstrates that intelligence and energy autonomy can coexist within a single wearable platform, opening a transformative pathway toward the next generation of intelligent, always-on, and truly personalized digital health systems.

Building on this, future studies will expand the validation scope to larger and more diverse participant cohorts, including individuals with different ages, body weights, gait patterns, and health conditions. These expanded datasets will enable the embedded algorithm to move beyond basic activity classification, step counting, and caloric-expenditure estimation toward more physiology- and pathology-relevant outputs, such as fall-risk assessment, abnormal-gait-pattern recognition, disease rehabilitation-stage classification, mobility-decline detection, and long-term recovery-trend tracking. In addition to gait monitoring, the modular system framework may also be extended to other self-sustaining wearable and bioelectronic sensing applications by adapting the energy harvester, PMC, sensing module, and embedded algorithm. With task-specific algorithm training and validation, this framework may support future applications in cardiac monitoring, biochemical biomarker tracking, and long-term personalized digital health monitoring.

## MATERIALS AND METHODS

### Materials

Kapton and fluorinated ethylene propylene (FEP) were purchased from McMaster-Carr. Aluminum foil was purchased from Fisher Scientific. All semiconductor devices (diodes, transistors, and chips such as ADXL367, MSP430FR5964, etc.) and passive components were purchased from Mouser Electronics.

### Fabrication of the triboelectric transducer

The FPCB (142.71 by 7.99 cm) of the triboelectric transducer was fabricated by Shenzhen JDB Technology Co., Ltd., containing the polyimide substrate, metal patterns (using electroless nickel immersion gold (ENIG) surface finish), coverlays, and vias (fig. S3). Then, the FEP films (125 μm thickness) were directly attached using a double-sided Kapton adhesive tape. The FEP film adhered to the surface of every other metal pad, adhering to both the front and rear sides to minimize parasitic capacitance and preserve device performance. Besides this integrated FPCB-based triboelectric transducer, we also fabricated one using our previously reported method (*10*). In this method, a 125 μm-thick Kapton film (2.2 in. width) was patterned into a multilayer zigzag architecture comprising desired stacked layers. Aluminum foil and fluorinated ethylene propylene (FEP) sheets (125 μm thickness, sputtered with 200 nm copper using PVD75 RF Sputter)—each precisely cut to serve as the positive and negative triboelectric interfaces—were sequentially laminated onto the Kapton substrate using a double-sided tape.

### Evaluation of the triboelectric transducer

To quantify the *Q*_SC_, the triboelectric transducer electrodes were directly connected to a Keithley 6514 electrometer that was set to its charge measurement mode (fig. S5A). The device was mechanically actuated by hand tapping, motor tapping, or maximum-effort tapping, depending on the testing purpose: hand tapping was used as a benchtop test to mimic the repeated contact-separation process during normal walking, motor tapping was used to provide a controlled and repeatable mechanical input for long-term quantitative characterization, and maximum-effort tapping refers to the fastest repeated manual tapping achievable by the operator, which was used to approximate the upper excitation frequency during high-intensity human motion. Each tapping event produced a charge readout that was stored via LabVIEW through the unit’s IEEE-488 interface.

For *V*_OC_ characterization, direct voltage measurement using the electrometer (fig. S5B) would theoretically be preferred; however, the high-voltage output of the triboelectric transducer exceeded the upper voltage limit of the instrument’s measurement range. To circumvent this, an indirect current-measurement approach was employed (fig. S5C): the triboelectric transducer was connected to a 10-giga-ohm precision resistor, with one resistor terminal tied to one triboelectric transducer electrode and the opposite resistor terminal and corresponding triboelectric transducer electrode connected to the Keithley 6514, which was configured for current measurement. Under mechanical excitation, the generated current through the resistor was recorded via LabVIEW; *V*_OC_ was then obtained by multiplying the measured current by 10 giga-ohms. The same current-measurement approach was used to characterize output performance under different load resistances, with the 10 giga-ohms resistor replaced by resistors of other specified values.

### Fabrication of the cold-start PMC and the ULP gait sensing system

The flexible printed-circuit substrate was manufactured by PCBWay as a two-layer board using polyimide as the dielectric layer. Component placement and soldering were carried out via stencil-assisted reflow soldering in a controlled oven, ensuring reliable assembly and minimal thermal stress on the ULP circuitry.

### Validation of the gait sensing measurement results

To validate the accuracy of our ULP gait sensing circuit, we performed side-by-side comparisons with the iPhone’s built-in accelerometer under synchronized, randomized motion (result shown in Fig. 3H). We secured the ADXL367-based sensor board and an iPhone together using an elastic band and subjected them to randomized manual oscillations simulating gait dynamics. ADXL367 outputs were acquired via I^2^C by an MSP430FR5964 microcontroller, while the iPhone’s internal accelerometer data were captured in real time using the Phyphox app. Both data streams were time-aligned.

### Measurement of power consumption

For the ULP motion sensor module, average current consumption was measured using a Keithley DMM6500 digital multimeter configured in DC current mode. The multimeter was inserted in series with the positive terminal of a regulated 2 V supply (Fig. S30) and the module under test. During operation, the current signal was recorded, and, for each operating stage of interest (e.g., serial communication or edge-AI processing), the readings were averaged over multiple sampling intervals to obtain the stage-specific mean current and the corresponding stage duration. The overall average current of the module was then computed as a duty-cycle-weighted combination of these stage-specific mean currents. Power consumption of the ULP motion sensor module was calculated from the measured current and the corresponding supply voltage under each operating condition.

To characterize the power consumption of the complete system, a capacitor-discharge method was employed. A 1 mF storage capacitor (*C*_1_) was first charged to 5 V and then disconnected from the charging source, allowing it to discharge naturally while connected to the system under test and the input of a Keithley 6514, which was configured in voltage measurement mode. The capacitor voltage decay was recorded using LabVIEW, and the average power consumption was calculated by determining the decrease in stored energy from the voltage–time curve over a defined interval and dividing it by the corresponding time duration. Different from the power consumption measurement of the ULP motion sensor module mentioned above, this complete-system power consumption measurement included the PMC stage, storage capacitor *C*_1_, and the 2.0 V buck converter. As a result, the power consumption measured here contained additional quiescent current of the PMC circuit components such as FSCC and SUCC, the leakage current of the storage-capacitor *C*_1_, and the energy conversion loss from the 2.0 V buck converter.

### Testing the devices on human bodies

The Institutional Review Board office at Rutgers University approved the study protocol for this project (protocol number Pro2025000220). All participants provided informed consent before participation after the nature of the study was explained. Testing of the integrated system was conducted on four healthy volunteers across four locomotor modes: slow walking, fast walking, running, and stair climbing. Each participant first put on the shoes embedding the self-powered ULP gait system. Treadmill trials were conducted at 1.3 mph, 2.0 mph, and 3.0 mph to emulate slow walking, fast walking, and running, respectively, with each condition maintained for at least 3–5 minutes to ensure a steady-state gait. Stair-climbing was then evaluated on a stair climber set to 60 steps per minute (SPM). Accelerometer outputs from the triboelectric transducer-powered sensor were recorded and stored in the MCU’s FRAM. These data were retrieved post-exercise and utilized as a sample for subsequent analysis. For data segmentation and analysis, the continuous accelerometer recordings were divided into 15-second windows. The initial and final portions of each trial were excluded from the analysis to avoid potential non-steady-state effects associated with task initiation and termination.

### Random Forest–based activity classifier for ULP edge-AI deployment

Based on the raw accelerometer data, an initial dataset contained 21 statistically engineered features per sample, along with a categorical label. On the training set, a random forest (RF) classifier was tuned using a randomized hyperparameter search with 5-fold cross-validation. The search space covered typical RF hyperparameters such as the number of trees, maximum depth, minimum samples per split, and leaf size, etc. Multiple performance metrics (overall accuracy and macro-averaged F1-score) were monitored during cross-validation, and the model was refitted according to the best validation accuracy, yielding a locally optimal RF model for subsequent analysis. After preprocessing, the dataset contained 537 samples and was divided using an 80:20 stratified hold-out split. The stratified split produced 429 training samples and 108 test samples, with the test set containing 29 Fast Walking samples, 25 Running samples, 32 Slow Walking samples, and 22 Stair Climbing samples. Hyperparameter optimization was performed using 5-fold stratified cross-validation with shuffling enabled. A randomized search was first conducted using both accuracy and macro-averaged F1-score as evaluation metrics, with accuracy used for final refitting, followed by a local grid search around the best configuration. The final 3-feature RF used 20 trees, entropy-based splitting, a maximum depth of 24, max_features = “log2”, max_samples = 0.9633.

On top of this optimal RF model, SHAP was employed to interpret the 21-feature RF. A TreeSHAP Explainer was constructed for the chosen optimal RF model, and SHAP values were computed over the full feature space. Global feature importance was summarized as the mean absolute SHAP value across all samples, and features were ranked accordingly. The top 3 features, standard deviation of x-axis, y-axis & z-axis, were picked for further edge-AI implementation.

Next, a compressed dataset was then constructed by retaining only these three features and the corresponding labels. On this 3-feature dataset, the training/test split, cross-validation protocol, and hyperparameter tuning strategy were repeated. The goal in this phase was to strike a balance between predictive performance and model compactness, so the search space deliberately focused on relatively small ensembles and moderate tree depths. The final locally optimized 3-feature RF achieved competitive accuracy and macro-F1 on the test set, while substantially reducing the number of features, making it suitable for deployment on the MCU.

For embedded deployment, the final 3-feature RF classifier was exported from Python. The generated header file was then imported into the Code Composer Studio (CCS) project for the MSP430FR5964 and compiled together with the remaining firmware. At run time, the MCU continuously acquired tri-axial accelerometer data at a 25 Hz sampling frequency. After obtaining 15 seconds of tri-axial accelerometer data, the MCU computed the corresponding statistical features (standard deviations of the x-, y-, and z-axis), and arranged them into a three-element floating-point feature vector. This vector was passed to the function generated by Python, and the resulting class index is mapped back to the corresponding label using the same encoding employed during training, thereby enabling fully local, real-time classification directly on the MSP430FR5964.

RAM usage on the MSP430FR5964 was obtained directly from Code Composer Studio (CCS) using the post-build Memory Allocation report. After compiling the program, CCS provided a breakdown of RAM consumption by section, allowing the RAM footprint to be documented consistently across different implementations. Even when the build indicates that RAM capacity was exceeded, the combination of the reported failed allocation information and the defined RAM memory region still enabled the determination of the program’s effective RAM demand and identification of which allocations do not fit.

Execution time in this work was defined specifically as the end-to-end processing interval covering axis alignment, time-domain & frequency-domain feature extraction, and final edge-AI implementation. Timing was measured using CCS EnergyTrace, which provided time-resolved profiling for the targeted execution segment. For versions that include FFT, the baseline RAM usage exceeded the MSP430FR5964’s 8 KB SRAM, so the LCD buffer was relocated from RAM to FRAM to relieve RAM pressure. Because the LCD section was not used during the measured processing pipeline, this memory relocation does not affect the reported execution time and is treated purely as a memory-management adjustment.

### Gait cycle identification and step counting algorithm

The primary objective of the step-counting module is to quantify spatiotemporal gait characteristics, and the analysis began with gait cycle identification, which was performed by evaluating the total magnitude of the tri-axial acceleration to determine stride duration and stance duration. Based on characteristic acceleration patterns observed during the gait cycle, time intervals in which the acceleration magnitude remained close to the magnitude of gravity were identified as stance phases. A gait cycle was confirmed when the intermediate interval between two stance phases exhibited acceleration changes consistent with the expected foot-lift and swing pattern. Once all gait cycles within a given signal segment were identified, the number of cycles directly yielded the step count for that segment. The effectiveness of the step-counting algorithm was evaluated by independently comparing the steps detected from left- and right-foot recordings collected during the same trial intervals, demonstrating high side-to-side consistency in step-count estimation (table S6). These results also showed close agreement with manually counted steps, further confirming the accuracy of the algorithm.

### Caloric expenditure estimation

Caloric expenditure was estimated using a standard MET-based formulation (*46*, *47*)

Energy expenditure (kcal)=body weight (kg)×activity duration (h)×MET

In our implementation, body weight was either set to the subject-specific value or, when unavailable, to a default of 75 kg. The activity duration per estimate was fixed to the sampling window length (15 s, i.e., 15/3600 h). For each window, the edge-AI classifier assigned one of four locomotion modes (slow walking, fast walking, running, or stair climbing), and a corresponding MET value was selected for that activity (*46*, *47*) (table S7). The per-window caloric expenditure was then computed using the equation above and accumulated over time. The LCD display reported the cumulative caloric expenditure from device start-up, together with the current step count and recognized activity type.

## Supplementary Materials

### This PDF file includes:

Supplementary Text Figs. S1 to S51 Tables S1 to S7 References
