# Supplementary material for: A biomimetic, ultralow-power edge-AI-empowered and self-sustaining gait analysis system
Source: Sci Adv. 2026 Aug 19;12(34):eaeh9625. doi: 10.1126/sciadv.aeh9625 (PMC13488879; doi:10.1126/sciadv.aeh9625)
Supplement: Supplementary file 1 [file sciadv.aeh9625_sm.pdf]

Science Advances
AAAS

Supplementary Materials for

A biomimetic, ultralow-power edge-AI-empowered and self-sustaining gait analysis system

Fuying Dong *et al.*

Corresponding author: Simiao Niu, simiao.niu@rutgers.edu

Sci. Adv. **12**, eaeh9625 (2026)
DOI: 10.1126/sciadv.aeh9625

This PDF file includes:

Supplementary Text
Figs. S1 to S51
Tables S1 to S7
References

Supplementary Text

Note S1 | Triboelectric transducer COMSOL's simulation method

The attached-electrode contact-mode triboelectric transducer model selected here is a conductor-to-dielectric type of triboelectric transducer, in which the bottom metal acts as not only the triboelectric layer but also the bottom electrode. The surface charge density of the tribo-charges on the dielectric layer is defined as $-\sigma$ ($-7e\text{-}6\ C\cdot m^{-2}$). The total charge of the electrodes can be obtained by multiplying σ by the surface area (S) of the tribolayer. As is typical in experimental conditions, the entire structure is surrounded by air. The potential at infinity is chosen as the reference point for the electric potential, set to 0.

COMSOL's simulation of the transducer is divided into two stages: the initial stage, when the upper and lower electrodes are in full contact, and the end stage, when the upper and lower electrodes are completely separated from each other. The dielectric material is FEP. The electrodes are separated by Kapton (Fig. S2a). For each stage, the parameters utilized are shown below:

Parameters used in the Triboelectric transducer simulations	Values
Length	100 [mm]
Width	50 [mm]
Surface charge density σ	7e-6 [$C\cdot m^{-2}$]
Electrode thickness	2e-2 [mm]
FEP thickness	0.125 [mm]
Kapton thickness	0.1 [mm]

The simulation outputs are provided in Fig. S2b.

Note S2 | Optimization of the value of C_1 and C_2

The primary objective here is to balance the trade-off between start-up time and the duration for which the system can sustain operation after the biomechanical energy supply ceases. When C_1 increases from 100 μF to 1 mF, the duration for which the system can continue operating after energy input ceases extends from 3.9 s to 38.5 s (Fig. S21). However, this increase in energy storage capacity also results in a longer cold-start time (Fig. S22). Based on the requirement to operate our motion sensor, the C_1 is chosen as 1 mF. Similarly, a smaller C_2 makes C_2 easier to drain out in Stage 2, but less time is needed to charge C_2 in Stage 1. As a result, the number of charging cycles required to alternately charge C_2 and C_1 increases (Fig. S23a-c). However, if C_2 becomes too small, each activation of J_{A1} results in a rapid voltage drop of V_2 to the threshold V_{thf2}, preventing sufficient time to transfer energy to C_1 and ultimately hindering successful cold start (Fig. S23d).

Note S3 | Clock-frequency scaling for minimizing energy in the processing/displaying phase

The MSP430FR5964 datasheet indicates that, in active mode with no wait states, the MCU supply current increases approximately linearly with the main clock frequency. This behavior can be modeled as

$$I_{AM}(f) = \alpha f + I_0$$

where f is the main clock frequency (MHz), α is the slope (μA/MHz), and I_0 is the frequency-independent offset current (μA). As an example, a representative curve reported in the datasheet can be expressed as

$$I_{AM,\ 75\%}(f)[\mu A] = 118 \cdot f[MHz] + 74$$

During the processing/displaying phase, the computational workload is approximately fixed for each decision/update (i.e., a fixed number of clock cycles). Therefore, the processing time can be modeled as inversely proportional to clock frequency:

$$t(f) = \frac{N}{f}$$

where N denotes the effective number of clock cycles required for one processing/displaying operation.

The total energy consumption of the processing/displaying phase can be approximated by

$$E(f) = V\int I(t)\,dt = V \cdot\ I_{AM}(f) \cdot t(f)$$

Substituting the two relationships above yields

$$E(f) = V(\alpha f + I_0)\frac{N}{f} = VN\left(\alpha + \frac{I_0}{f}\right)$$

This equation explains why increasing f can reduce energy even though the instantaneous current increases: the execution time decreases approximately as $\frac{1}{f}$, and the frequency-independent term I_0 contributes for a shorter duration, thereby reducing its overall energy cost.

Note S4 | Gait steadiness analysis based on raw acceleration data

In addition to the real-time embedded processing and edge-AI–based activity classification performed on single-foot accelerometer data, we conducted an offline gait steadiness analysis using bilateral raw acceleration signals to enable a more detailed characterization of gait. For gait steadiness analysis, the focus is on quantifying the quality and regularity of locomotion. As illustrated in fig. S39, the analysis begins with preprocessing. Taking advantage of the flexibility of offline processing, we not only perform coordinate-axis alignment to transform sensor-frame accelerations into the ground-referenced frame, but also apply a 3 Hz low-pass filter to reduce high-frequency noise. After preprocessing, gait cycles are identified using the same acceleration-magnitude-based method described in the Methods section, and subsequent steadiness metrics are computed on a cycle-by-cycle basis. Within each identified gait cycle, a set of clinically relevant smoothness metrics is extracted to evaluate gait steadiness (*52*) (fig. S39). These include harmonic ratios (HR) and root mean square (RMS), which measure the rhythmic consistency of acceleration patterns during locomotion. Additionally, normalized jerk score and log-dimensionless jerk (LDLJ-A) quantify rapid directional fluctuations and abrupt neuromuscular adjustments. In addition to these smoothness indices, symmetry quantification is conducted based on temporal gait parameters, including mean stride duration, mean stance duration, and the stance-to-stride ratio. To evaluate inter-limb symmetry, the Ratio Index (RI) and Robinson Index (SI) are computed (*53*), where RI values approaching 1 and SI values nearing 0 indicate greater symmetry between the left and right limbs during gait. Experimental results from healthy participants corroborate the effectiveness of these metrics, demonstrating consistency with established gait analysis paradigms (fig. S40).

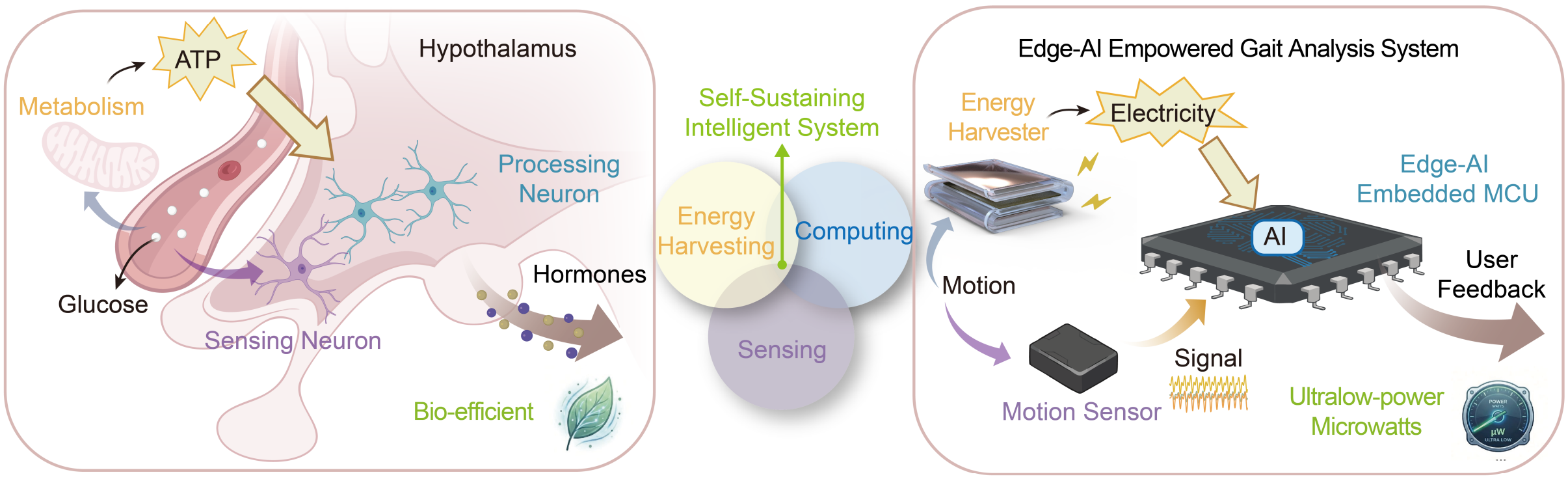

Fig. S1. Conceptual schematic of the bio-inspired edge-AI-empowered gait analysis system. The hypothalamus harvests blood glucose as an energy source through metabolic processes, incorporates multiple physiological sensors (e.g., glucose-sensing neurons), and local neural circuits perform ultra-efficient, low-power inference to generate downstream outputs. Analogously, our system integrates energy harvesting, motion sensing, and on-device edge-AI computation on an embedded MCU to enable ultralow-power gait-state inference and provide timely user feedback.

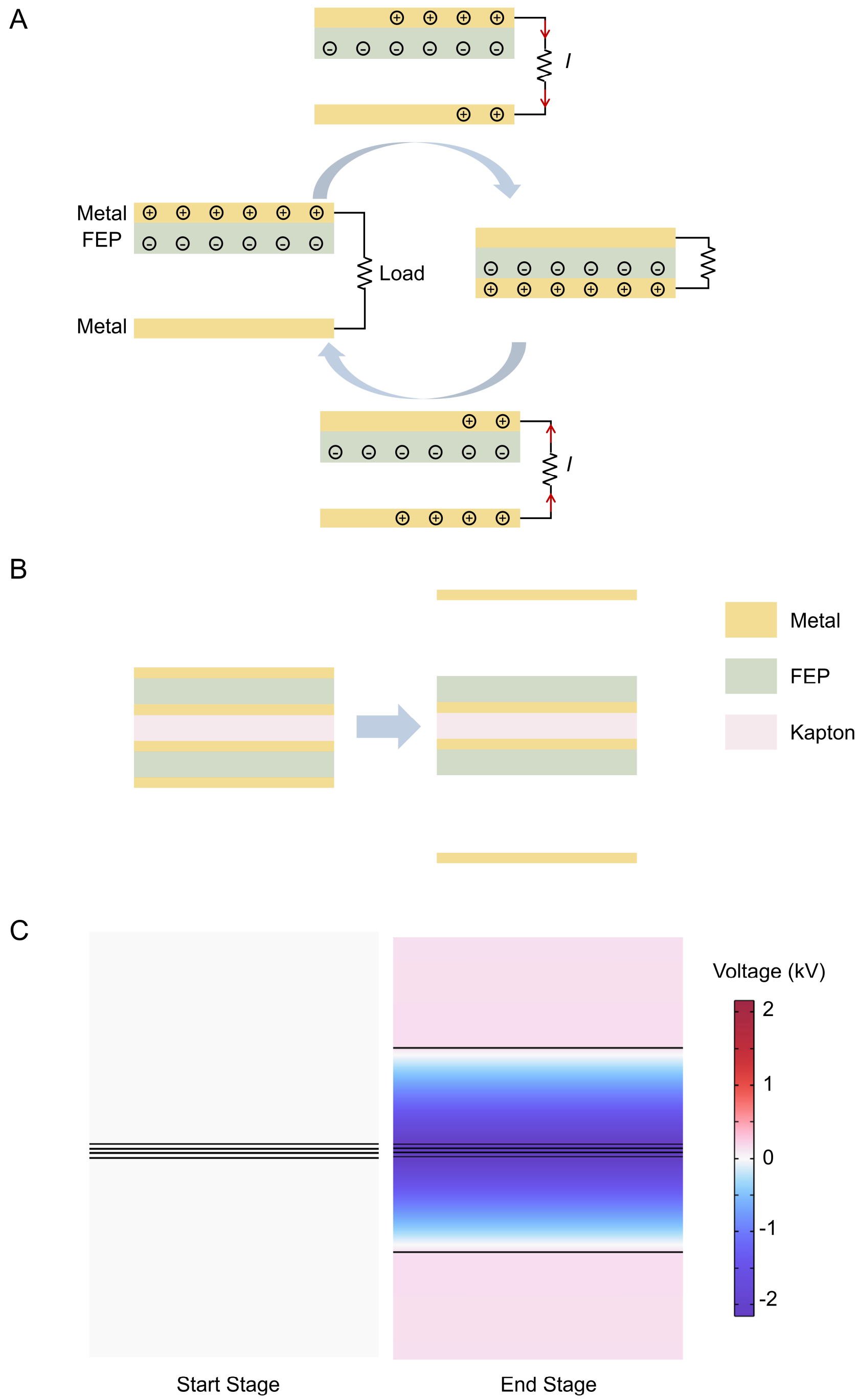

Fig. S2. Working mechanism and COMSOL's simulation of the attached-electrode contact-mode triboelectric transducer. (A) Schematic illustration showing the periodic contact–separation process and charge transfer between triboelectric layers. (B) Simulation model illustrating the initial and finite stages. (C) Simulated electric field distribution at the start and end of the contact–separation cycle.

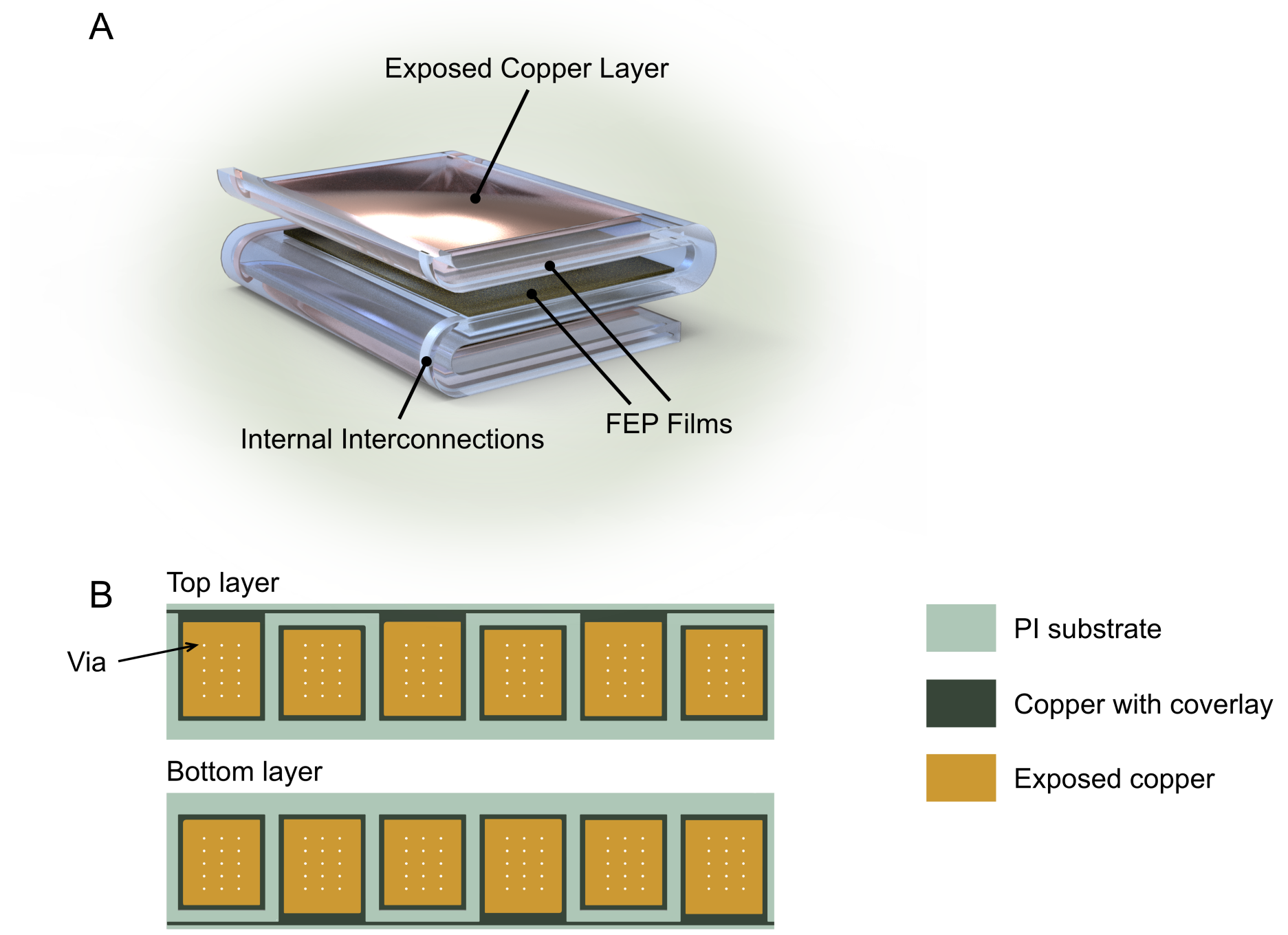

Fig. S3. Structural details of the two-layer flexible printed circuit board-based triboelectric transducer. (A) Folded 3D view highlighting the assembled layer stack and overall geometry. (B) Partially enlarged view with annotated design features, emphasizing key structural elements.

A

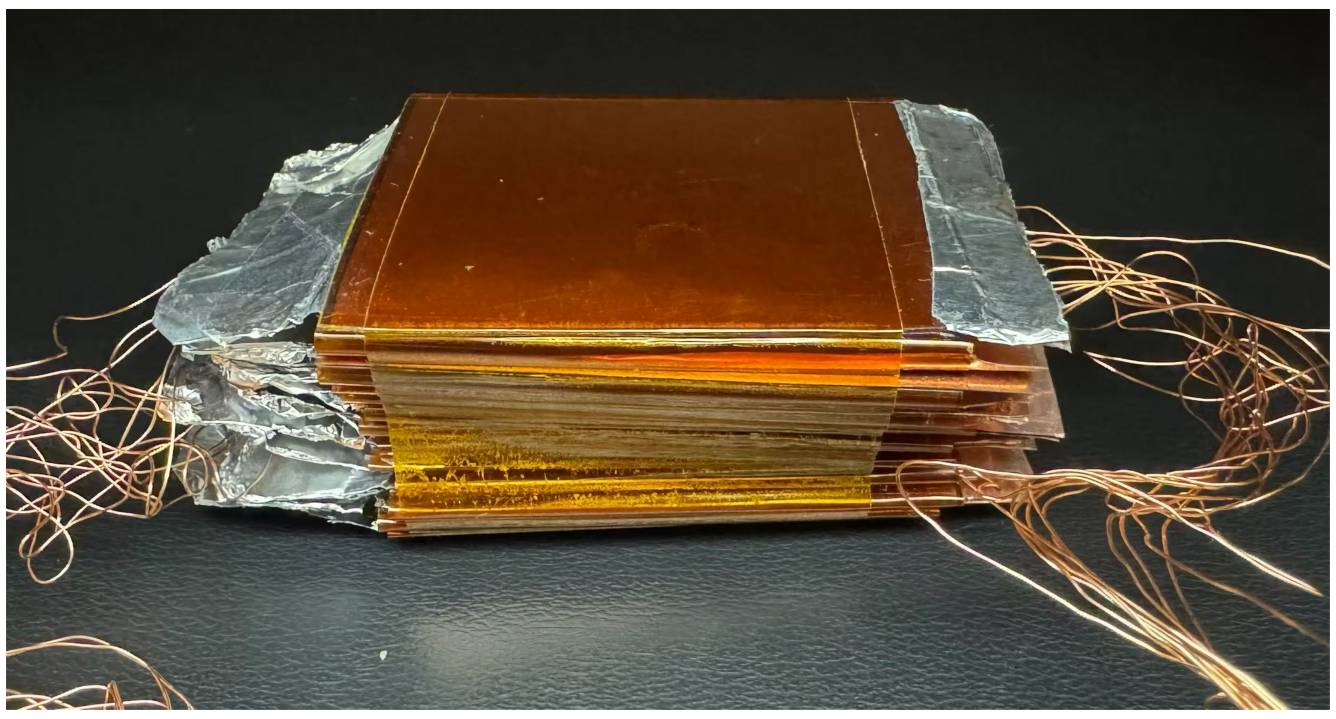

B

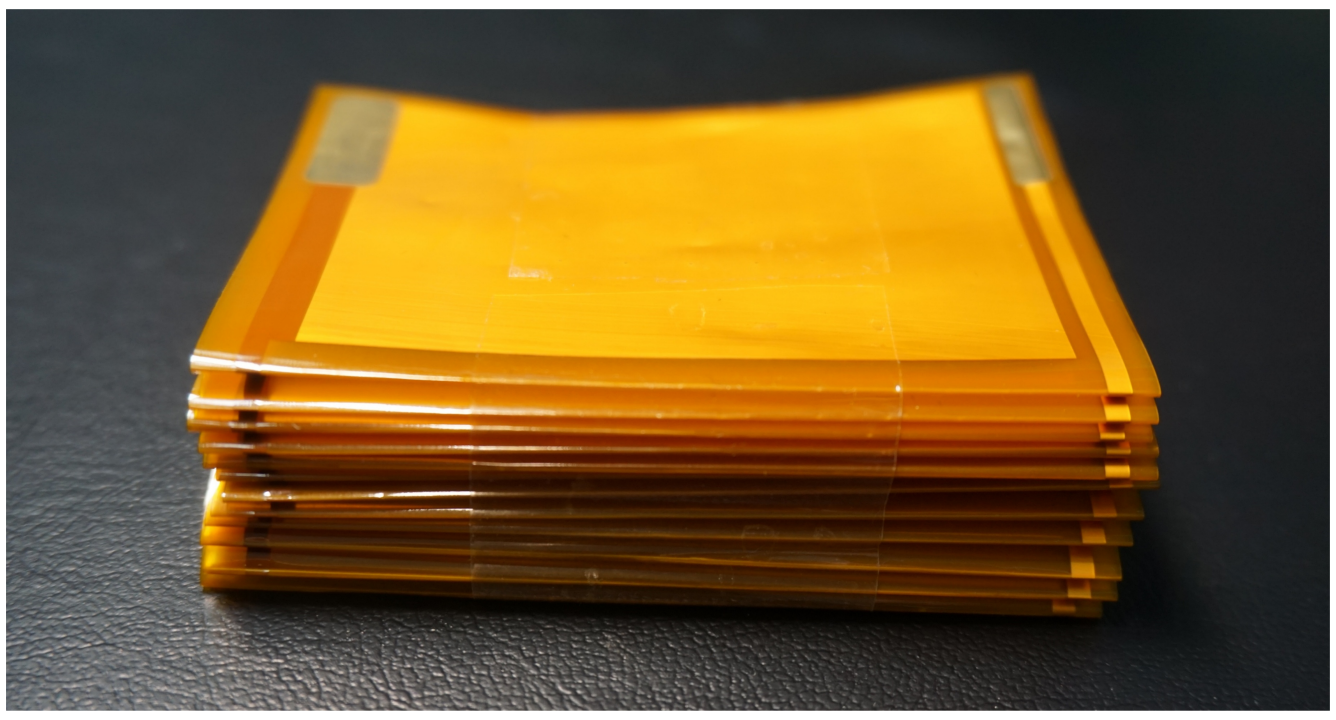

Fig. S4. Comparison of two multilayer triboelectric transducer designs. (A) Conventional design requiring external wire connections. (B) Compact design using flexible printed circuit board fabrication.

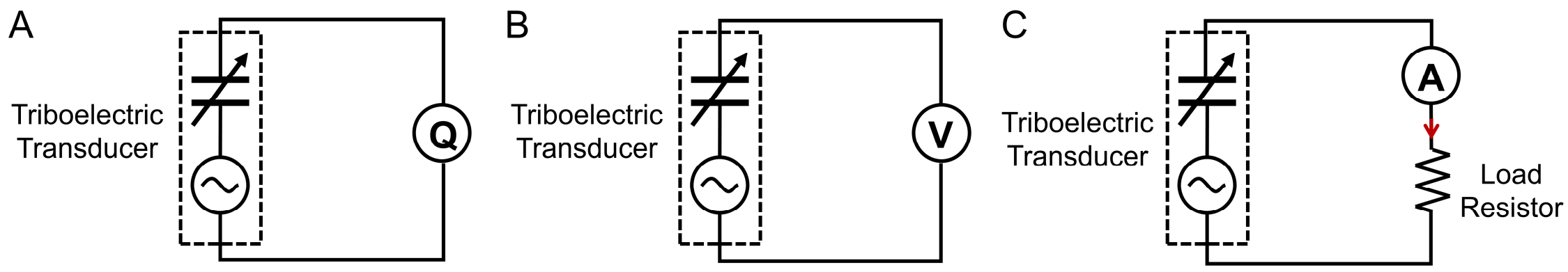

Fig. S5. Test circuits for triboelectric transducer output characterization. (A) Circuit for short-circuit transferred charge (Q_{SC}) measurement. (B) Direct-measurement circuit for open-circuit voltage (V_{OC}). (C) Circuit for current output measurement.

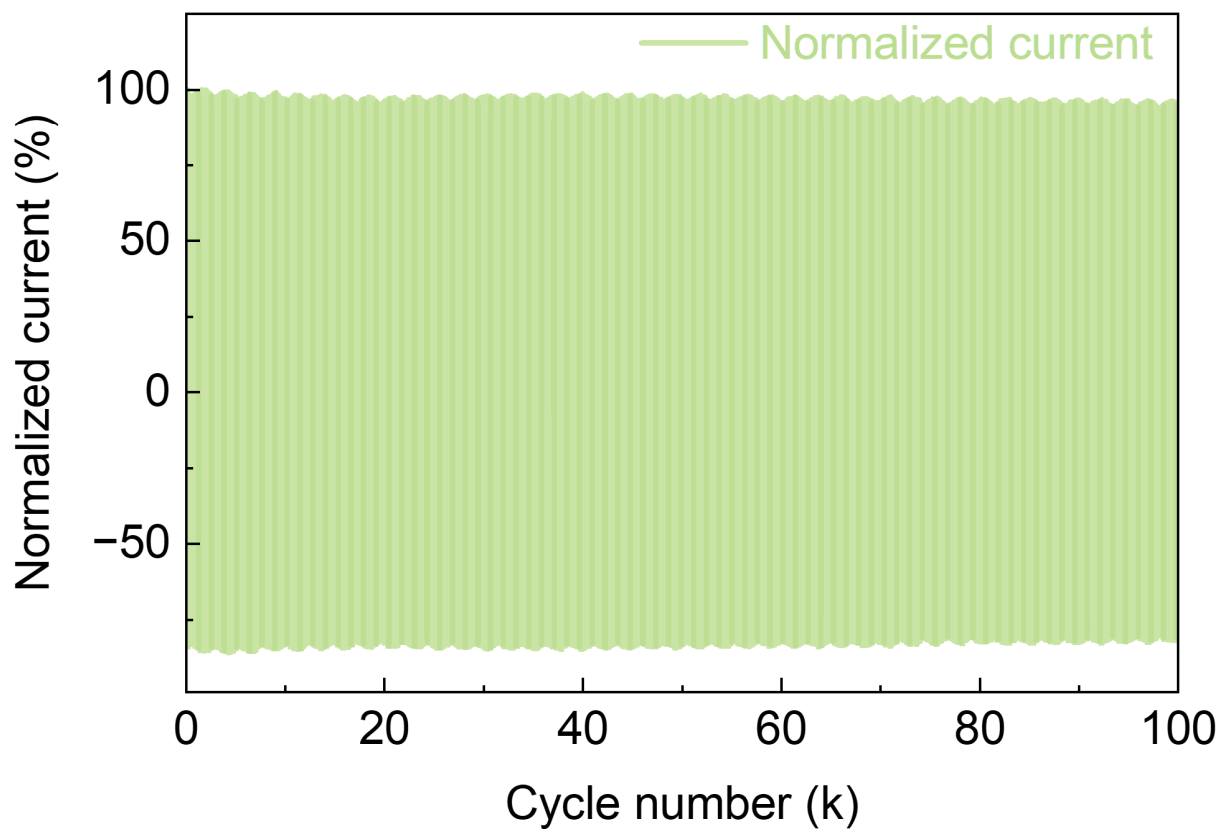

Fig. S6. Durability of the triboelectric transducer. Output performance after 100,000 compression–rebound cycles shows negligible degradation.

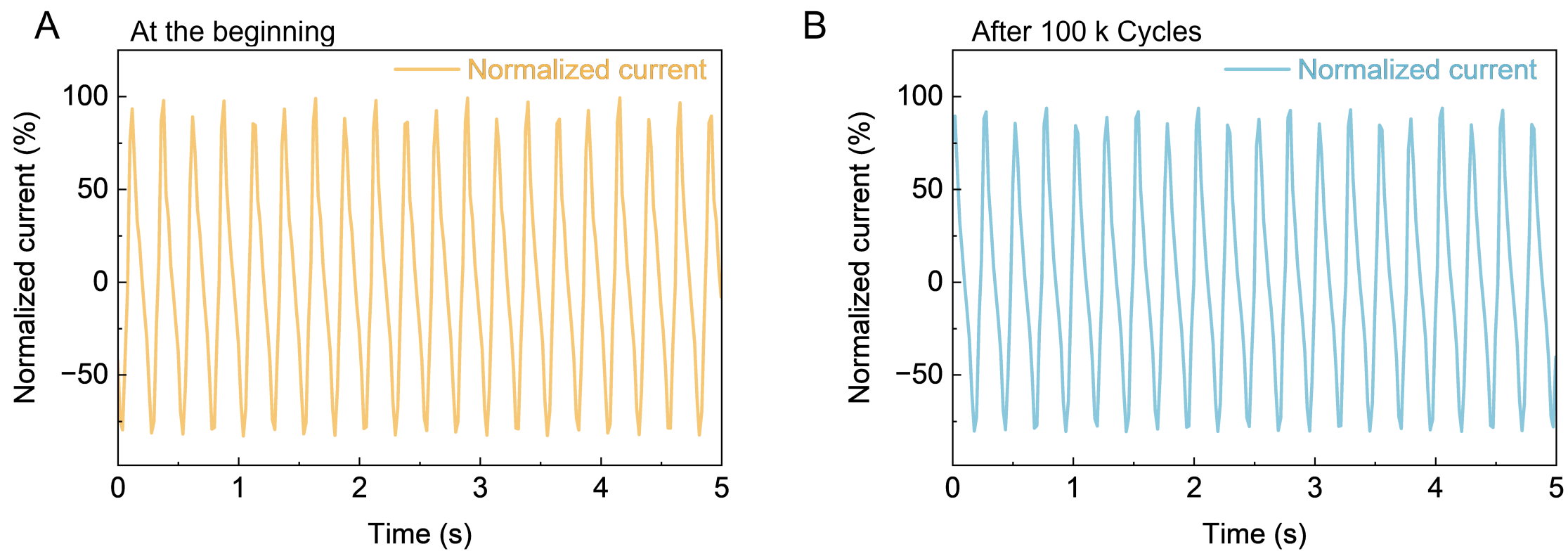

Fig. S7. Output signal before and after durability testing. (A) Initial output signal. (B) Output signal after 100,000 compression–rebound cycles.

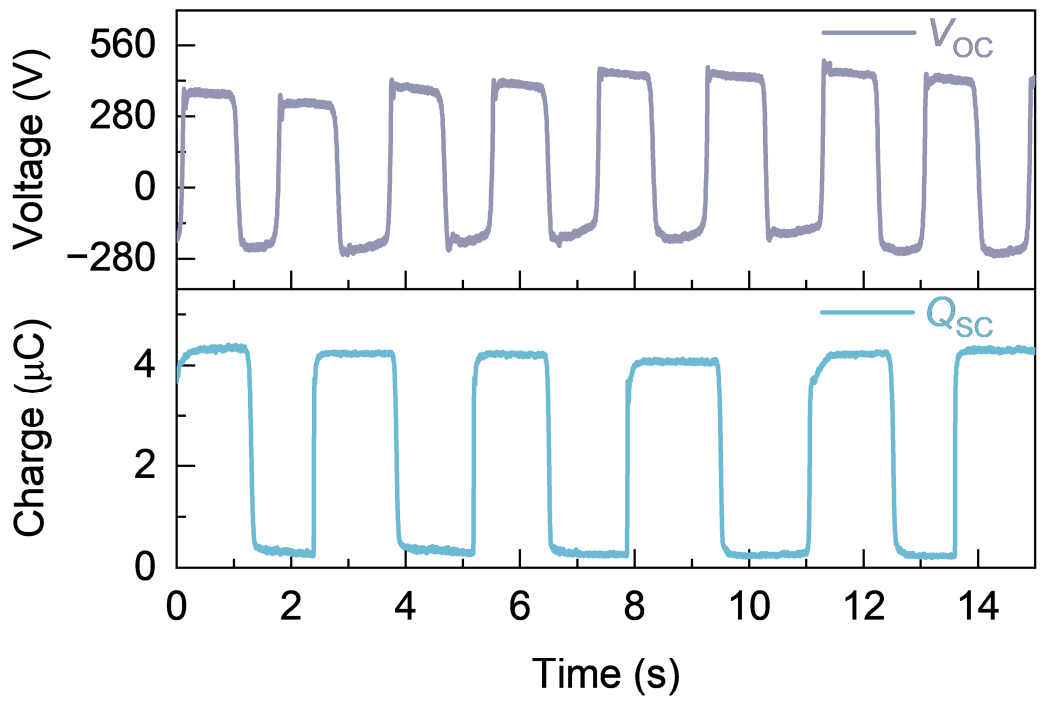

Fig. S8. Output performance of the conventional multilayer triboelectric transducer. Short-circuit transferred charge (Q_{SC}) and open-circuit voltage (V_{OC}) measured under cyclic loading. A 5 Hz low-pass FIR filter is applied to remove power line noise.

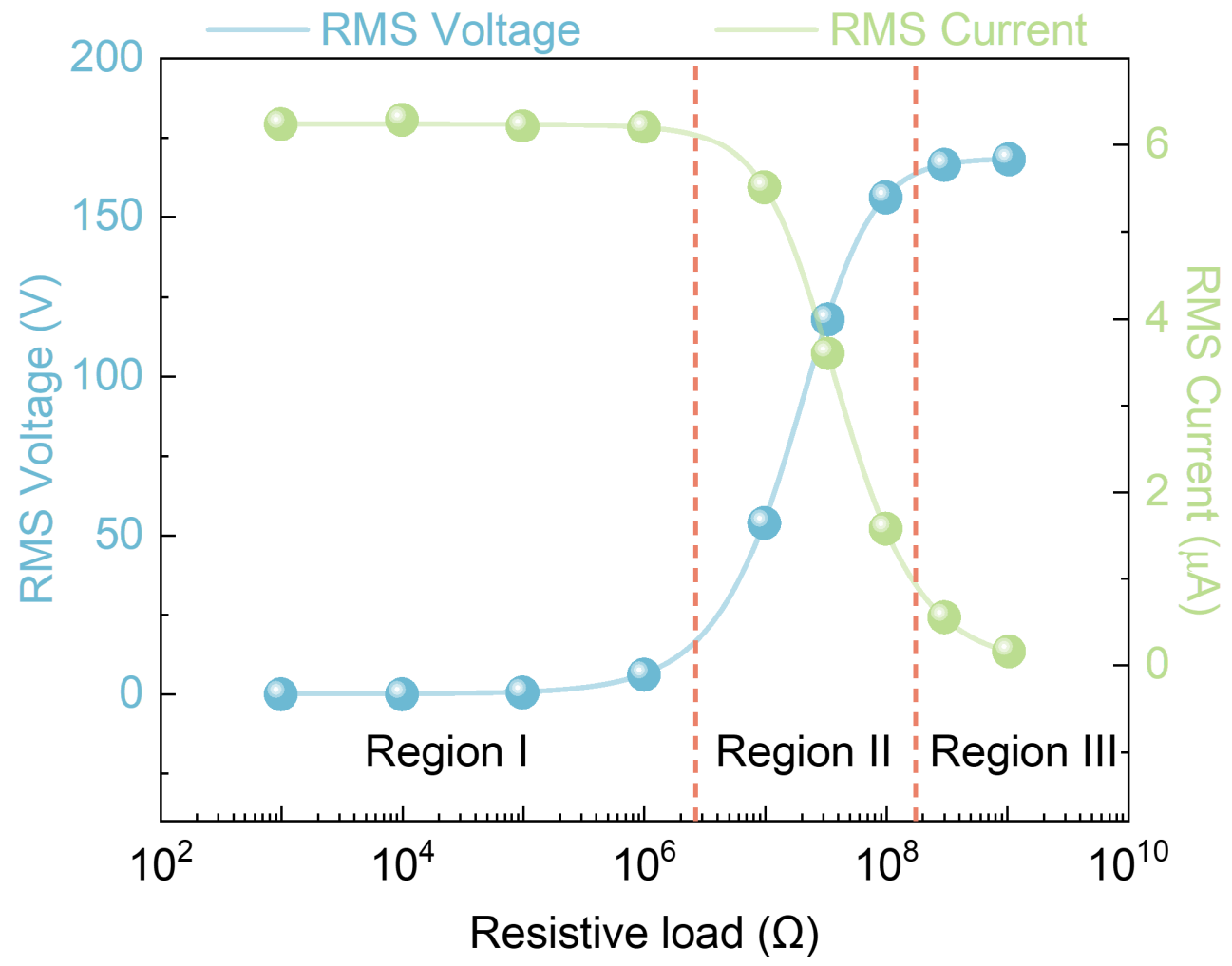

Fig. S9. Effect of load resistance on output current and voltage. Experimental results (dots) and interpolated curves showing three distinct working regions.

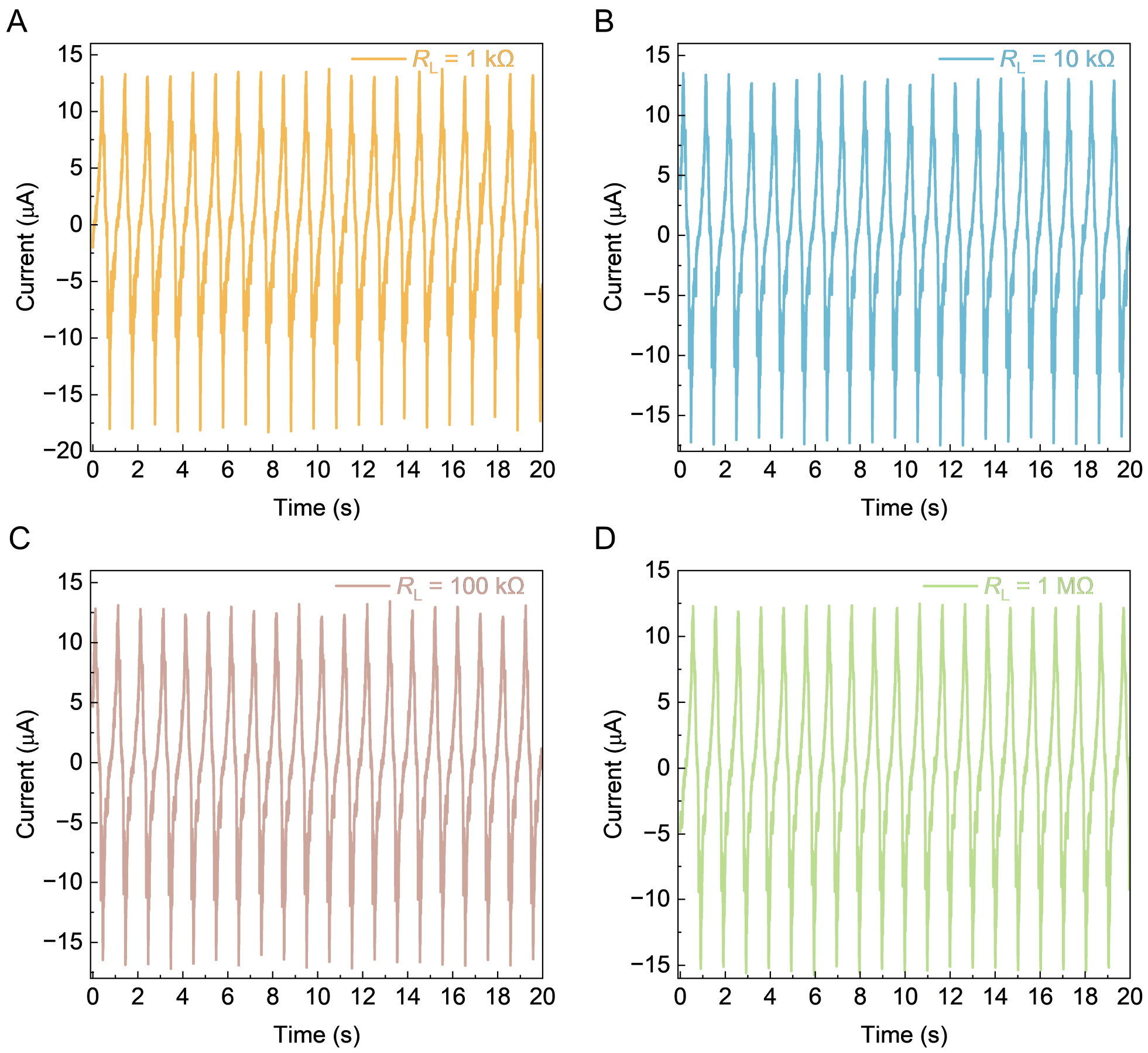

Fig. S10. Output current in working region I. (A) Load resistance = 1 kilohm. (B) 10 kilohms. (C) 100 kilohms. (D) 1 megaohms.

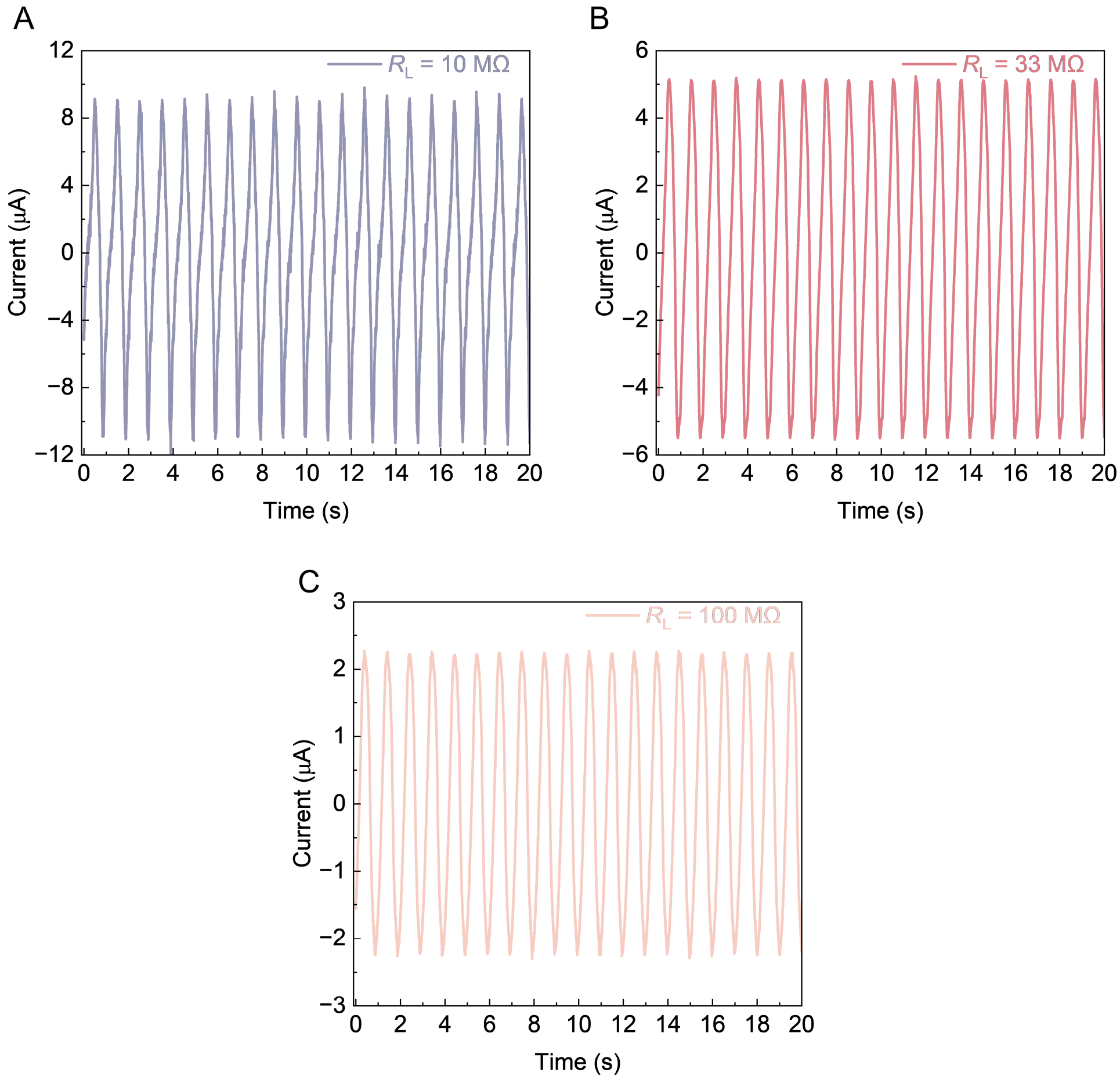

Fig. S11. Output current in working region II. (A) Load resistance = 10 megaohms. (B) 33 megaohms. (C) 100 megaohms.

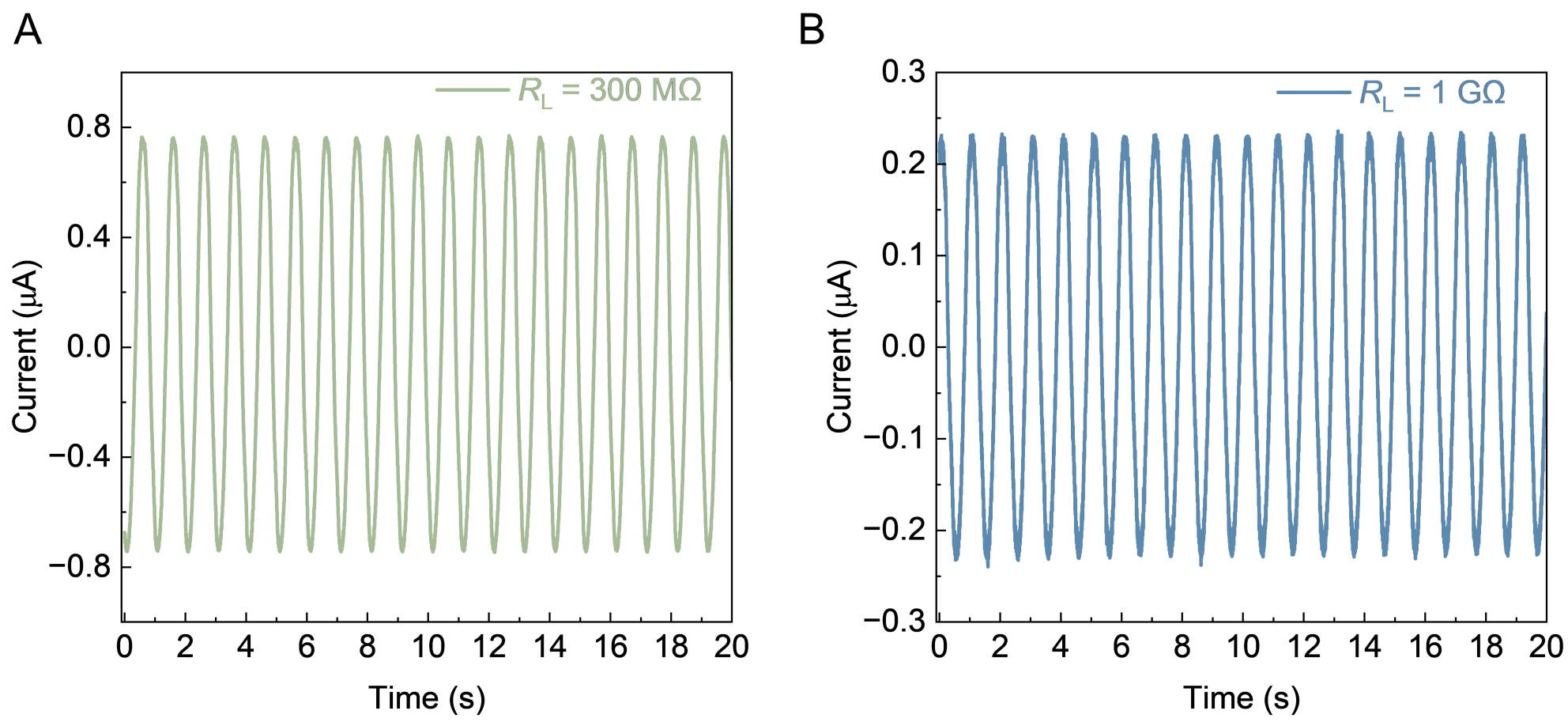

Fig. S12. Output current in working region III. (A) Load resistance = 300 megaohms. (B) 1 giga-ohm.

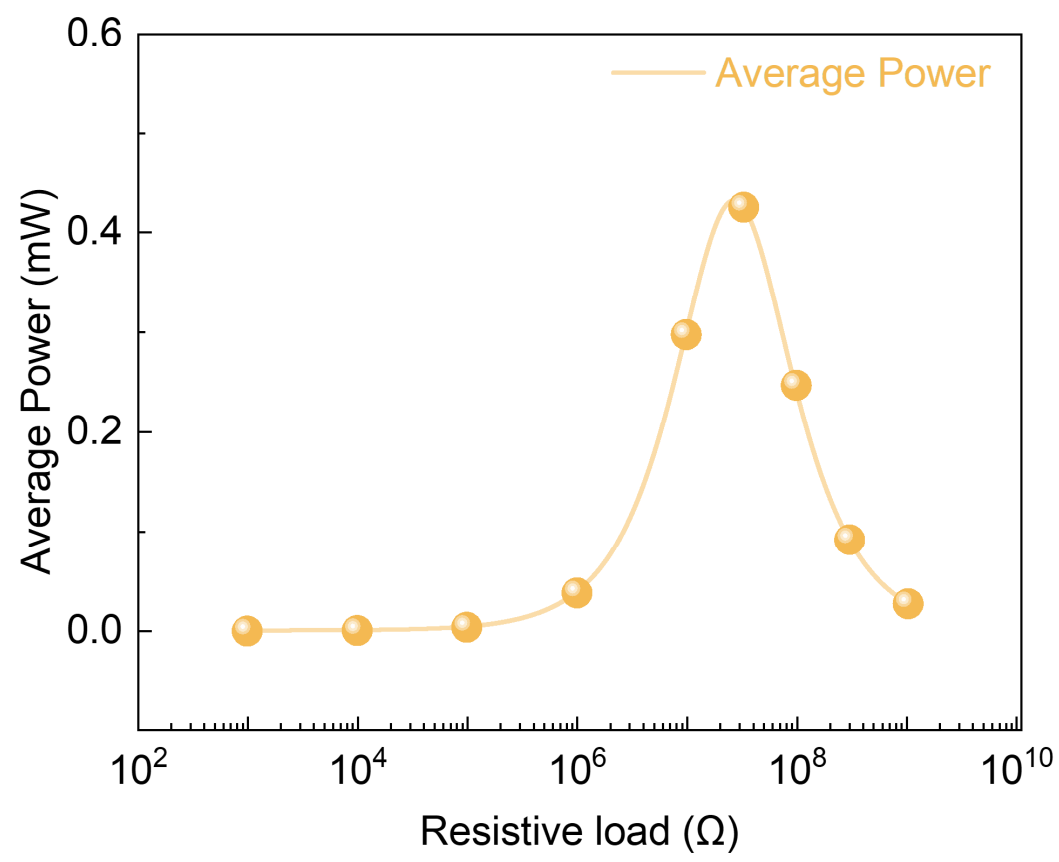

Fig. S13. Maximum output power versus load resistance. Experimental data (dots) and interpolated curves showing the variation of maximum output power across resistive loads.

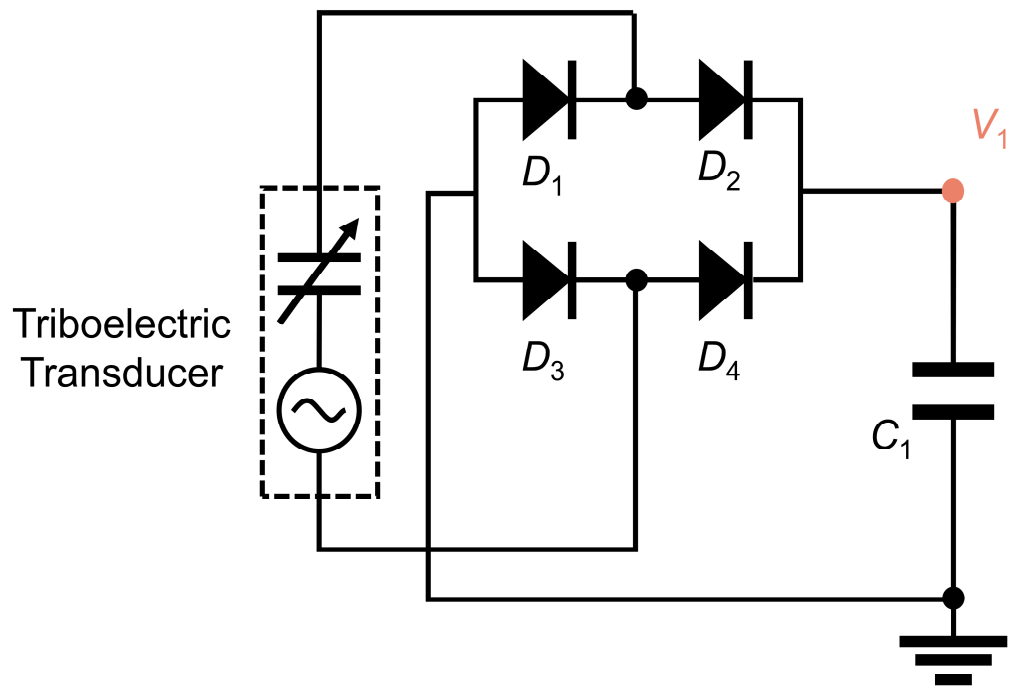

Fig. S14. Conventional diode-bridge rectification configuration. Circuit diagram of the standard diode-bridge arrangement used for AC energy harvesting.

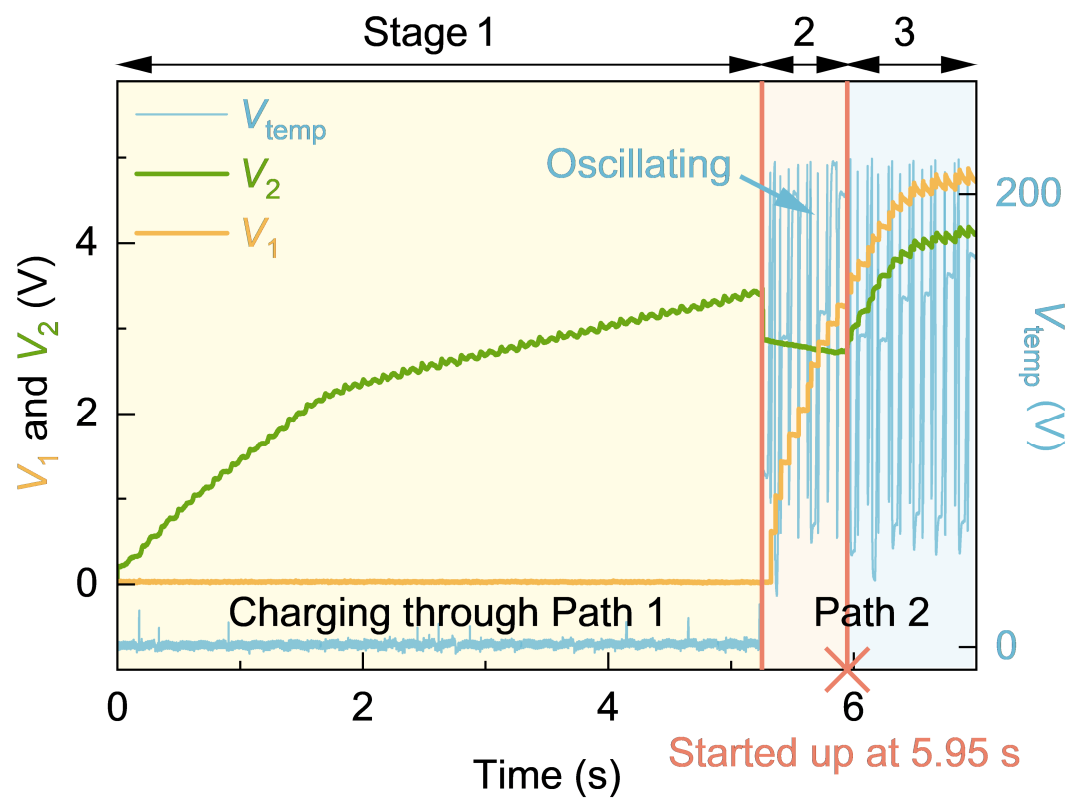

Fig. S15. Complete "dual-path" cold-starting process, including working stages (1–3), illustrating circuit functionality. Experiments were conducted with C_1 = 100 μF and C_2 = 22 μF during a maximum-effort-tapping process, and data were recorded by an oscilloscope; a moving-window average filter was applied to the raw signal to reduce noise and recover the true waveform.

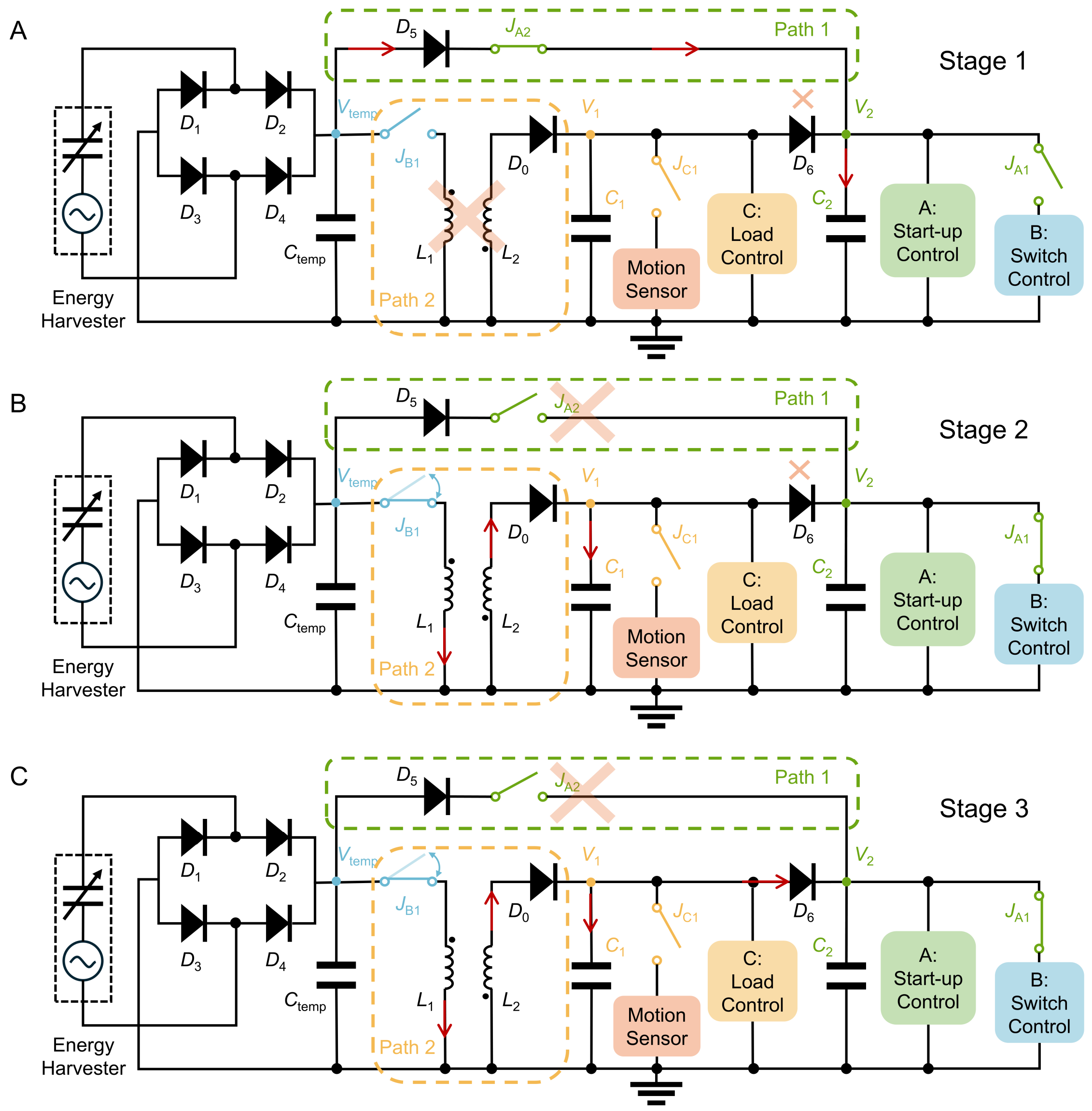

Fig. S16. Annotated energy-flow paths in the cold-start PMC. (A) In Stage 1, the harvested energy flows through Path 1 and passively charges the start-up capacitor C_2 through the closed depletion-mode switch J_{A2}. (B) In Stage 2, after C_2 reaches the threshold voltage, the circuit switches to Path 2, where the flyback converter transfers the harvested energy to C_1. (C) In Stage 3, C_1 charges C_2 through D_6, completing the cold-start process and allowing the PMC to remain in the optimized flyback-conversion configuration.

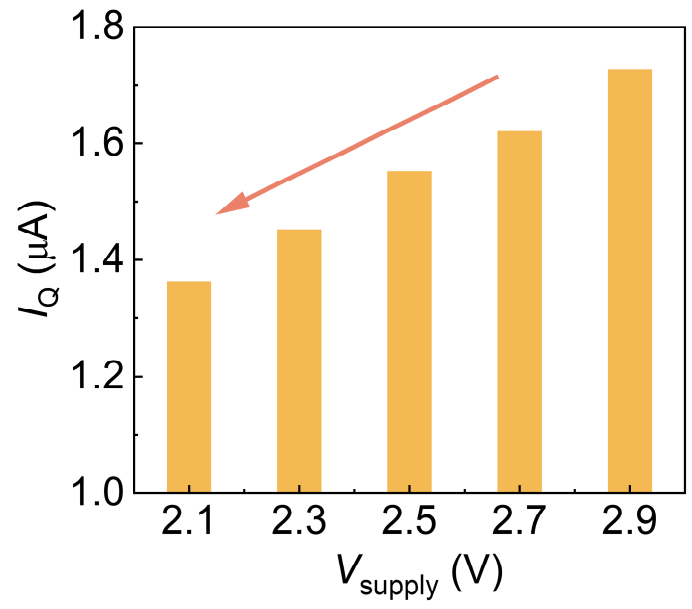

Fig. S17. Quiescent current of the flyback switch control circuit (FSCC). Measured quiescent current values at different supply voltage levels.

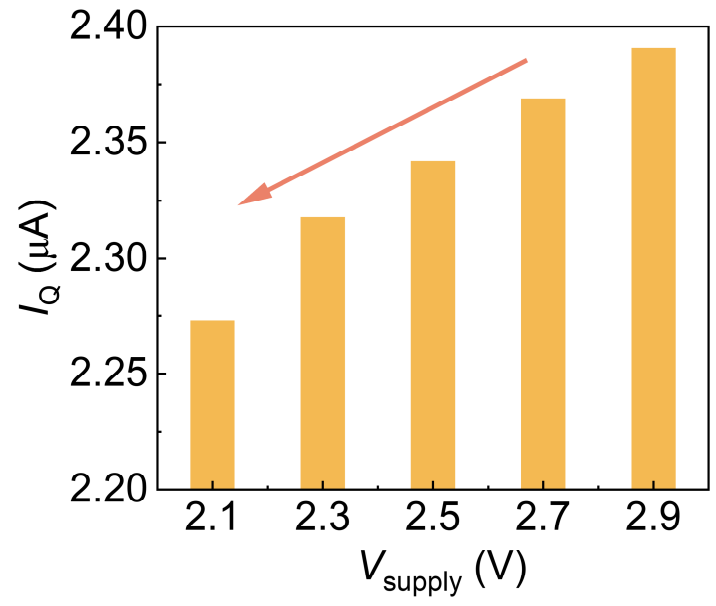

Fig. S18. Quiescent current of the start-up control circuit (SUCC). Measured quiescent current values at different supply voltage levels.

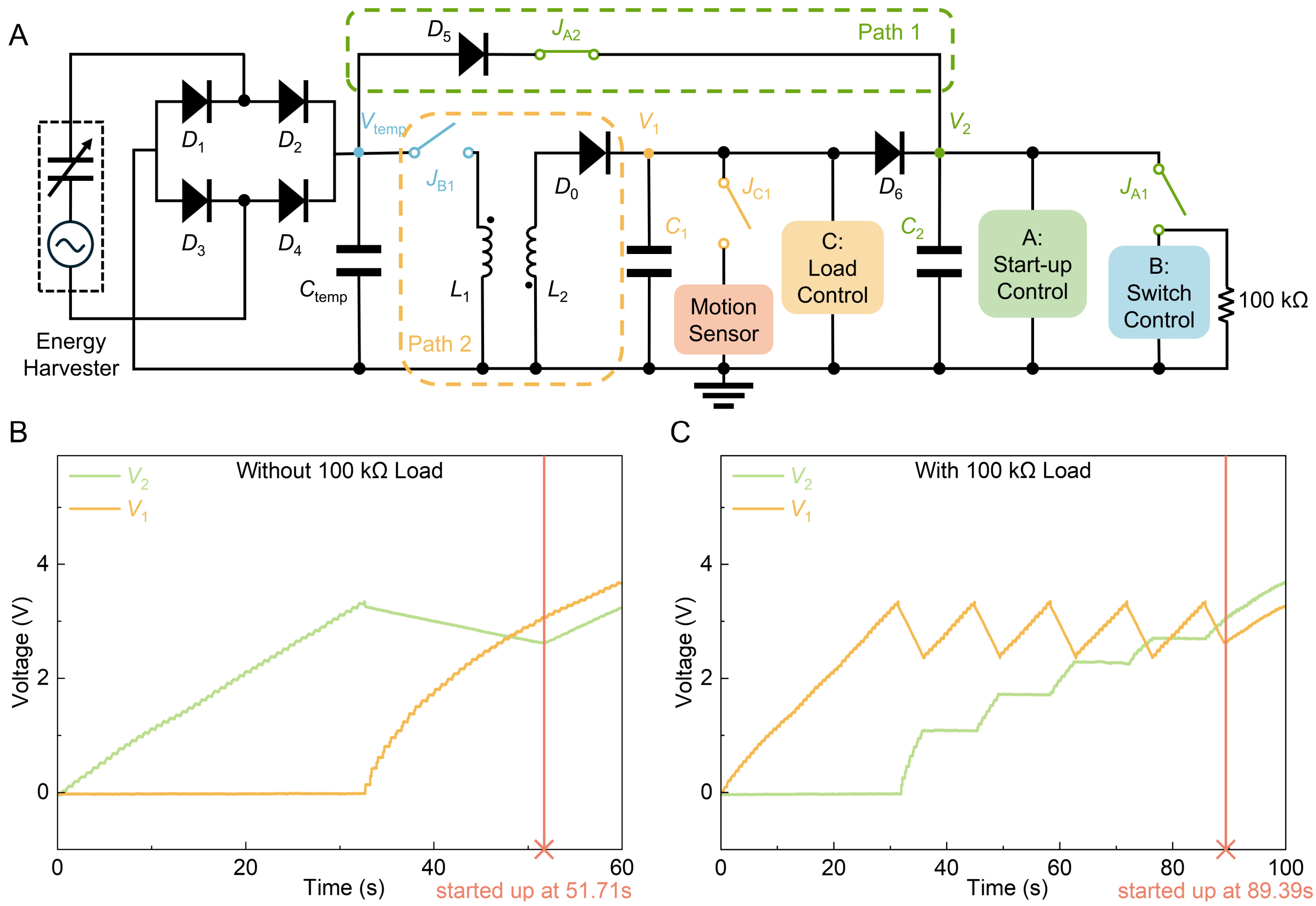

Fig. S19. Effect of high quiescent current on the start-up process. (A) Circuit configuration for testing under high flyback switch control circuit (FSCC) quiescent current, where a 100-kilohm resistor was connected between the 2.5 V power supply within the switch-control circuit and GND. (B) Start-up behavior under normal quiescent current. (C) Start-up behavior under high quiescent current. Experiments were conducted with C_1 = 1 mF and C_2 = 100 μF during a continuous 90 BPM tapping process, and data were recorded by an oscilloscope; a moving-window average filter was applied to the raw signal to reduce noise.

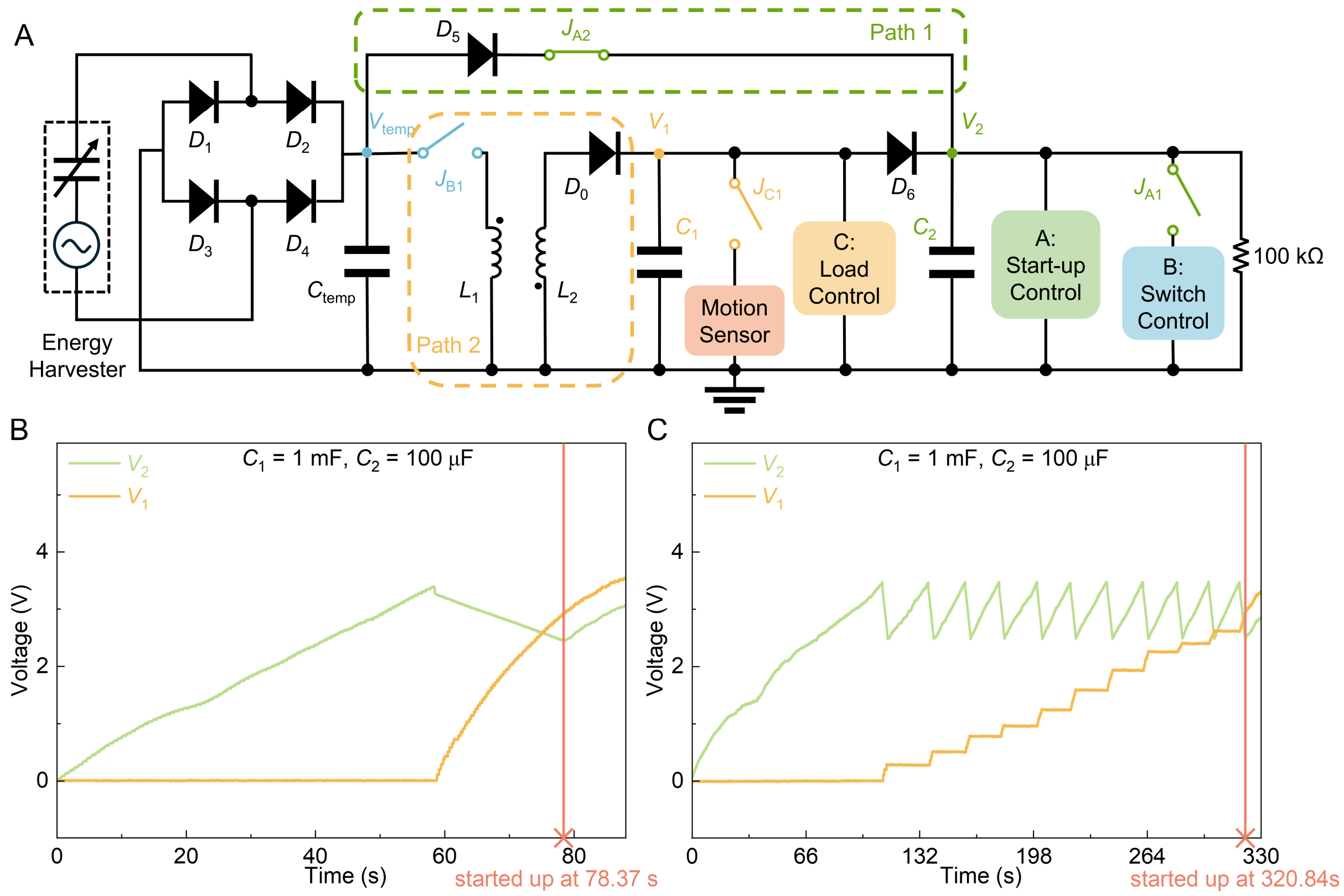

Fig. S20. Effect of high quiescent current on the start-up process. (A) Circuit configuration for testing under high start-up control circuit (SUCC) quiescent current, where a 100-kilohm resistor was connected in parallel with the capacitor C_2. (B) Start-up behavior under normal quiescent current. (C) Start-up behavior under high quiescent current. Experiments were conducted with C_1 = 1 mF and C_2 = 100 μF during a continuous 90 BPM tapping process, and data were recorded by an oscilloscope; a moving-window average filter was applied to the raw signal to reduce noise.

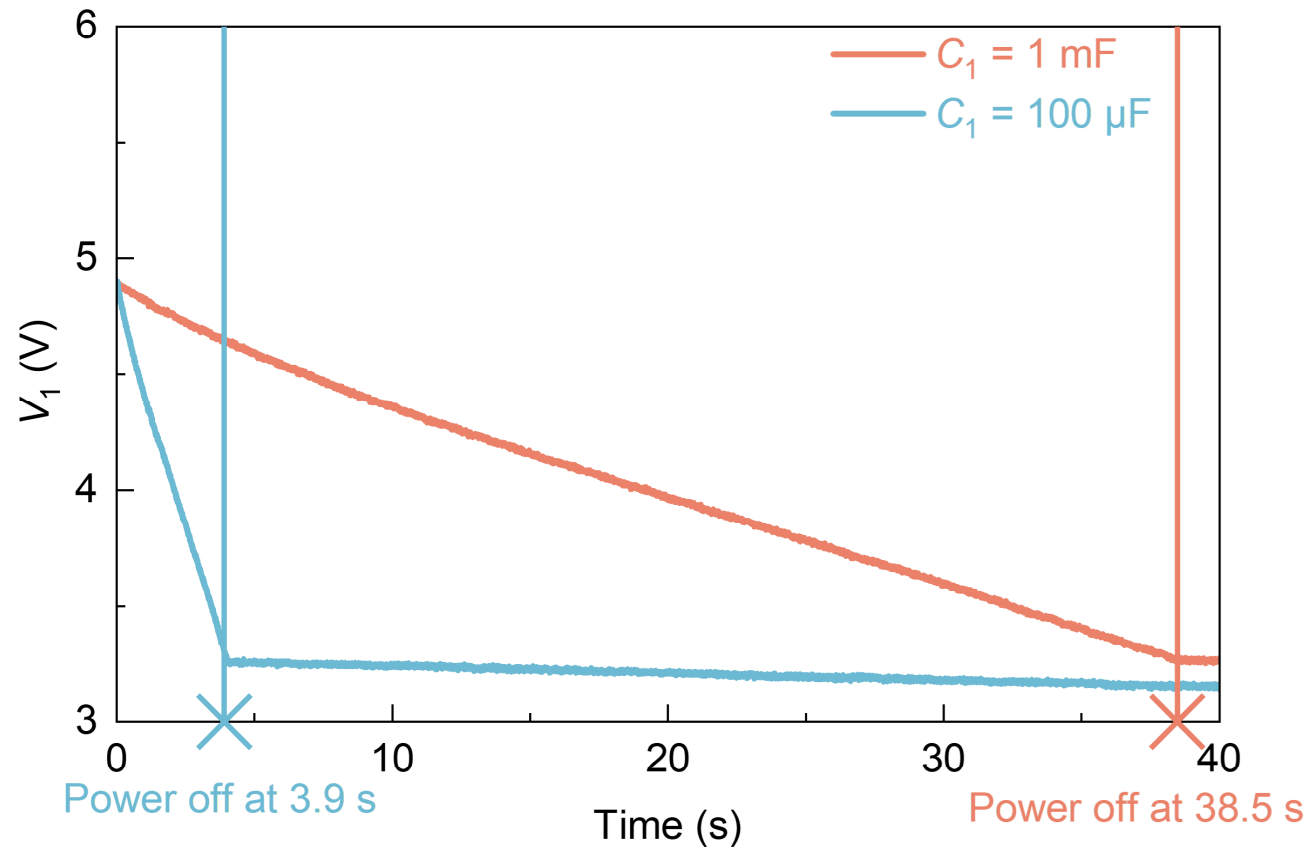

Fig. S21. Effect of C_1 capacitance on the post-supply operation. Duration of system operation after power input removal, measured for different C_1 capacitance values. Voltage data were recorded by an oscilloscope and processed with a moving-window mean filter to reconstruct the waveform.

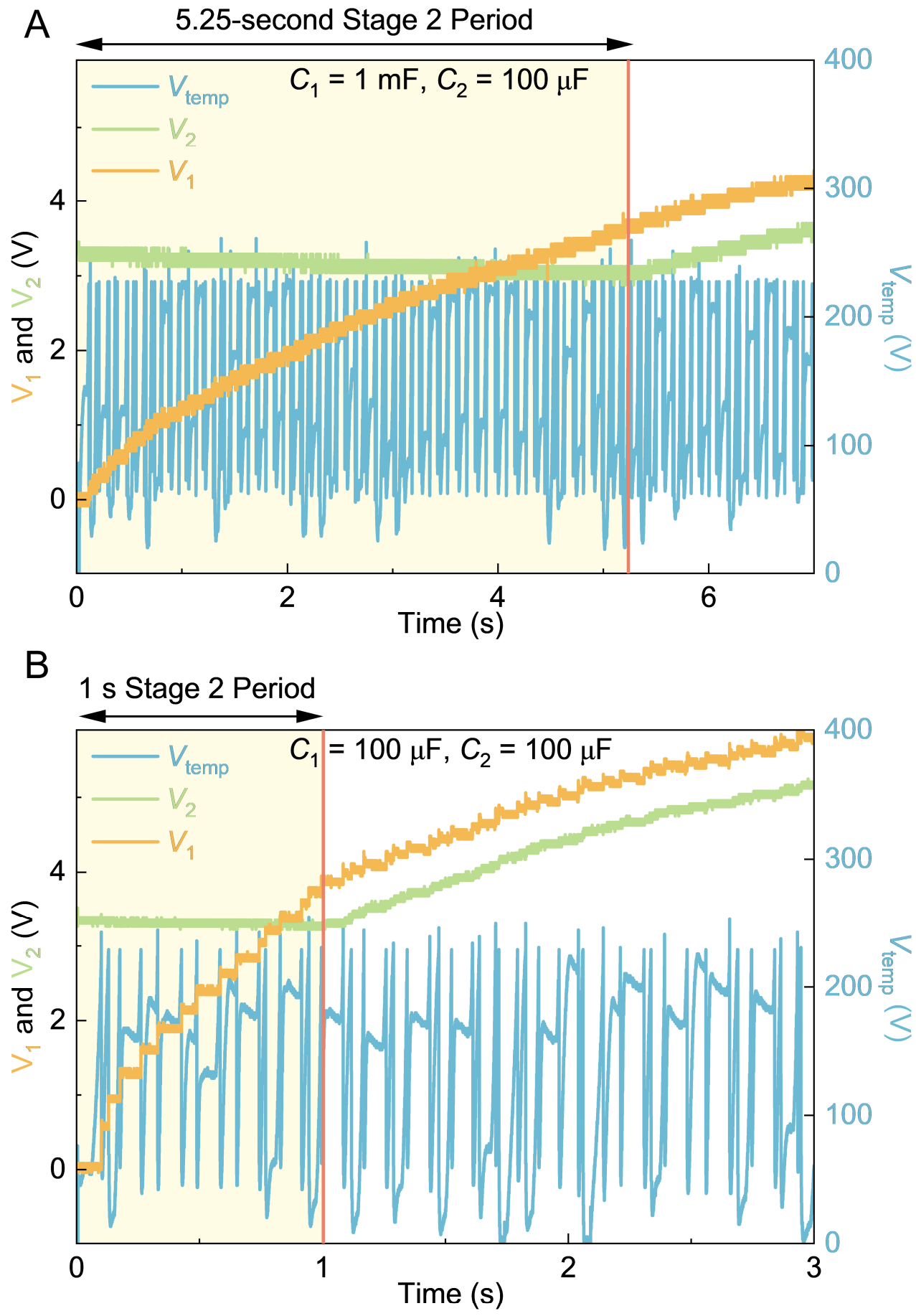

Fig. S22. Start-up behavior during charging of C_1 to the operational voltage threshold. Oscilloscope-recorded start-up waveforms showing the process of charging C_1 during Stage 2 under maximum-frequency tapping, for different C_1 capacitance values.

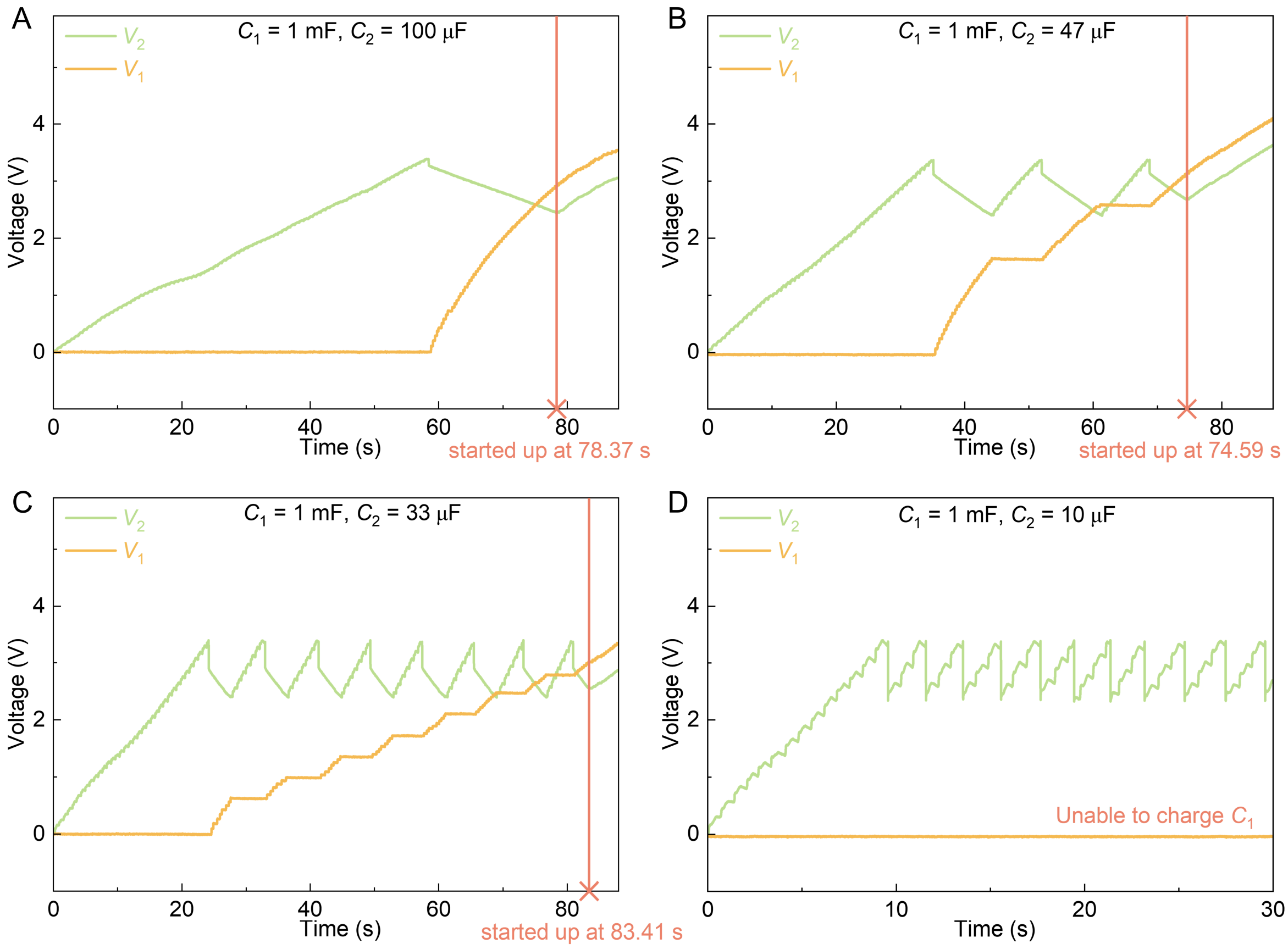

Fig. S23. Start-up behavior with varying C_2 capacitance values. Oscilloscope-recorded start-up waveforms measured for different C_2 capacitance values. Experiments were conducted during a 90 BPM tapping process. A moving-window mean filter was applied to reduce noise and reconstruct the true waveform.

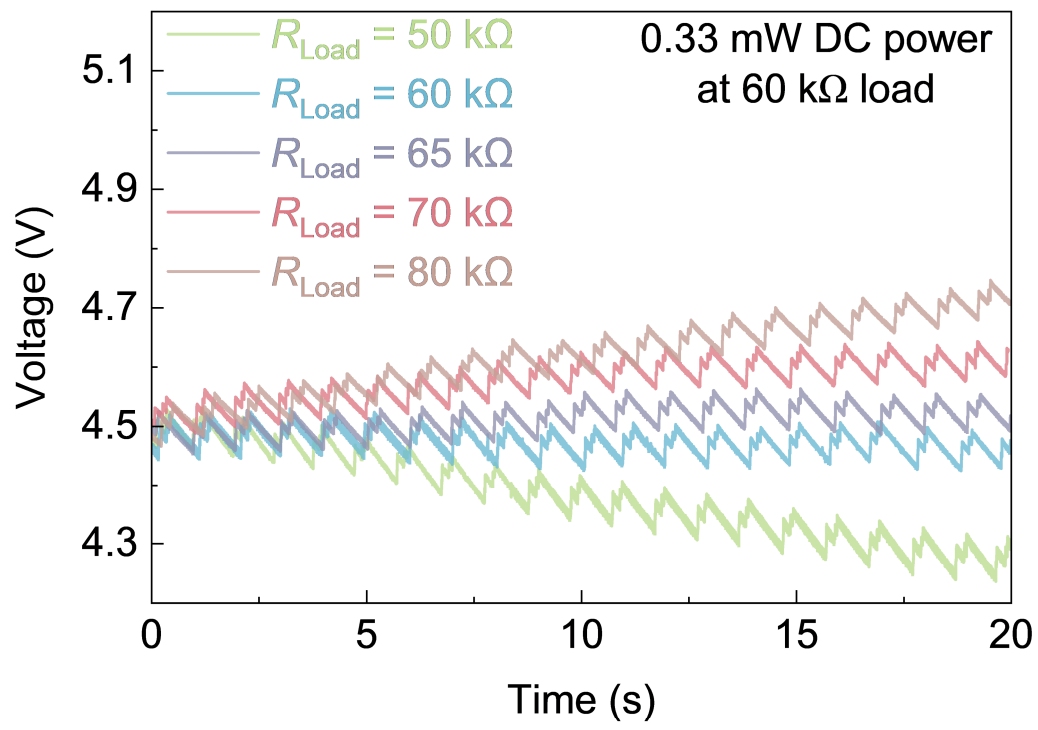

Fig. S24. DC power output under biomechanical energy input. Measured DC power output of the system driven by human biomechanical energy. The voltage shown here is the voltage of V_1. DC power is calculated as V_1^2 / R_{load} when the slope of V_1 is averaged as 0. Experiments were conducted with C_1 = 1 mF and C_2 = 100 μF during a 60 BPM tapping process.

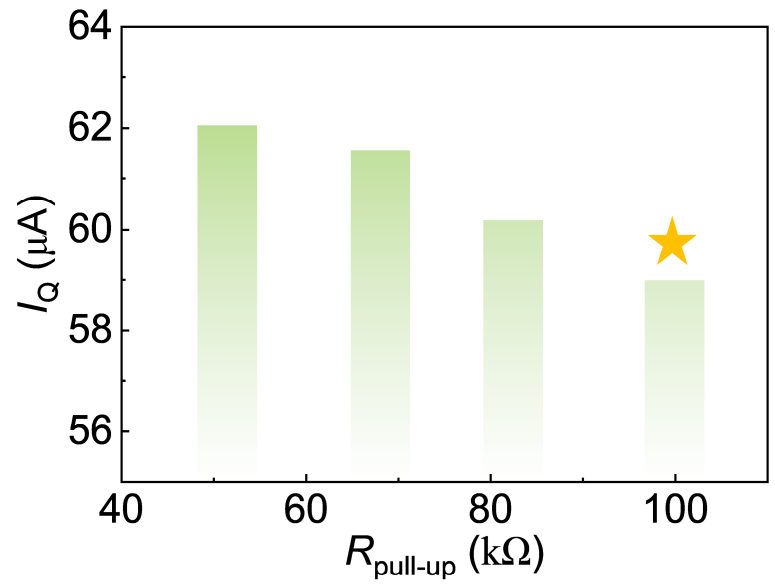

Fig. S25. I²C pull-up resistor value optimization to minimize the power consumption during I²C serial communication. Experiments were conducted using the firmware with only data collection functionality before final optimization. No edge-AI calculation or display-driving function is included in this firmware.

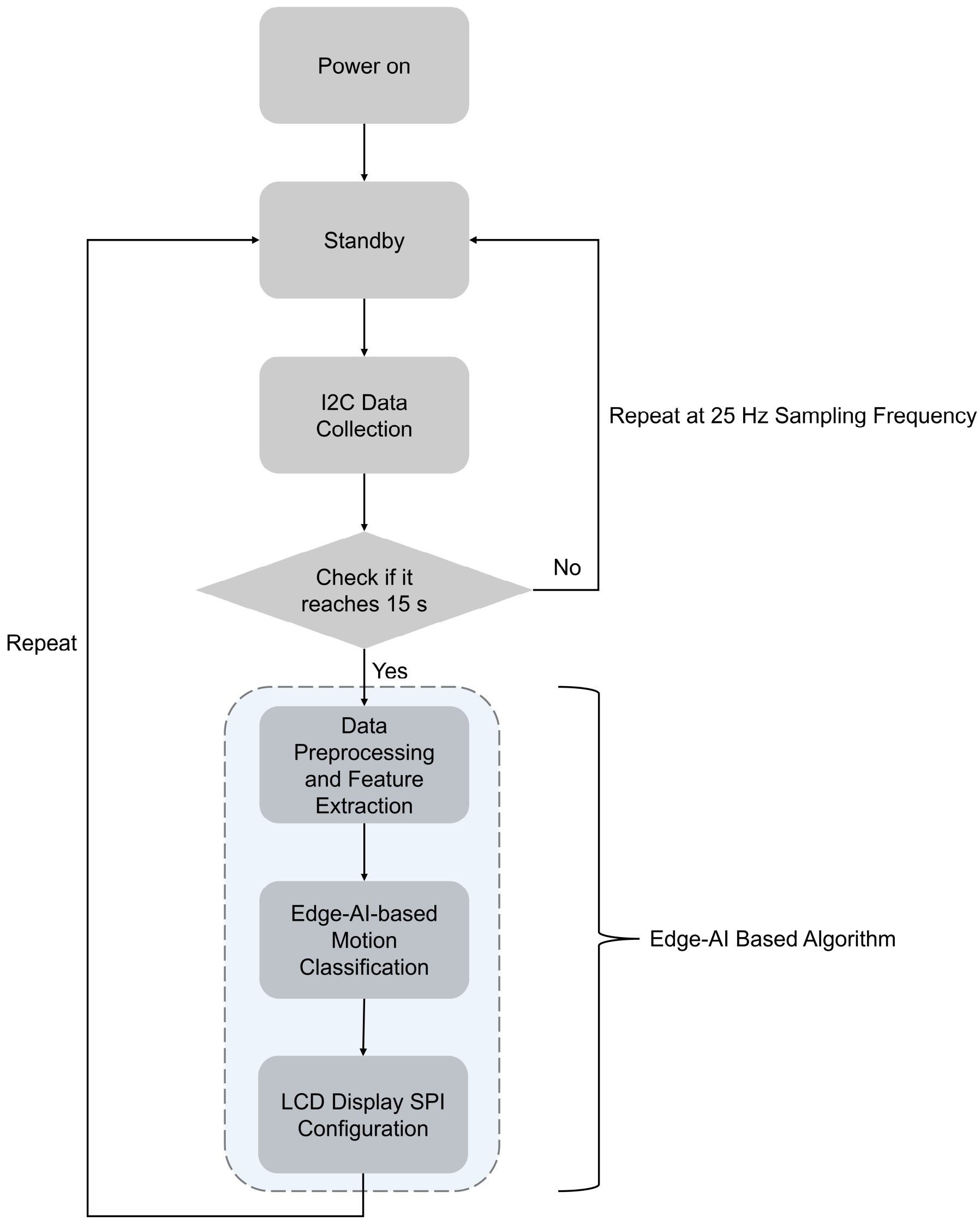

Fig. S26. Working flow of the final firmware.

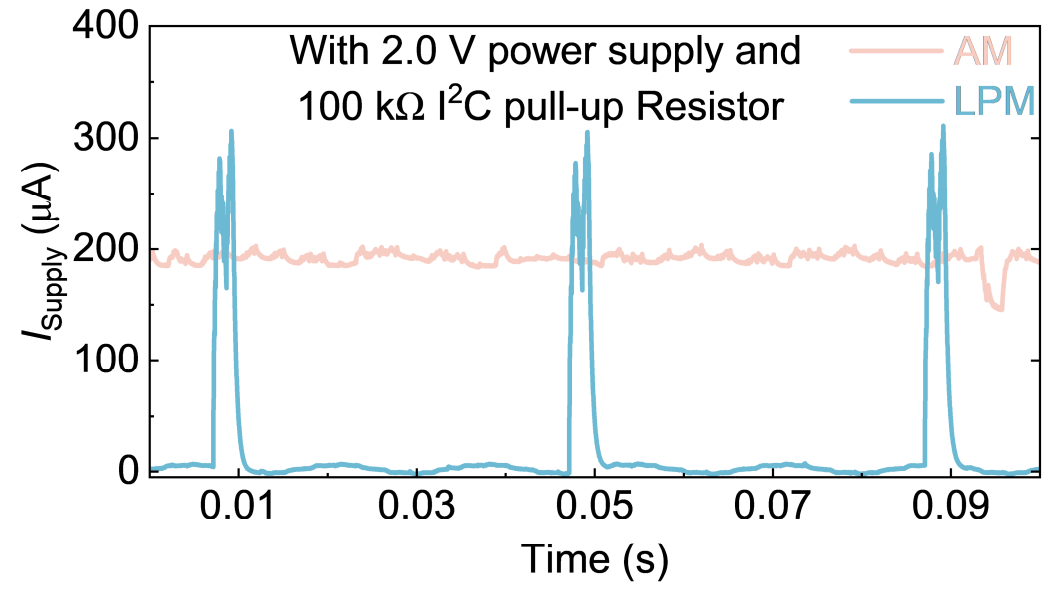

Fig. S27. Representative supply current traces comparing LPM and AM during the continuous data acquisition stage. Experiments were conducted using the firmware with only data collection functionality. No edge-AI calculation and display driving function is included in this firmware.

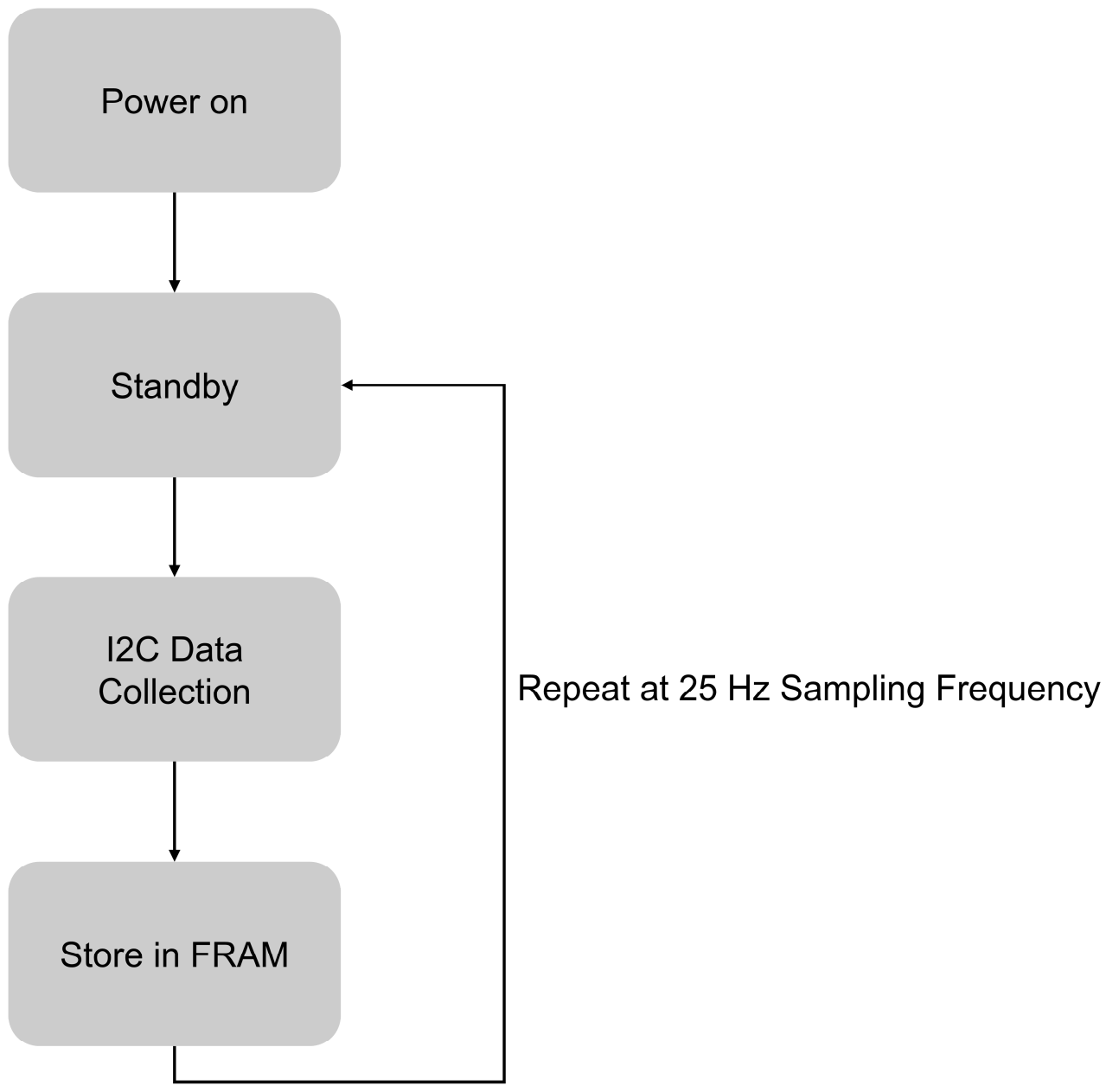

Fig. S28. Working flow of the firmware with only data collection functionality.

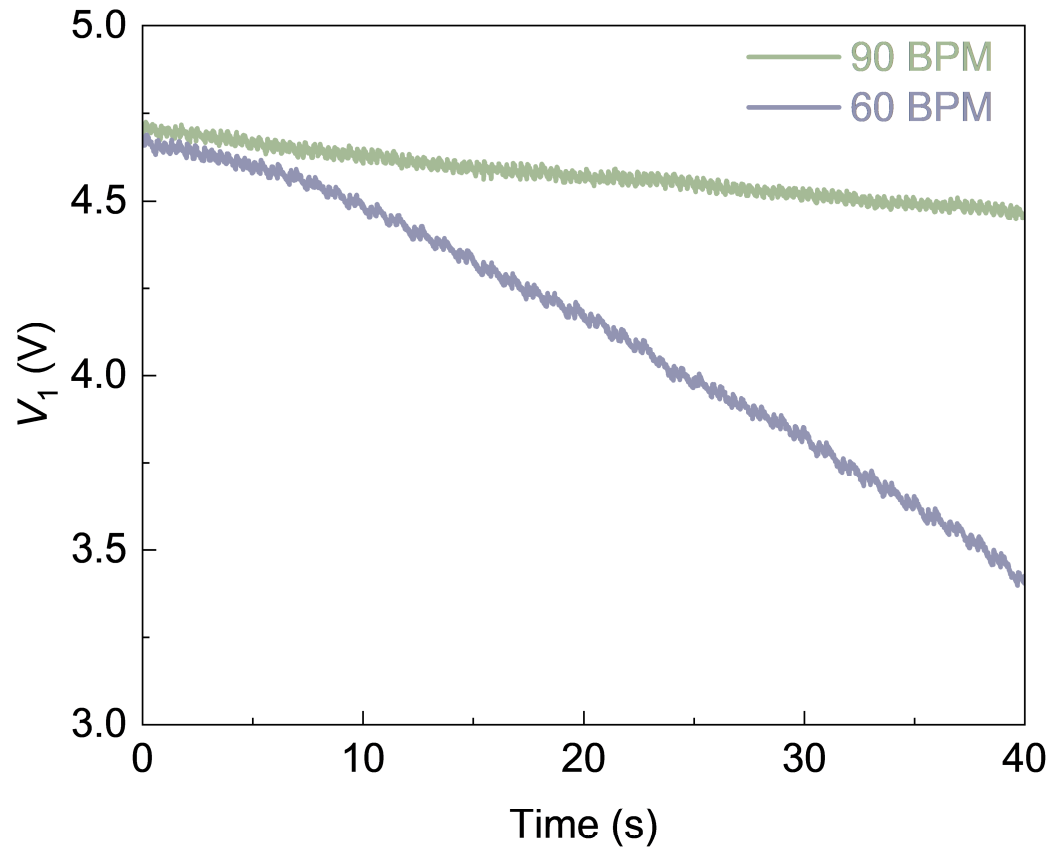

Fig. S29. Storage capacitor voltage V_1 profiles at different tapping frequencies when a data-collection firmware with only active mode is used. Oscilloscope-recorded voltage waveforms of the storage capacitor during active mode operation at tapping frequencies of 60 and 90 beats per minute (BPM). Experiments were conducted with C_1 = 1 mF and C_2 = 100 μF. Due to the large power consumption of the active mode, even 90 BPM tapping cannot support the continuous operation. The slope of V_1 is negative, which means the energy supply is smaller than the energy consumed by the load. For comparison, if we use LPM to achieve the same functionality, only 12 BPM is sufficient to sustain the operation. Experiments were conducted using firmware that utilizes I^2C communication to perform continuous data acquisition, which records 3-axis accelerometer data at a 25 Hz sampling frequency and stores it in the FRAM of the MCU. No edge-AI calculation and display driving function is included in this firmware.

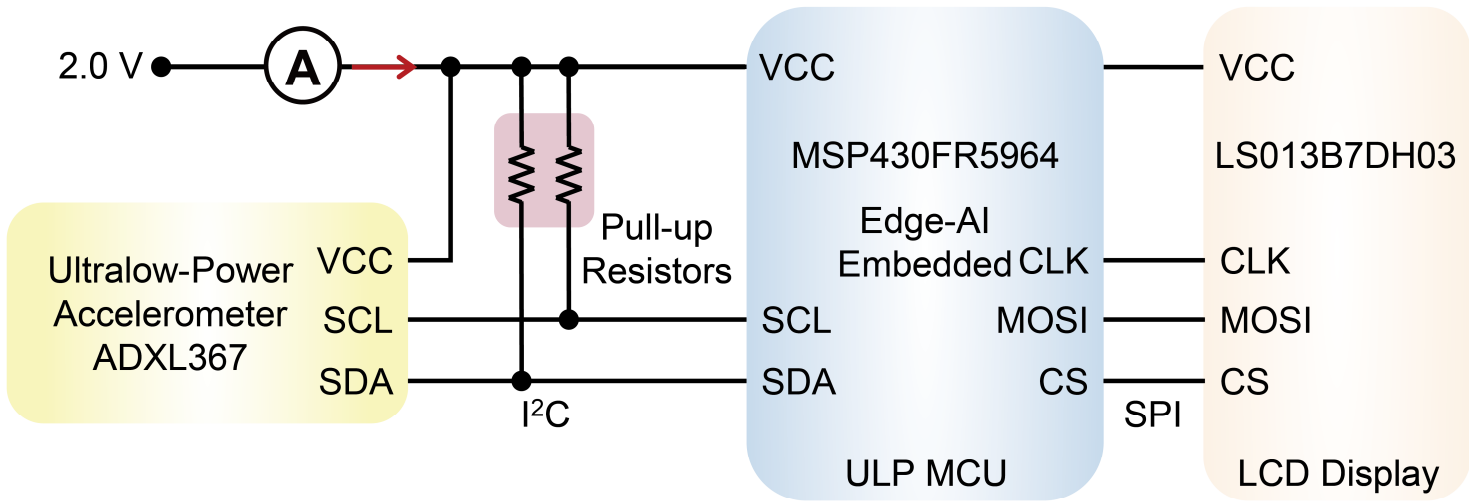

Fig. S30. The power-consumption measurement setup for the ULP motion sensor module

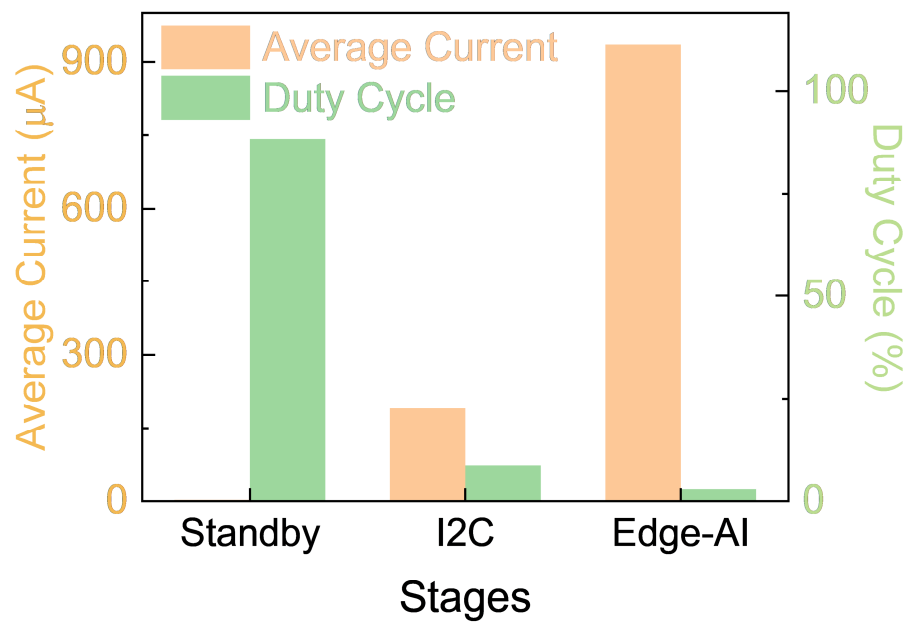

Fig. S31. Stage-specific average current consumption and duty cycle using our final firmware with a 10-second data acquisition window. The system stays in an LPM standby state (3 µA supply current) for 88.3% of the time, stays in serial communication state (190 µA supply current) for 8.7% of the time, and stays in edge-AI computation state (935 µA supply current) for 2.9% of the time. In this case, the overall average current of the ULP motion sensor module is **46.5 µA**. Measurements were conducted using our final firmware with a 10-second data acquisition window. Compared to the final firmware version with a 15-second data acquisition window, the edge-AI processing time reduces since the 10-second data acquisition window results in less data to process. However, the standby state time period also reduces. As a result, the overall supply current is larger than the firmware with a 15-second data acquisition window.

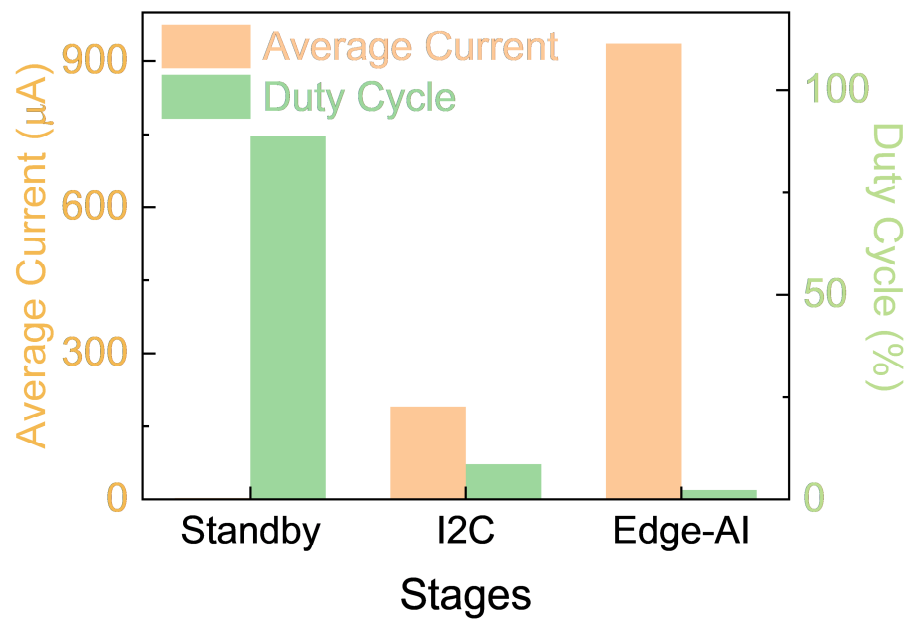

Fig. S32. Stage-specific average current consumption and duty cycle using our final firmware with a 20-second data acquisition window. The system stays in an LPM standby state (3 μA supply current) for 88.9% of the time, stays in serial communication state (190 μA supply current) for 8.8% of the time, and stays in edge-AI computation state (935 μA supply current) for 2.3% of the time. In this case, the overall average current of the ULP motion sensor module is **41.3 μA**. Measurements were conducted using our final firmware with a 20-second data acquisition window. Compared to the version with a 15-second data acquisition window, the edge-AI processing time increases since the 20-second data acquisition window results in more data to process. As the standby state time period also increases, the final overall supply current is smaller than the firmware with a 15-second data acquisition window.

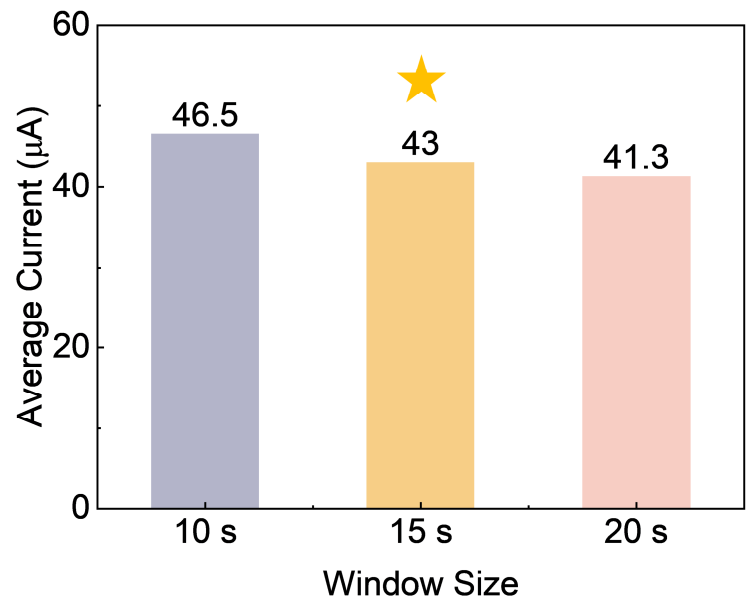

Fig. S33. Average current consumption comparison using Firmware II with different data acquisition windows. The firmware with a 20-second data acquisition window has the smallest supply current. Nevertheless, considering the trade-off between data volume and timely user feedback, we adopted a 15 s data-acquisition window as the final setting.

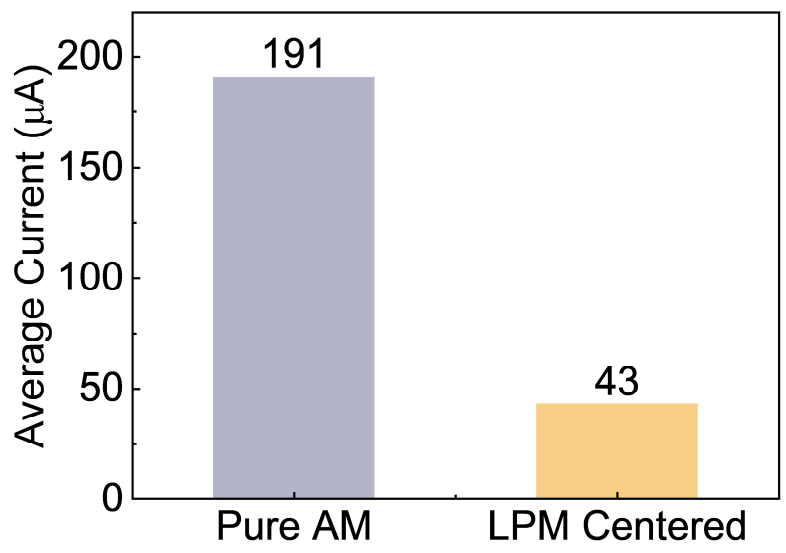

Fig. S34. Comparison of overall average current between an LPM–centered method and a pure active-mode operation. Measurements were conducted using our final firmware (including edge-AI processing) with a 15-second data acquisition window.

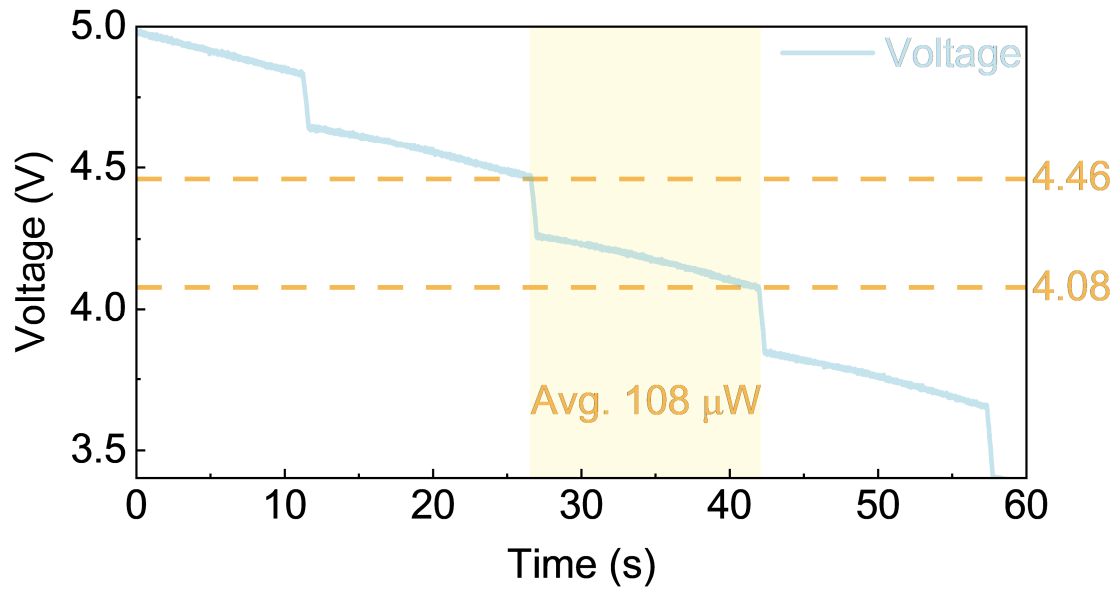

Fig. S35. Average power consumption of the complete system under the LPM-centered operating strategy. The signal was obtained using a capacitor-discharge measurement with a 1 mF storage capacitor C_1, and the highlighted region corresponds to one complete sampling-and-processing cycle. Measurements were conducted using our final firmware with a 15-second data acquisition window.

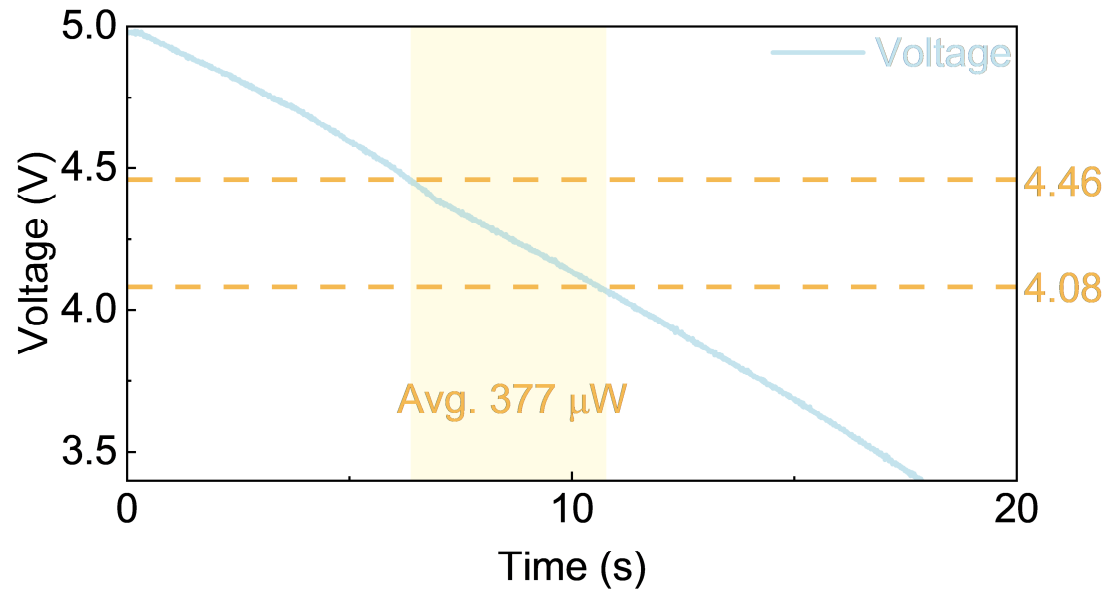

Fig. S36. Average power consumption of the complete system under the pure AM operating strategy. The signal was obtained using a capacitor-discharge measurement with a 1 mF storage capacitor C_1. Measurements were performed using the same final firmware architecture with a 15-s data-acquisition window, under pure AM operation.

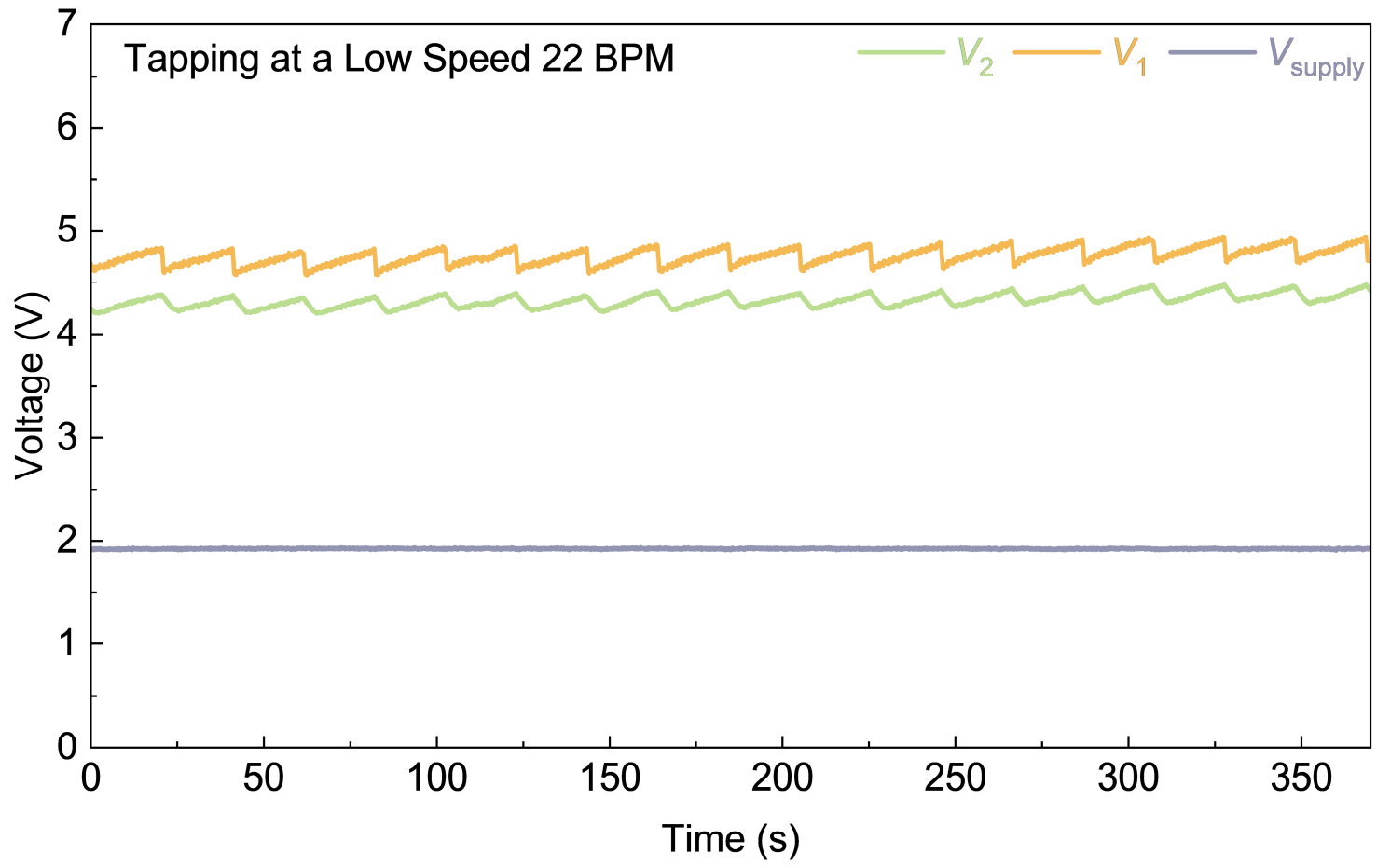

Fig. S37. Continuous powering of the motion sensor under low-speed walking. Demonstration of sustained operation for the long term. Voltage data were recorded by an oscilloscope, with a moving-window mean filter applied to reduce noise and reconstruct the true waveform. Measurements were conducted using our final firmware with a 15-second data acquisition window.

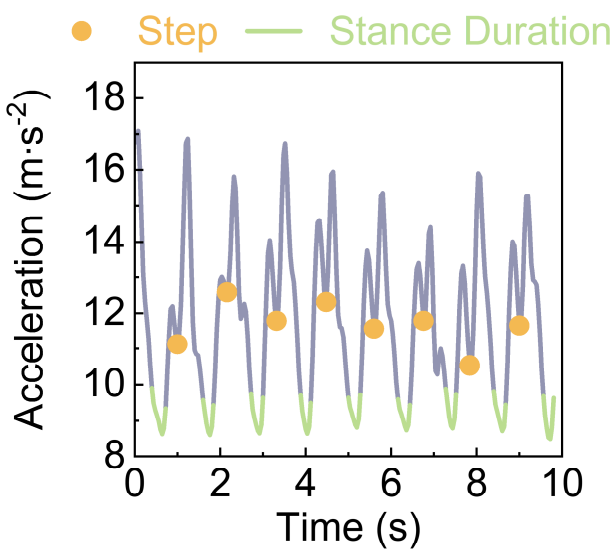

Fig. S38. Results of gait cycle identification.

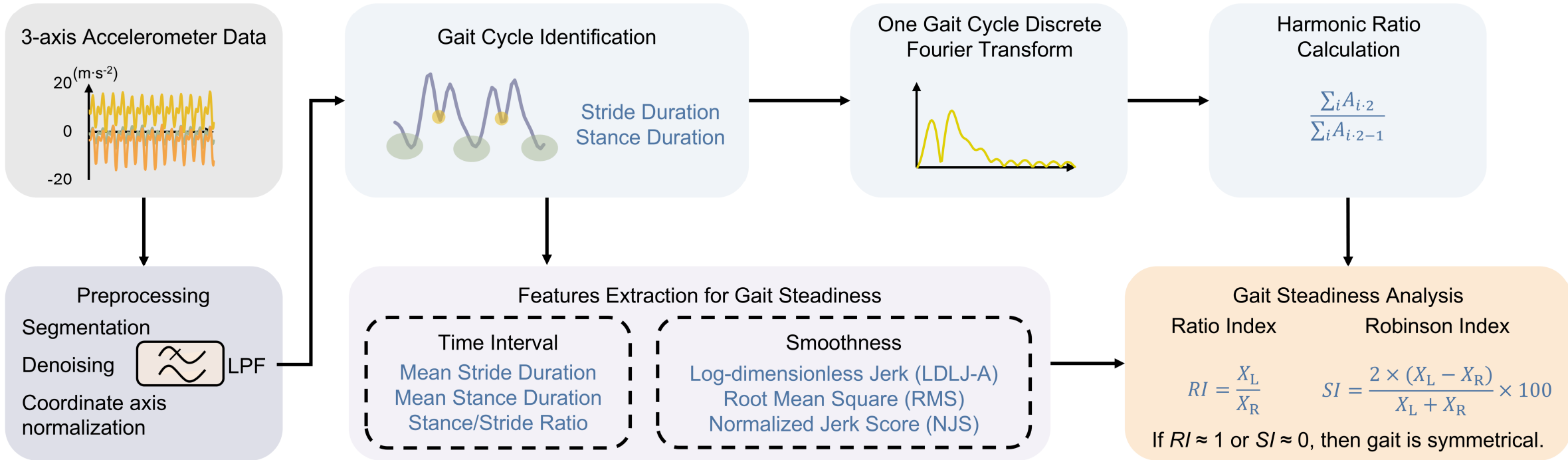

Fig. S39. Schematic representation of gait steadiness assessment algorithm.

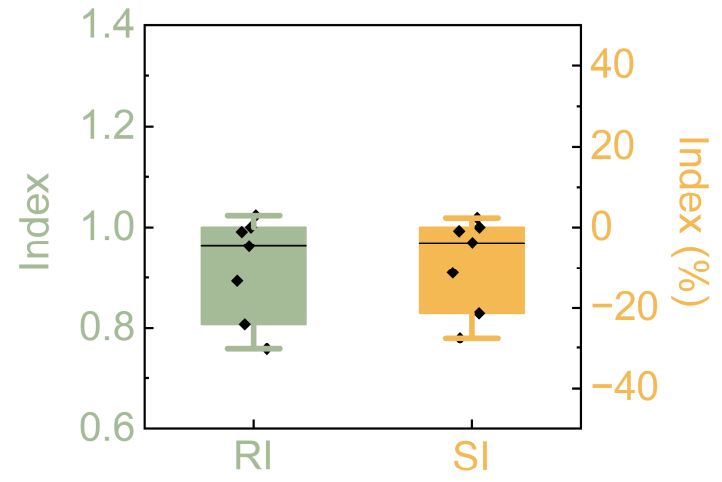

Fig. S40. Distribution of gait steadiness index values derived from the fast-walking motion state.

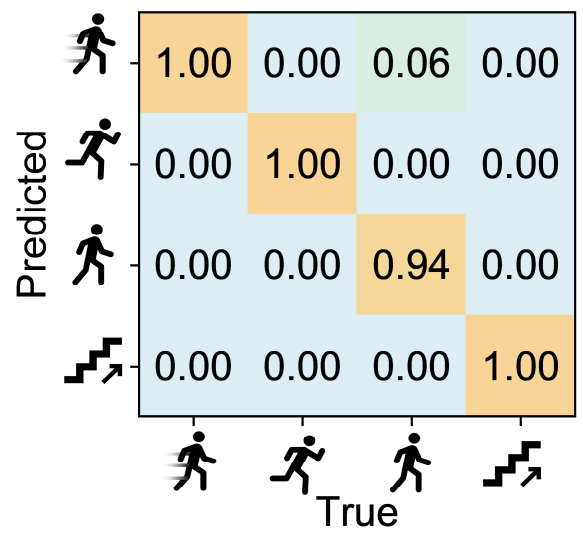

Fig. S41. Confusion matrix of motion classification before feature compression, using the full 21-feature model.

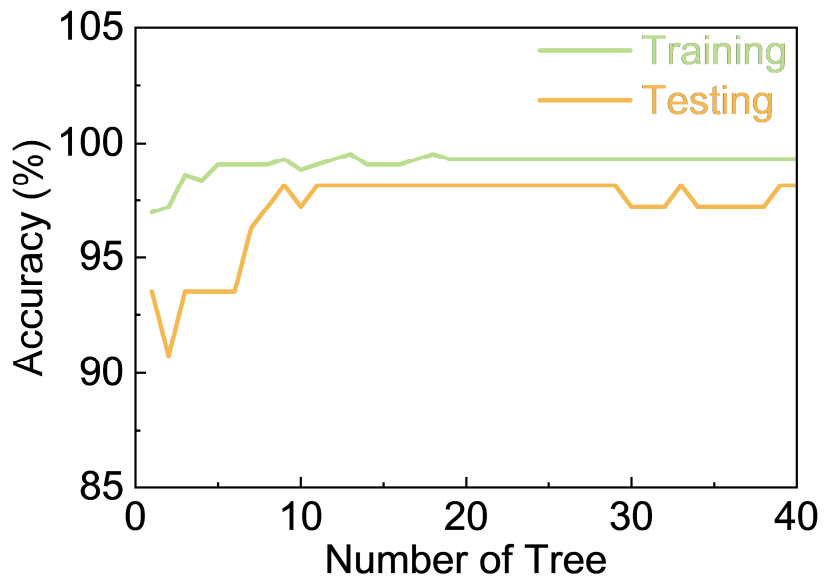

Fig. S42. Overfitting detection for the algorithm in motion classification using the full 21-feature model. Classification performance of the algorithm under varying tree numbers. Training and testing accuracy are shown.

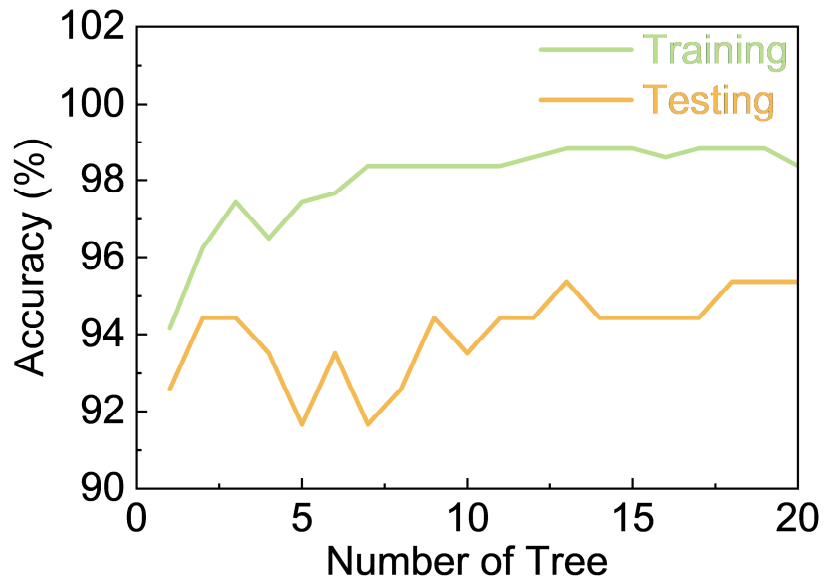

Fig. S43. Overfitting detection for the algorithm in motion classification using the final 3-feature model. Classification performance of the algorithm under varying tree numbers. Training and testing accuracy are shown.

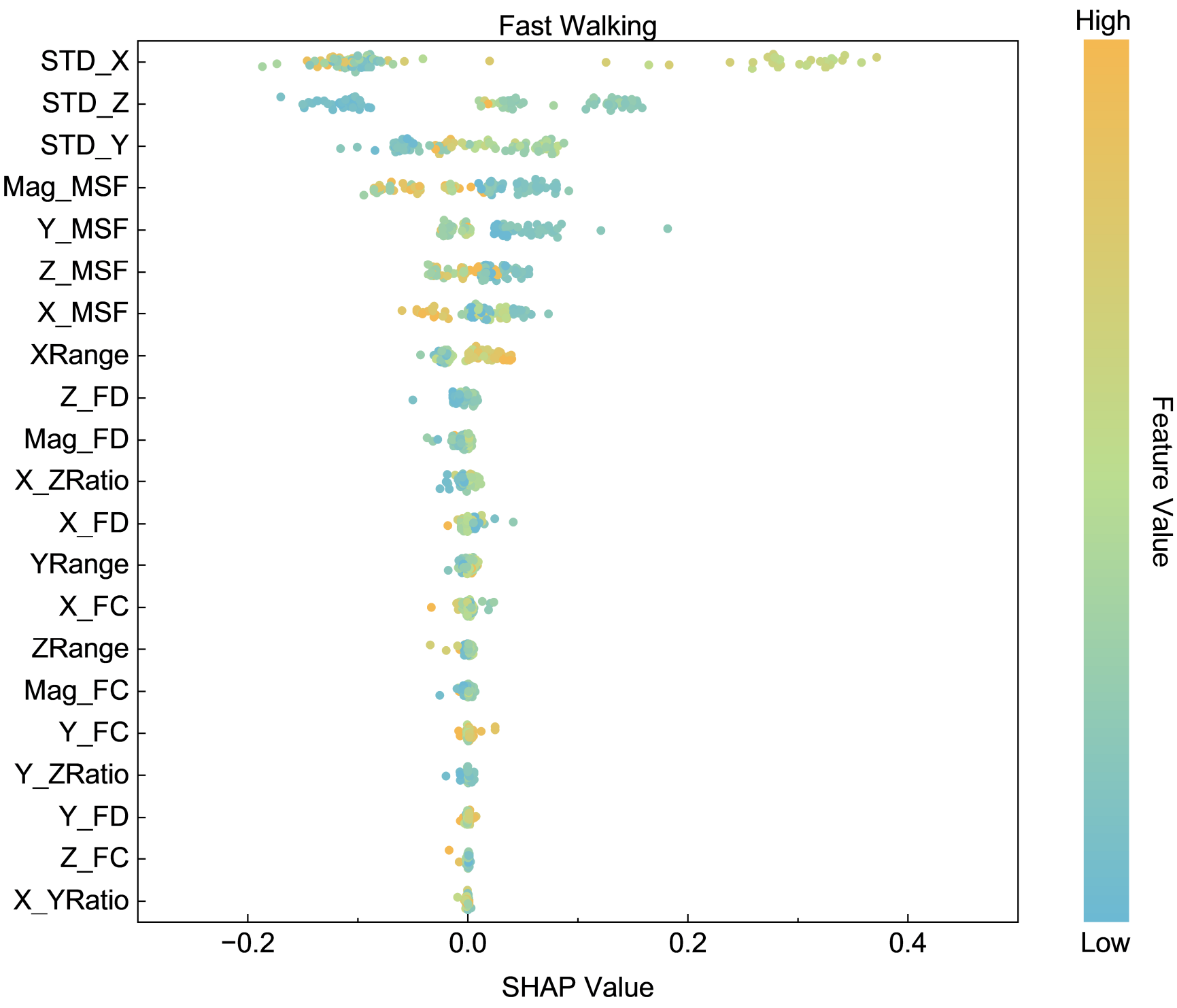

Fig. S44. SHAP Beeswarm plot showing each feature's contribution to model predictions, with samples predominantly assigned to the Fast Walking class (all 21 features shown).

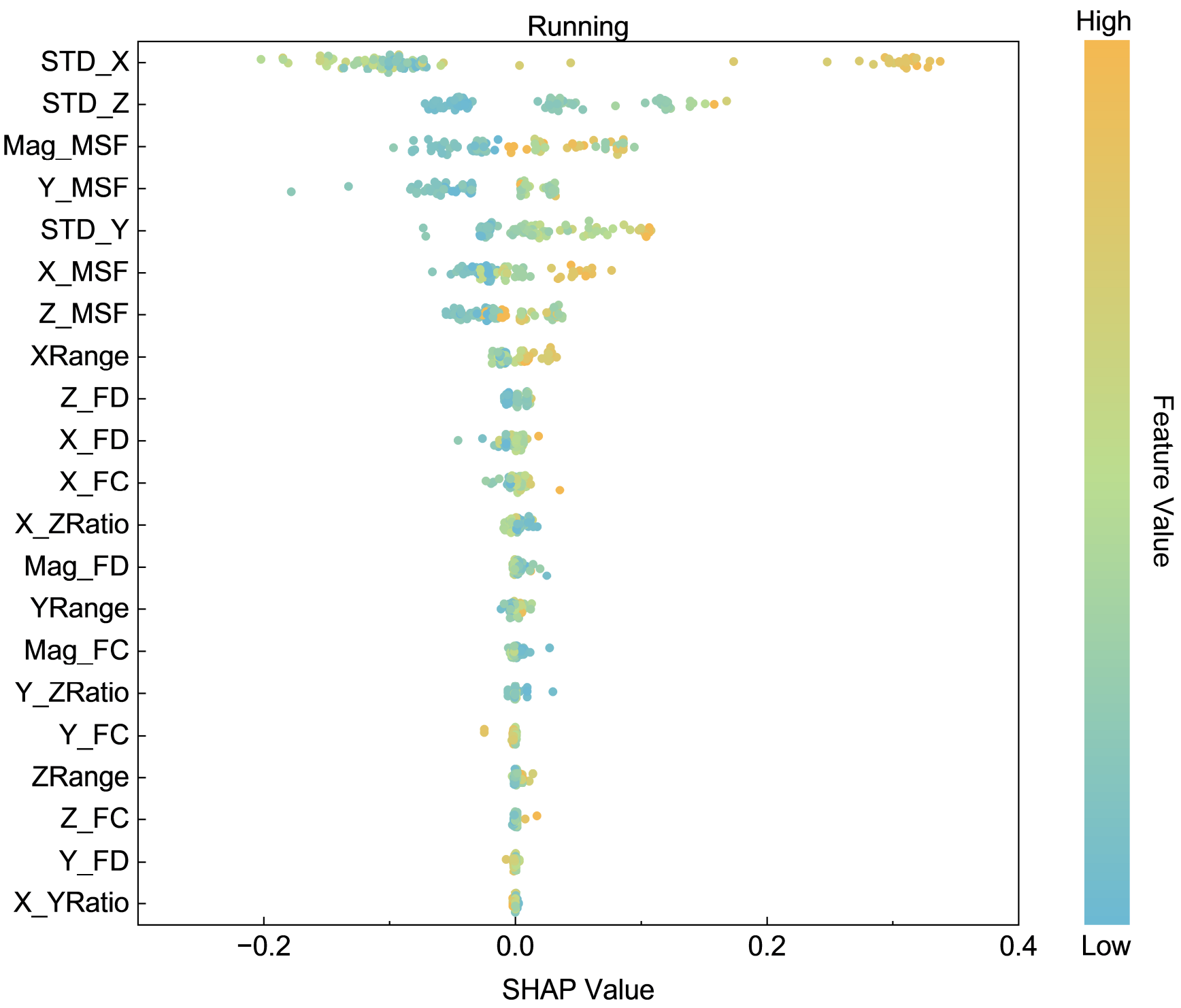

Fig. S45. SHAP Beeswarm plot showing each feature's contribution to model predictions, with samples predominantly assigned to the Running class (all 21 features shown).

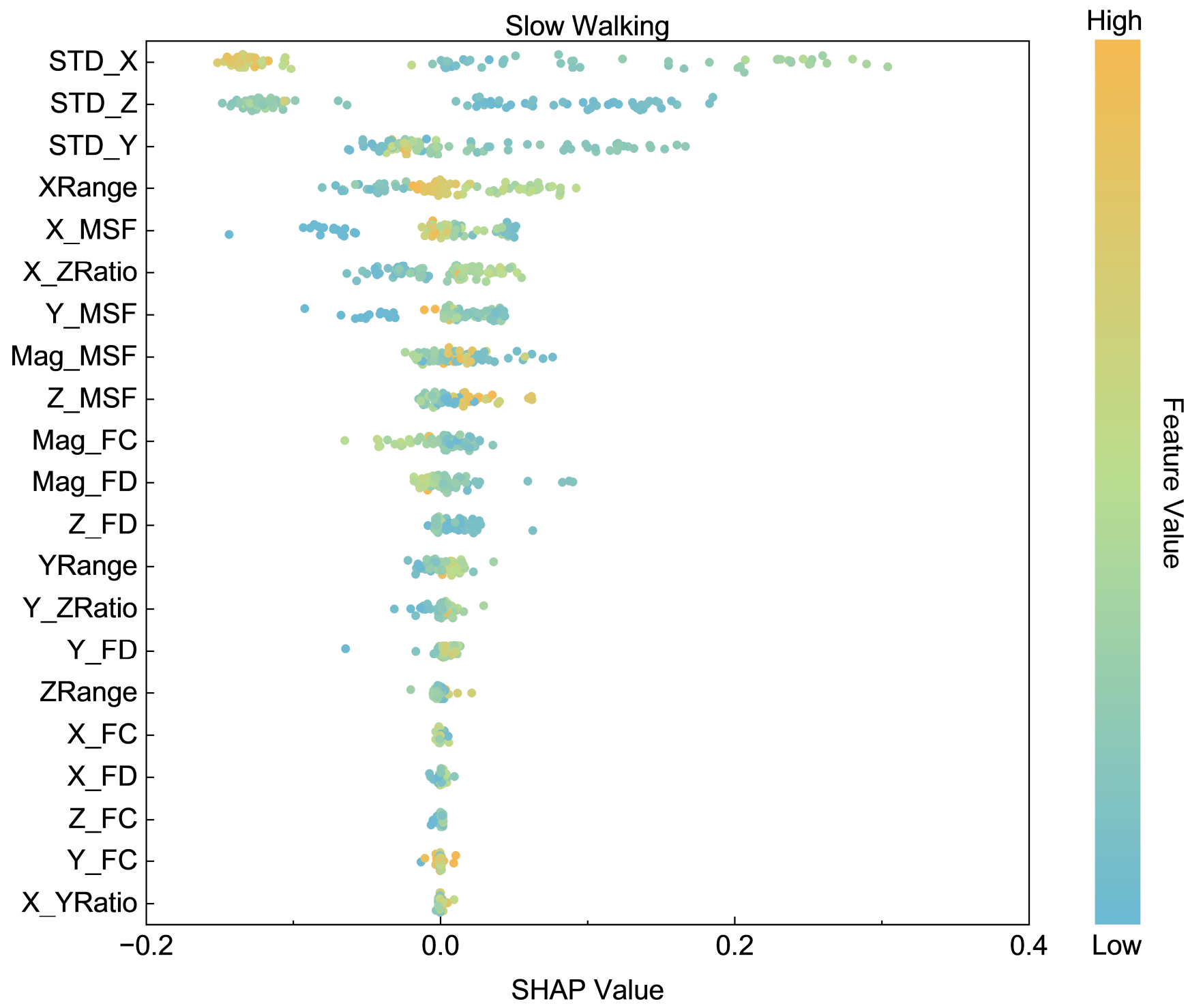

Fig. S46. SHAP Beeswarm plot showing each feature's contribution to model predictions, with samples predominantly assigned to the Slow Walking class (all 21 features shown).

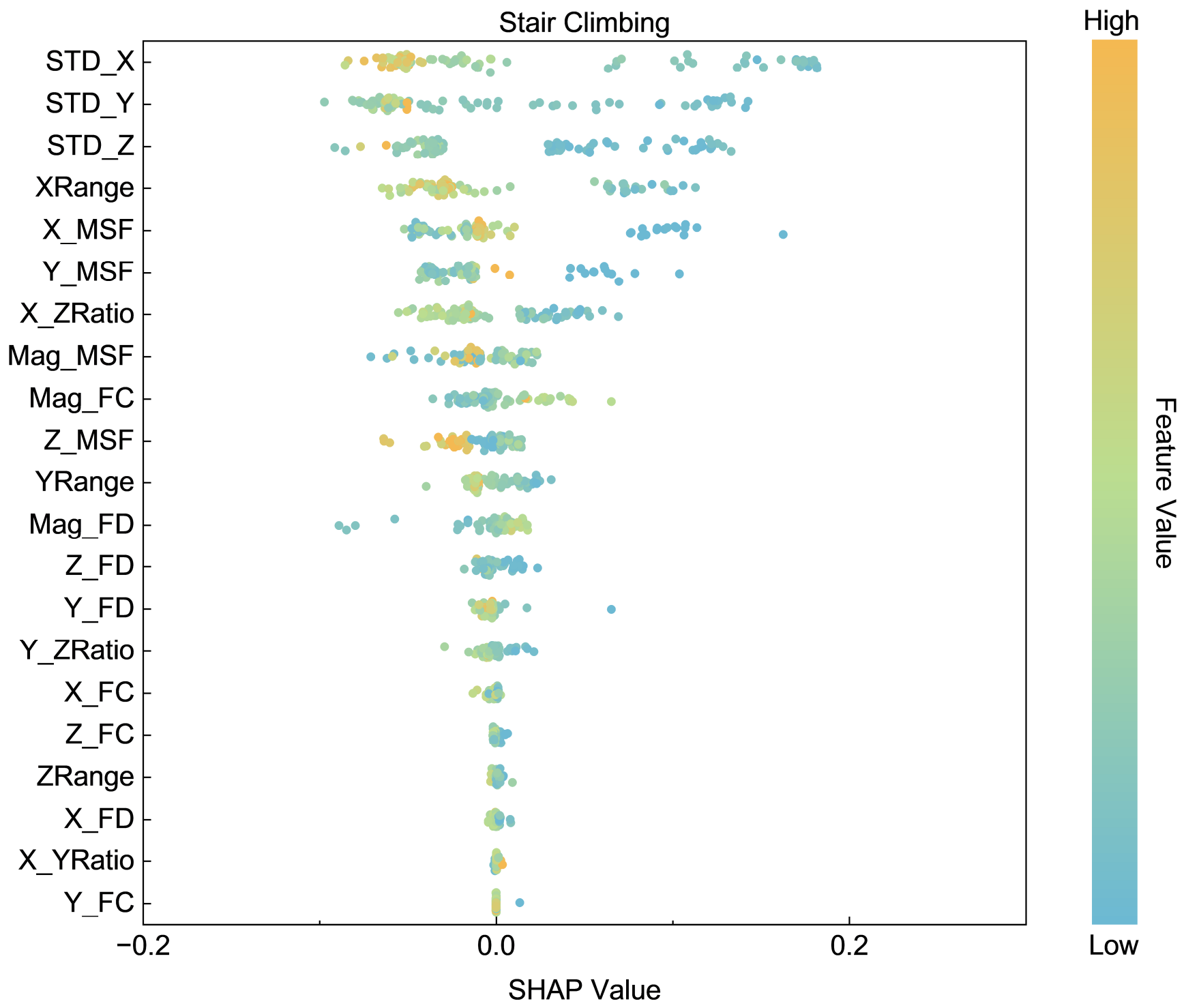

Fig. S47. SHAP Beeswarm plot showing each feature's contribution to model predictions, with samples predominantly assigned to the Stair Climbing class (all 21 features shown).

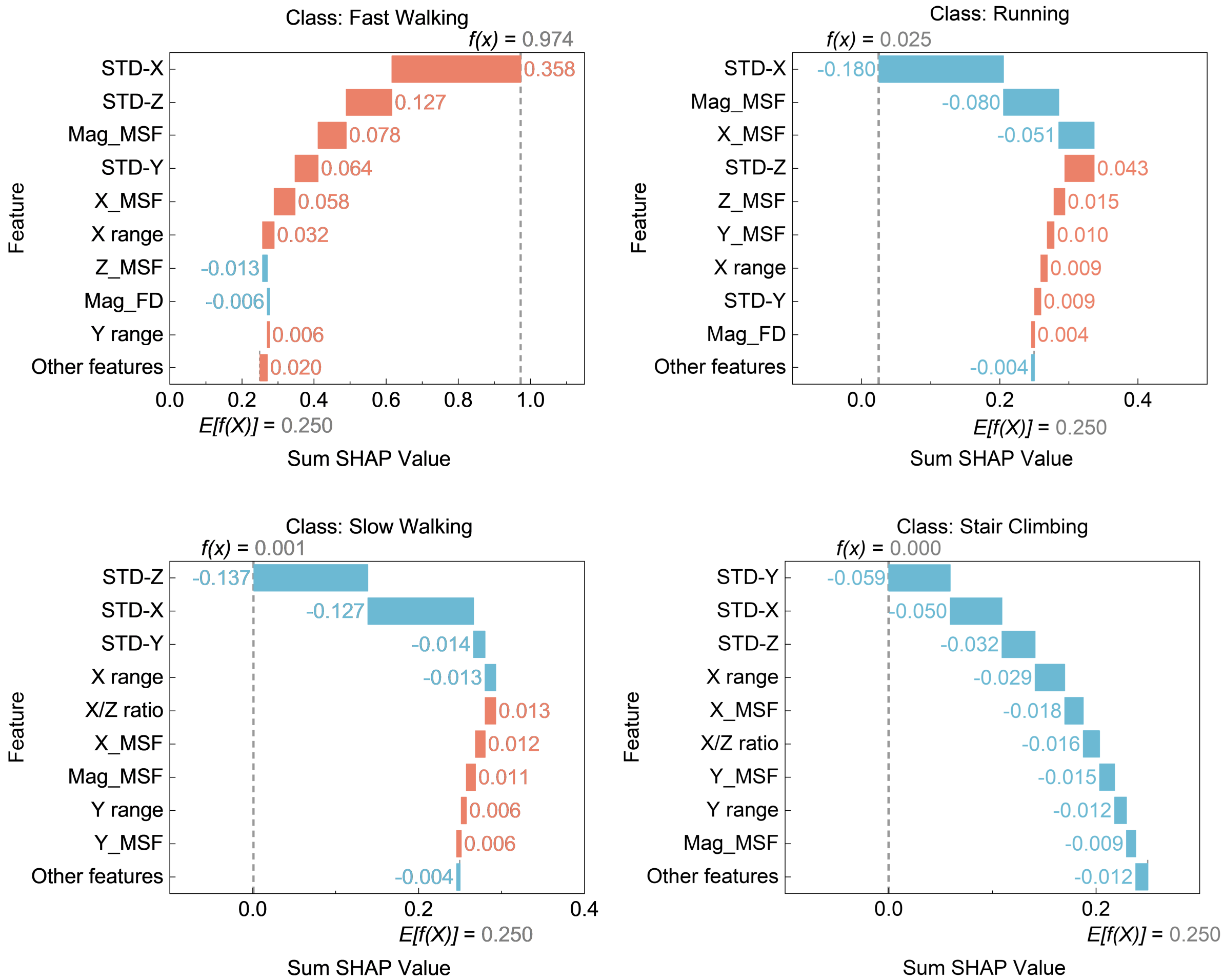

Fig. S48. SHAP waterfall explanation for a representative Fast Walking prediction using the embedded motion-classification model. The waterfall plot shows feature-wise SHAP contributions for sample ID = 0, for which the model predicts Fast Walking with probability 0.974 (Running: 0.025, Stair Climbing: 0.0, Slow Walking: 0.0), illustrating how the cumulative effects of the most influential features drive the final decision toward the Fast Walking class.

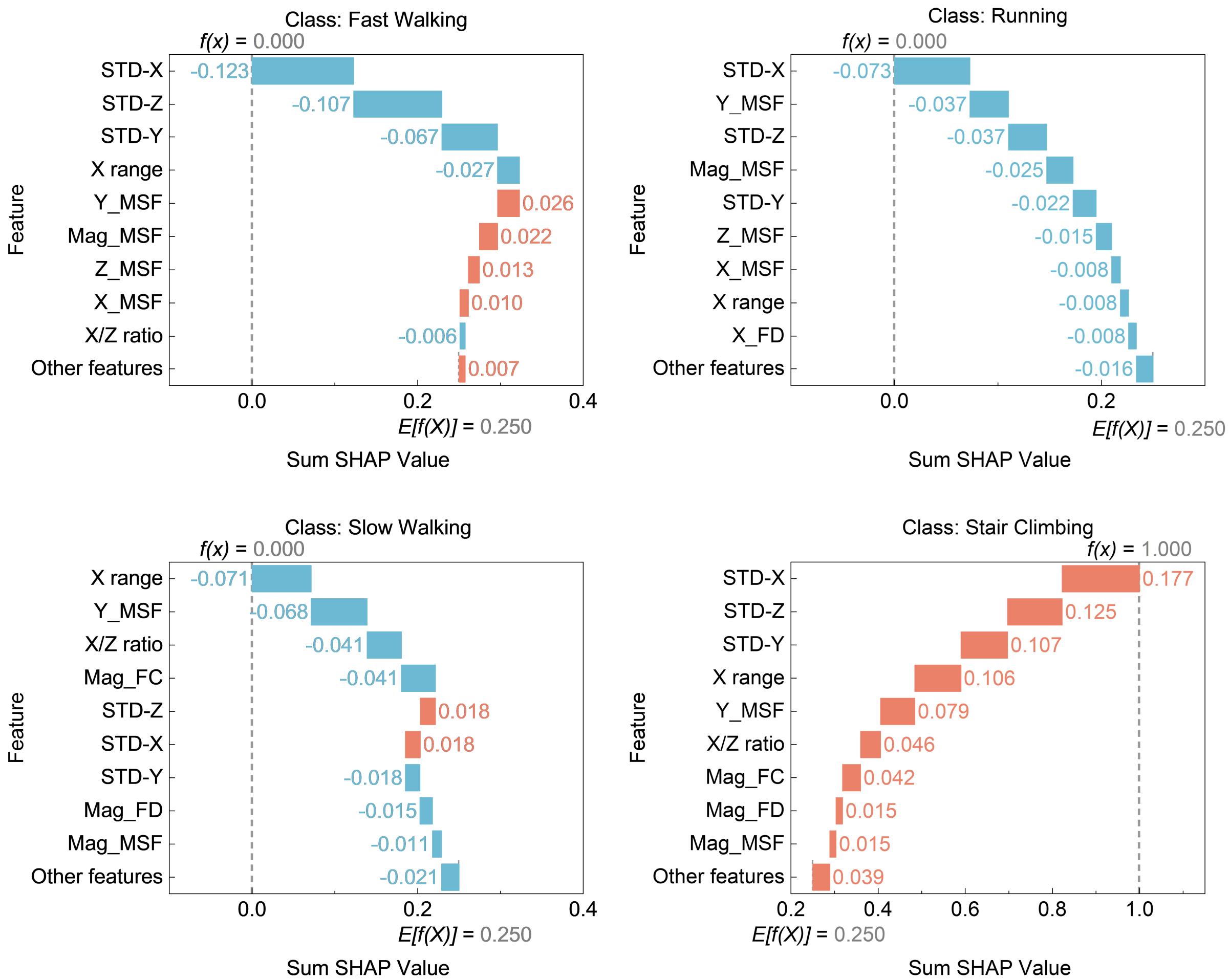

Fig. S49. SHAP waterfall explanation for a representative Stair Climbing prediction using the embedded motion-classification model. The waterfall plot shows feature-wise SHAP contributions for sample ID = 359, for which the model predicts Stair Climbing with probability 1.0 (Fast Walking/Running/Slow Walking: 0.0), illustrating how the cumulative effects of the most influential features drive the final decision toward the Stair Climbing class.

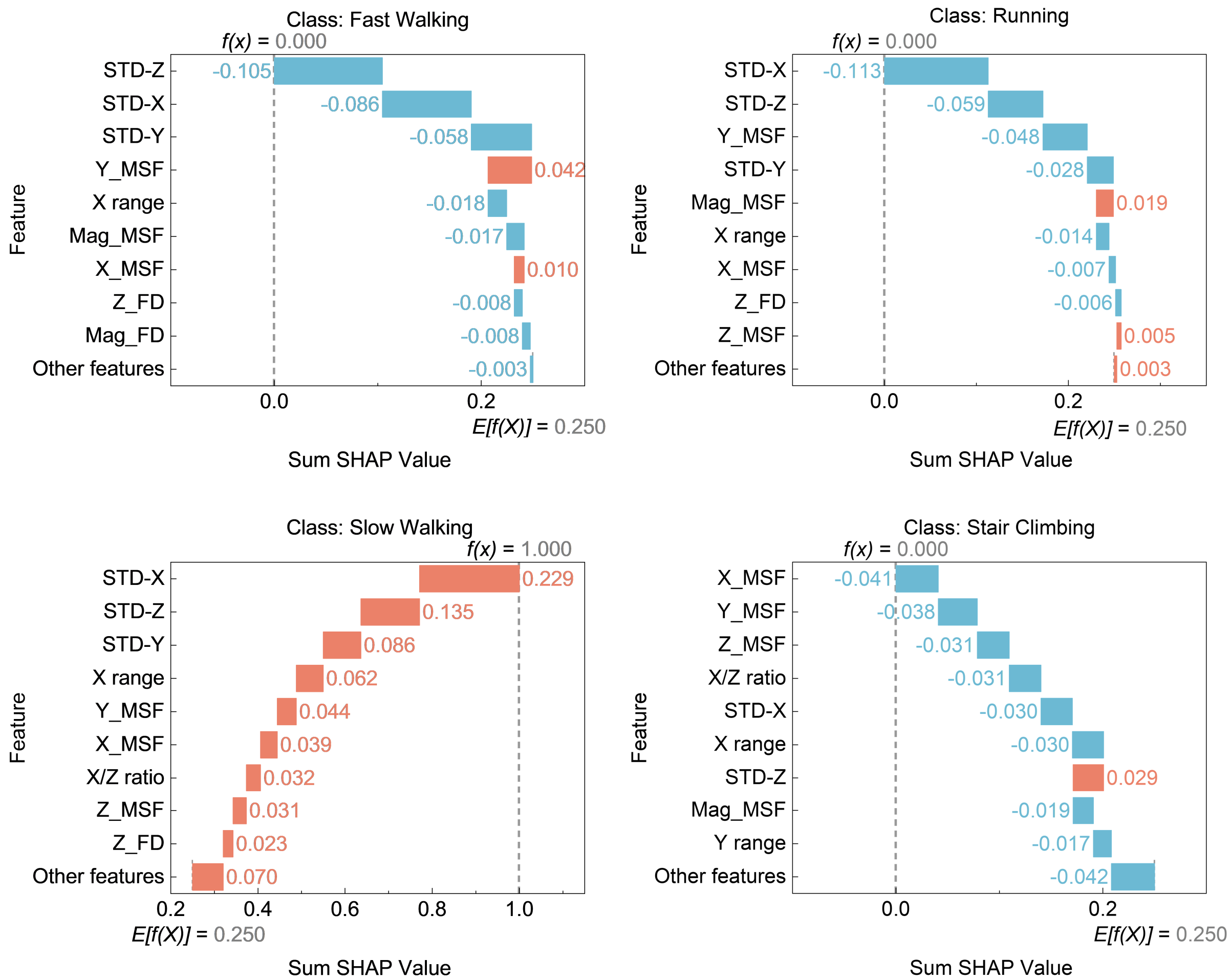

Fig. S50. SHAP waterfall explanation for a representative Slow Walking prediction using the embedded motion-classification model. The waterfall plot shows feature-wise SHAP contributions for sample ID = 492, for which the model predicts Slow Walking with probability 1.0 (Fast Walking/Running/Stair Climbing: 0.0), illustrating how the cumulative effects of the most influential features drive the final decision toward the Slow Walking class.

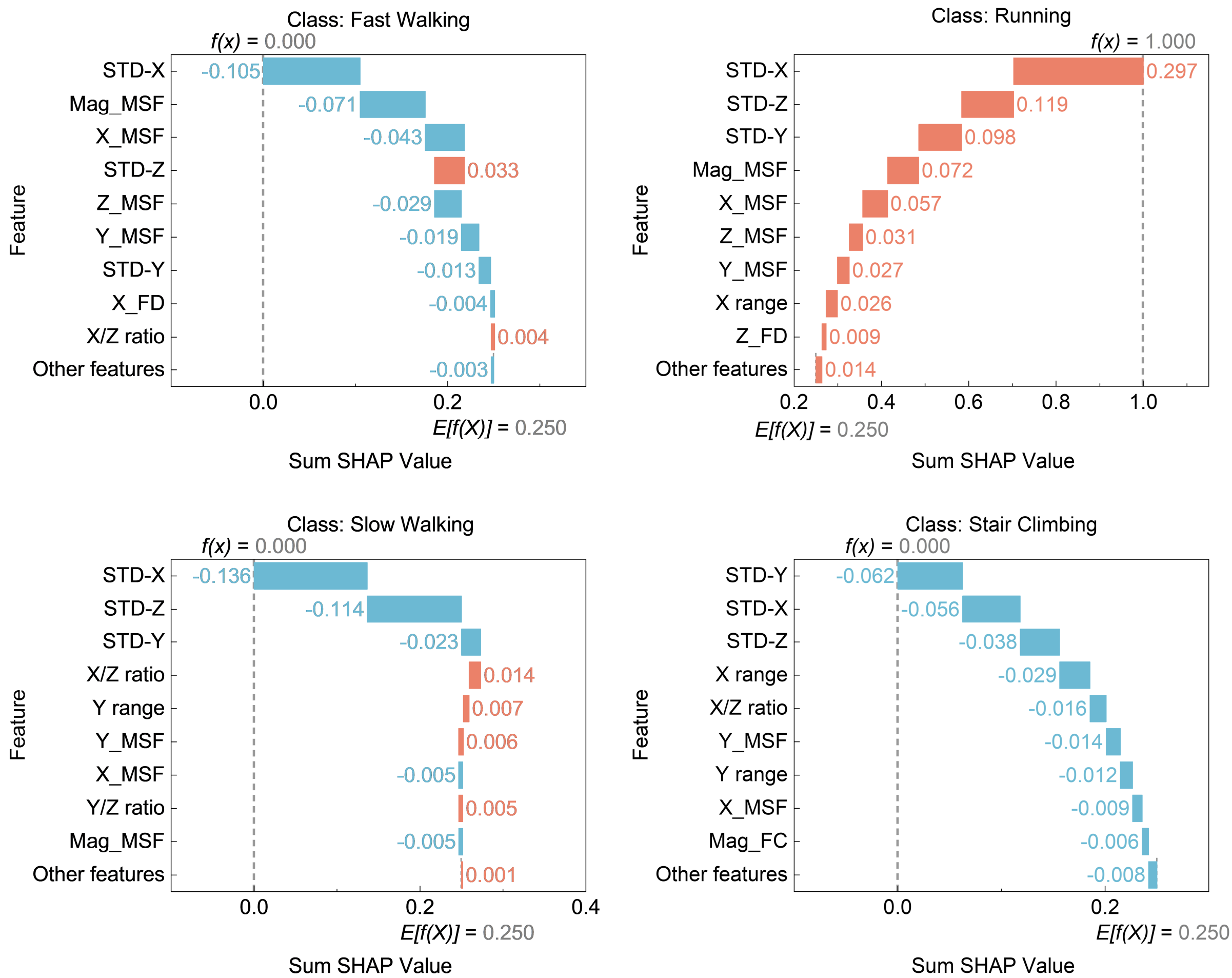

Fig. S51. SHAP waterfall explanation for a representative Running prediction using the embedded motion-classification model. The waterfall plot shows feature-wise SHAP contributions for sample ID = 204, for which the model predicts Running with probability 1.0 (Fast Walking/Slow Walking/Stair Climbing: 0.0), illustrating how the cumulative effects of the most influential features drive the final decision toward the Running class.

Work	Application	Sensing modality	Power source	On-device AI inference	Power consumption	Self-sustaining
(*24*)	EEG speech-imagery interface	EEG	Battery	Yes	25.93 mW	No
(*25*)	Multimodal biosignal acquisition and edge-AI	EEG/EMG/ECG/PPG	Battery	Yes	32.8 mW headband; 26.7 mW EMG sleeve; 9.3 mW ECG-PPG chestband;	No
(*26*)	On-device frailty assessment	IMU gait signal	Wearable platform	Yes	BLE TX/RX events up to >30 mW; MCU core <8 mW active;	No
(*54*)	Multimodal wearable edge-AI platform for cough monitoring, heartbeat classification, and seizure detection	IMU, acoustic, ECG/EEG/EMG, EDA/RSP, PPG, temperature	Battery	Yes	~42 mA in all-modality storage-stream mode; 8.86–30.3 mW in different edge-AI processing tasks	No
(*55*)	Real-time gait-pattern identification	Wearable MEMS accelerometer	Battery	Yes	optimized design: 94 mW	No
(*28*)	Smart insole for gait monitoring and recognition	CNT/ACET/PDMS pressure sensor array	Flexible perovskite solar module + lithium battery energy storage	No; smartphone-side processing	40.7 mW at 32 Hz; 4.07 mW at 1 Hz; 0.33 mW without BLE	Self-sustaining under sufficient light
This work	Gait monitoring and activity recognition	Accelerometer	Biomechanical energy harvesting + cold-start PMC	Yes	86 µW	Yes

Table S1. Comparison of representative wearable edge-AI and gait-monitoring systems.

Accelerometer	Typical Operating Current	Notes
**ADXL367**	**0.89 µA**	**Ultralow power; no duty cycling; used in this work**
ADXL345	23 µA	Common in consumer-grade wearables
LIS3DH	2 µA	Widely used in low-power applications
BMA400	5.8 µA	Integrated power management features

Table S2. Comparison of power consumption among representative accelerometers in wearable electronics.

Microcontroller	Active Mode Operating Current	Notes
**MSP430FR5964**	**125 µA (1 MHz)**	**Selected for this work**
ATtiny (25/45/85)	800 µA (4 MHz）	Common in low-power systems
STM32U3 series	305 µA (1 MHz)	Ultralow-power ARM Cortex-M33
nRF52840	3.3 mA (64 MHz)	BLE-enabled, Cortex-M4

Table S3. Comparison of active mode power consumption among representative microcontrollers used in wearable electronics.

Device	Estimated Average Current Consumption	Notes
**This work's ULP motion sensor**	**43 µA**	**Ultralow-power**
Axivity AX3 (https://axivity.com/product/ax3)	~0.21 – 0.54 mA	150–180 mAh battery; 14 days @ 100 Hz; 30 days @ 12.5 Hz
Xsens DOT (https://xsens.com/hubfs/Downloads/Whitepapers/Xsens%20DOT%20WhitePaper.pdf)	~5.83 mA	70 mAh battery; up to ~12 h on-board recording
Mbientlab MetaMotionR (https://mbientlab.com/store/metamotionr/)	7.7 mA	100 mAh Li-Polymer battery; ~12.97 h estimate (accelerometer-only configuration)

*Average current = (battery capacity) ÷ (battery life in hours) when both are provided.

Table S4. Comparison of power consumption between the motion sensor developed in this work and representative commercial motion-sensor units. Similar to our work, the commercial devices listed are primarily accelerometer-based data acquisition units configured with on-board storage. The estimated average current for commercial devices is derived from manufacturer-reported battery capacity and operating life under stated settings.

Subject	Participant Information				Class of Locomotion				
	Sex	Age	Height (cm)	Weight (kg)	Slow walking	Fast walking	Running	Stair climbing	Total
**S1**	F	25	165	72	32	34	29	19	114
**S2**	M	24	180	85	33	37	29	40	139
**S3**	M	23	175	75	61	41	32	33	167
**S4**	M	26	179	93	31	34	35	17	117
**Total**	—	—	—	—	157	146	125	109	537

Table S5. Participant information and distribution of 15-s gait data windows across subjects and locomotor classes.

Trial	Left Foot Step Count	Right Foot Step Count
1	50	49
2	39	39
3	41	40
4	45	44
5	18	19
6	57	57

Table S6. Comparison of step counting results between the left and right foot over 60-second intervals. Step counts were independently extracted from left- and right-foot recordings collected during the same walking trials. A one-step difference between feet within each 60-second interval is considered negligible for practical gait analysis.

Activity class label	MET value used
Slow walking	2.0 (*46*)
Fast walking	2.5 (*46*)
Running	3.0 (*46*)
Stair climbing	8.6 (*47*)

Table S7. Mapping between activity classes and MET values

REFERENCES

1. Q. Zeng, X. Tian, D. T. Nguyen, C. Li, P. Chia, B. C. K. Tee, C. Wu, J. S. Ho, A digitally embroidered metamaterial biosensor for kinetic environments. *Nat. Electron.* **7**, 1025–1034 (2024).

2. J. Shi, S. Kim, P. Li, F. Dong, C. Yang, B. Nam, C. Han, E. Eig, L. L. Shi, S. Niu, J. Yue, B. Tian, Active biointegrated living electronics for managing inflammation. *Science* **384**, 1023–1030 (2024).

3. G. Xu, Y. Lu, C. Cheng, X. Li, J. Xu, Z. Liu, J. Liu, G. Liu, Z. Shi, Z. Chen, F. Zhang, Y. Jia, D. Xu, W. Yuan, Z. Cui, S. S. Low, Q. Liu, Battery-free and wireless smart wound dressing for wound infection monitoring and electrically controlled on-demand drug delivery. *Adv. Funct. Mater.* **31**, 2100852 (2021).

4. W. Ouyang, W. Lu, Y. Zhang, Y. Liu, J. U. Kim, H. Shen, Y. Wu, H. Luan, K. Kilner, S. P. Lee, Y. Lu, Y. Yang, J. Wang, Y. Yu, A. J. Wegener, J. A. Moreno, Z. Xie, Y. Wu, S. M. Won, K. Kwon, C. Wu, W. Bai, H. Guo, T.-L. Liu, H. Bai, G. Monti, J. Zhu, S. R. Madhvapathy, J. Trueb, M. Stanslaski, E. M. Higbee-Dempsey, I. Stepien, N. Ghoreishi-Haack, C. R. Haney, T.-I. Kim, Y. Huang, R. Ghaffari, A. R. Banks, T. C. Jhou, C. H. Good, J. A. Rogers, A wireless and battery-less implant for multimodal closed-loop neuromodulation in small animals. *Nat. Biomed. Eng.* **7**, 1252–1269 (2023).

5. Y. Wang, Z. Chen, B. Davis, W. Lipman, S. Xing, L. Zhang, T. Wang, P. Hafiz, W. Xie, Z. Yan, Z. Huang, J. Song, W. Bai, Digital automation of transdermal drug delivery with high spatiotemporal resolution. *Nat. Commun.* **15**, 511 (2024).

6. D. Yin, P. Wang, Y. Hao, W. Yue, X. Jiang, K. Yao, Y. Wang, X. Hang, A. Xiao, J. Zhou, L. Lin, Z. Rao, H. Wu, F. Liu, Z. Dong, M. Wu, C. Xu, J. Huang, H. Chang, Y. Fan, X. Yu, C. Yu, L. Chang, M. Li, A battery-free nanofluidic intracellular delivery patch for internal organs. *Nature* **642**, 1051–1061 (2025).

7. Z. Liu, X. Hu, R. Bo, Y. Yang, X. Cheng, W. Pang, Q. Liu, Y. Wang, S. Wang, S. Xu, Z. Shen, Y. Zhang, A three-dimensionally architected electronic skin mimicking human mechanosensation. *Science* **384**, 987–994 (2024).

8. T. Zheng, X. Xie, Q. Shi, J. Wu, C. Yu, Self-powered artificial neuron devices: Towards the all-in-one perception and computation system. *Adv. Mater.* **37**, e2416897 (2025).

9. J. Jiang, R. Zhang, M. H. Lee, W. Wu, Flexible piezo-electrocatalytic uric acid sensor. *Nano Energy* **118**, 108978 (2023).

10. S. Niu, X. Wang, F. Yi, Y. S. Zhou, Z. L. Wang, A universal self-charging system driven by random biomechanical energy for sustainable operation of mobile electronics. *Nat. Commun.* **6**, 8975 (2015).

11. Z. Xie, L. Teng, H. Wang, Y. Liu, M. Fu, J. Liang, A self-powered synchronous switch energy extraction circuit for electromagnetic energy harvesting enhancement. *IEEE Trans. Power Electron.* **38**, 9972–9982 (2023).

12. Y. Song, J. Min, Y. Yu, H. Wang, Y. Yang, H. Zhang, W. Gao, Wireless battery-free wearable sweat sensor powered by human motion. *Sci. Adv.* **6**, eaay9842 (2020).

13. H. Yu, N. Li, N. Zhao, How far are we from achieving self-powered flexible health monitoring systems: An energy perspective. *Adv. Energy Mater.* **11**, 2002646 (2021).

14. Y. Pang, S. Chen, Y. Cao, Z. Huang, X. Xu, Y. Fang, C. Cao, Self-powered multifunctional human–nachine interfaces for respiratory monitoring and smart system control. *Adv. Mater. Interfaces* **9**, 2201202 (2022).

15. D. Wang, W. Liu, L. Gu, E. Schmuck, T. Hacker, P. Chen, J. Sui, X. Wang, Instantaneous piezoelectric nanogenerator for pacemaker applications. *Nano Energy* **138**, 110828 (2025).

16. D. Kang, B.-J. Park, J.-H. Hwang, Y.-J. Kim, S.-H. Kim, H. W. Kim, K. J. Yu, J. Jeon, H. Y. Lee, Y. Chung, S. H. Nam, B.-O. Choi, S.-W. Kim, In-body current path manipulation with minimal attenuation. *Sci. Adv.* **11**, eadx5922 (2025).

17. V. Vallem, Y. Sargolzaeiaval, M. Ozturk, Y.-C. Lai, M. D. Dickey, Energy harvesting and storage with soft and stretchable materials. *Adv. Mater.* **33**, e2004832 (2021).

18. E. Istif, H. Mirzajani, Ç. Dağ, F. Mirlou, E. Y. Ozuaciksoz, C. Cakır, H. C. Koydemir, I. Yilgor, E. Yilgor, L. Beker, Miniaturized wireless sensor enables real-time monitoring of food spoilage. *Nat. Food* **4**, 427–436 (2023).

19. X. Chen, Z. Lou, X. Gao, L. Yin, S. Qin, M. Lin, F. Zhang, Y. Lu, S. Ding, R. Liu, S. Tang, S. Zhou, D. T. Dang, X. Yang, Z. Wu, Z. Zhang, H. Hu, X. Wang, Y. Zhu, Y. Xu, R. Sheng, J. Zhou, C. Lu, R. Wang, W. Yue, H. Huang, R. S. Wu, Y. Bian, G. Park, J. Cao, X. Liu, D. Luo, G. Cauwenberghs, J. Wang, S. Xu, A noise-tolerant human–machine interface based on deep learning-enhanced wearable sensors. *Nat. Sens.* **1**, 39–51 (2026).

20. Y. Xin, L. Chen, B. Sun, F. Tang, Y. Zhang, Galvanic-cell-based self-powered bioelectronic devices. *Adv. Mater.* **37**, e09169 (2025).

21. X. Hu, Z. Liu, Y. Zhang, Three-dimensionally architected tactile electronic skins. *ACS Nano* **19**, 14523–14539 (2025).

22. L. Chang, in *Proceedings of the 4th International Symposium on Semiconductor and Electronic Technology (ISSET)* (Institute of Electrical and Electronics Engineers, 2025), pp. 483–486.

23. G. M. Iodice, W. Furtner, Edge AI—An industry view. *IEEE Des. Test* **42**, 27–34 (2025).

24. T. M. Ingolfsson, V. Kartsch, L. Benini, A. Cossettini, A wearable ultra-low-power system for EEG-based speech-imagery interfaces. *IEEE Trans. Biomed. Circuits Syst.* **19**, 743–755 (2025).

25. S. Frey, G. Spacone, A. Cossettini, M. Guermandi, P. Schilk, L. Benini, V. Kartsch, BioGAP-Ultra: A modular Edge-AI platform for wearable multimodal biosignal acquisition and processing. *IEEE Trans. Biomed. Circuits Syst.* **20**, 399–415 (2026).

26. K. A. Kasper, R. Thien, T. Stuart, J. Kim, A. Bhatia, P. Gutruf, Wearable AI for on-device frailty assessment. *Nat. Commun.* **17**, 991 (2026).

27. R. Katmah, A. A. Shehhi, H. F. Jelinek, A. A. Hulleck, K. Khalaf, A systematic review of gait analysis in the context of multimodal sensing fusion and AI. *IEEE Trans. Neural Syst. Rehabil. Eng.* **31**, 4189–4202 (2023).

28. Q. Wang, H. Guan, C. Wang, P. Lei, H. Sheng, H. Bi, J. Hu, C. Guo, Y. Mao, J. Yuan, M. Shao, Z. Jin, J. Li, W. Lan, A wireless, self-powered smart insole for gait monitoring and recognition via nonlinear synergistic pressure sensing. *Sci. Adv.* **11**, eadu1598 (2025).

29. A. C. Y. Lim, P. Natarajan, R. D. Fonseka, M. Maharaj, R. J. Mobbs, The application of artificial intelligence and custom algorithms with inertial wearable devices for gait analysis and detection of gait-altering pathologies in adults: A scoping review of literature. *Digit. Health* **8**, 205520762210741 (2022).

30. D. Jarchi, J. Pope, T. K. M. Lee, L. Tamjidi, A. Mirzaei, S. Sanei, A review on accelerometry-based gait analysis and emerging clinical applications. *IEEE Rev. Biomed. Eng.* **11**, 177–194 (2018).

31. E. Ergin, S. Savci, B. Ozcan Kahraman, A. Tanriverdi, I. Ozsoy, G. Atakul, A. Volkan, S. Asilsoy, N. Uzuner, Three-axis accelerometer system for comparison of gait parameters in children with cystic fibrosis and healthy peers. *Gait Posture* **78**, 60–64 (2020).

32. M. Mancini, M. Afshari, Q. Almeida, S. Amundsen-Huffmaster, K. Balfany, R. Camicioli, C. Christiansen, M. L. Dale, L. E. Dibble, G. M. Earhart, T. D. Ellis, G. J. Griffith, M. E. Hackney, J. Hopkins, F. B. Horak, K. E. Jones, L. Ling, J. A. O'keefe, K. Kwei, G. Olivier, A. K. Rao, A. Sivaramakrishnan, D. M. Corcos, Digital gait biomarkers in Parkinson's disease: Susceptibility/risk, progression, response to exercise, and prognosis. *npj Parkinson's Dis.* **11**, 51 (2025).

33. J. M. Jasiewicz, J. H. J. Allum, J. W. Middleton, A. Barriskill, P. Condie, B. Purcell, R. C. T. Li, Gait event detection using linear accelerometers or angular velocity transducers in able-bodied and spinal-cord injured individuals. *Gait Posture* **24**, 502–509 (2006).

34. S. Fink, M. Suppanz, J. Oberzaucher, M. A. Castro, O. Fernandes, I. Alves, Gait characterization in rare bone diseases in a real-world environment – A comparative controlled study. *Gait Posture* **112**, 174–180 (2024).

35. Y. Celik, S. Stuart, W. L. Woo, A. Godfrey, Gait analysis in neurological populations: Progression in the use of wearables. *Med. Eng. Phys.* **87**, 9–29 (2021).

36. Y. Yun, S. Jang, S. Cho, S. H. Lee, H. J. Hwang, D. Choi, Exo-shoe triboelectric nanogenerator: Toward high-performance wearable biomechanical energy harvester. *Nano Energy* **80**, 105525 (2021).

37. M. H. Albuqami, E. Kovalska, K. S. Sadanandan, M. S. Alghamdi, A. I. S. Neves, S. Russo, M. F. Craciun, Integration of two-dimensional WS2 in flexible textile triboelectric nanogenerators via electronic dyeing for self-powered sensing. *Nanoscale* **17**, 12080–12086 (2025).

38. Y. Zi, H. Guo, Z. Wen, M.-H. Yeh, C. Hu, Z. L. Wang, Harvesting low-frequency (<5 Hz) irregular mechanical energy: A possible killer application of triboelectric nanogenerator. *ACS Nano* **10**, 4797–4805 (2016).

39. F. Jiang, G. Thangavel, J. P. Lee, A. Gupta, Y. Zhang, J. Yu, T. Yokota, K. Yamagishi, Y. Zhang, T. Someya, P. S. Lee, Self-healable and stretchable perovskite-elastomer gas-solid triboelectric nanogenerator for gesture recognition and gripper sensing. *Sci. Adv.* **10**, eadq5778 (2024).

40. G. Xu, H. Wang, G. Zhao, J. Fu, K. Yao, S. Jia, R. Shi, X. Huang, P. Wu, J. Li, B. Zhang, C. K. Yiu, Z. Zhou, C. Chen, X. Li, Z. Peng, Y. Zi, Z. Zheng, X. Yu, Self-powered electrotactile textile haptic glove for enhanced human-machine interface. *Sci. Adv.* **11**, eadt0318 (2025).

41. A. Delbani, D. Galayko, M. Kane, P. Basset, Road bump triboelectric generator excited by a small number of actuations for powering Bluetooth communications. *APL Electron. Devices* **1**, 016114 (2025).

42. Z. Rao, F. Ershad, Y.-S. Guan, F. C. Paccola Mesquita, E. C. Da Costa, M. A. Morales-Garza, A. Moctezuma-Ramirez, B. Kan, Y. Lu, S. Patel, H. Shim, K. Cheng, W. Wu, T. Haideri, X. L.

Lian, A. Karim, J. Yang, A. Elgalad, C. Hochman-Mendez, C. Yu, Ultrathin rubbery bio-optoelectronic stimulators for untethered cardiac stimulation. *Sci. Adv.* **10**, eadq5061 (2024).

43. P. Cheng, S. Dai, Y. Liu, Y. Li, H. Hayashi, R. Papani, Q. Su, N. Li, Y. Dai, W. Liu, H. Hu, Z. Liu, L. Jin, N. Hibino, Z. Wen, X. Sun, S. Wang, An intrinsically stretchable power-source system for bioelectronics. *Device* **2**, 100216 (2024).

44. C. Han, M. Jin, F. Dong, P. Xu, X. Jiang, S. T. Cai, Y. Jiang, Y. Zhang, Y. Fang, S. Niu, Interpretable machine learning for evaluating nanogenerators' structural design. *ACS Nano* **19**, 14456–14466 (2025).

45. T. Chen, C. Guestrin, in *Proceedings of the the 22nd ACM SIGKDD International Conference on Knowledge Discovery and Data Mining* (Association for Computing Machinery, 2016), pp. 785–794.

46. W. L. Haskell, I.-M. Lee, R. R. Pate, K. E. Powell, S. N. Blair, B. A. Franklin, C. A. Macera, G. W. Heath, P. D. Thompson, A. Bauman, American College of Sports Medicine, American Heart Association, Physical activity andpublic health: Updated recommendation for adults from the American College of Sports Medicine and the American Heart Association. *Circulation* **116**, 1081–1093 (2007).

47. D. R. Bassett, J. A. Vachon, A. O. Kirkland, E. T. Howley, G. E. Duncan, K. R. Johnson, Energy cost of stair climbing and descending on the college alumnus questionnaire. *Med. Sci. Sports Exerc.* **29**, 1250–1254 (1997).

48. S. M. Lundberg, S.-I. Lee, in *Proceedings of the 31st Conference on Neural Information Processing Systems (NIPS)* (Curran Associates Inc., 2017), pp. 4765–4774.

49. S. M. Lundberg, G. Erion, H. Chen, A. Degrave, J. M. Prutkin, B. Nair, R. Katz, J. Himmelfarb, N. Bansal, S.-I. Lee, From local explanations to global understanding with explainable AI for trees. *Nat. Mach. Intell.* **2**, 56–67 (2020).

50. X. Tang, J. Zhang, Y. He, X. Zhang, Z. Lin, S. Partarrieu, E. B. Hanna, Z. Ren, H. Shen, Y. Yang, X. Wang, N. Li, J. Ding, J. Liu, Explainable multi-task learning for multi-modality biological data analysis. *Nat. Commun.* **14**, 2546 (2023).

51. W. F. Fadel, J. K. Urbanek, S. R. Albertson, X. Li, A. K. Chomistek, J. Harezlak, Differentiating between walking and stair climbing using raw accelerometry data. *Stat. Biosci.* **11**, 334–354 (2019).

52. S. F. Castiglia, D. Trabassi, A. Tatarelli, A. Ranavolo, T. Varrecchia, L. Fiori, D. Di Lenola, E. Cioffi, M. Raju, G. Coppola, P. Caliandro, C. Casali, M. Serrao, Identification of gait unbalance and fallers among subjects with cerebellar ataxia by a set of trunk acceleration-derived indices of gait. *Cerebellum* **22**, 46–58 (2023).

53. S. Viteckova, P. Kutilek, Z. Svoboda, R. Krupicka, J. Kauler, Z. Szabo, Gait symmetry measures: A review of current and prospective methods. *Biomed. Signal Process. Control* **42**, 89–100 (2018).

54. T. A. Najafi, J. A. M. Calero, J. Thevenot, B. Duc, S. Albini, A. Amirshahi, H. Taji, M. J. B. Beneyto, A. Affanni, D. Atienza, VersaSens: An extendable multimodal platform for next-generation Edge-AI wearables. *IEEE Trans. Circuits Syst. Artif. Intell.* **1**, 83–96 (2024).

55. G. Dion, A. Tessier-Poirier, L. Chiasson-Poirier, J.-F. Morissette, G. Brassard, A. Haman, K. Turcot, J. Sylvestre, In-sensor human gait analysis with machine learning in a wearable microfabricated accelerometer. *Commun. Eng.* **3**, 48 (2024).
